# Ambiphilic Reactivity of Iridium Complexes with *N*‑Heterocyclic Vinylidene Ligands

**DOI:** 10.1021/jacs.5c04371

**Published:** 2025-05-28

**Authors:** Tak Hin Wong, Paul Varava, Farzaneh Fadaei-Tirani, Rosario Scopelliti, Kay Severin

**Affiliations:** Institut des Sciences et Ingénierie Chimiques, 27218 École Polytechnique Fédérale de Lausanne (EPFL), 1015 Lausanne, Switzerland

## Abstract

Room-temperature stable diazoolefins provide access to a unique class of carbon-donor ligands: *N*-heterocyclic vinylidenes. Herein, we describe iridium complexes with terminal *N*-heterocyclic vinylidene ligands. Cationic complexes of type [Cp*IrClL]­X (X = SbF_6_, OTf; L = *N*-heterocyclic vinylidene) were obtained by reaction of (Cp*IrCl_2_)_2_ with an *N*-heterocyclic diazoolefin, followed by chloride abstraction. Reduction instead of chloride abstraction gave a neutral Cp*IrL complex with a “pogo stick” geometry. Crystallographic analyses of the complexes revealed very short iridium–carbon bonds, suggesting partial carbyne character. The vinylidene ligands display ambiphilic reactivity. The cationic complex forms covalent adducts with halides and pseudohalides, whereas the neutral complex reacts with electrophiles. These transformations generate rare carbon-donor species, including anionic *N*-heterocyclic olefins with pronounced carbene character or a side-on bound alkylidene ketene.

## Graphical abstract

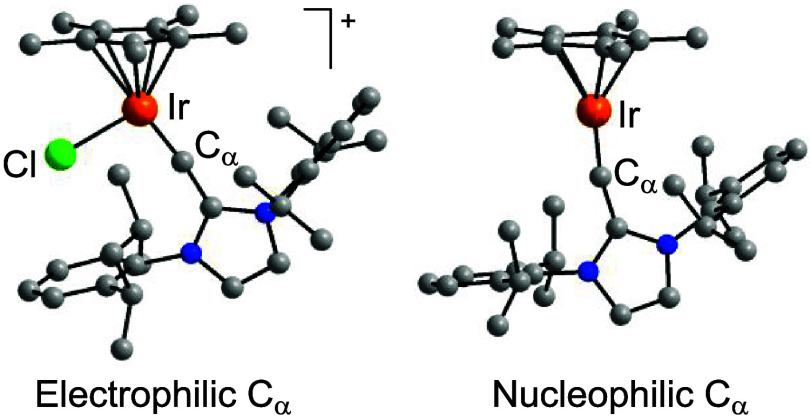

## Introduction

2-Methyleneimidazolines feature a highly polarized exocyclic double bond. As a result, they can form stable complexes with main group Lewis acids and with metal complexes (Scheme 1a). Pioneering work in this area was performed by Kuhn and co-workers. They showed that 1,3,4,5-tetramethyl-2-methyleneimidazoline can coordinate to BH_3_, to Mo­(CO)_5_, and to lanthanide complexes.^1^ Today, the coordination chemistry of 2-methyleneimidazolines is well-developed, and applications in catalysis have been reported.^2^ Other *N*-heterocycles with exocyclic alkylidene groups display a similar Lewis basicity, and compounds of type (*N*-heterocycle)=CRR′ are nowadays referred to as ‘*N*-heterocyclic olefins’ (NHOs).^2^

**Scheme 1:**
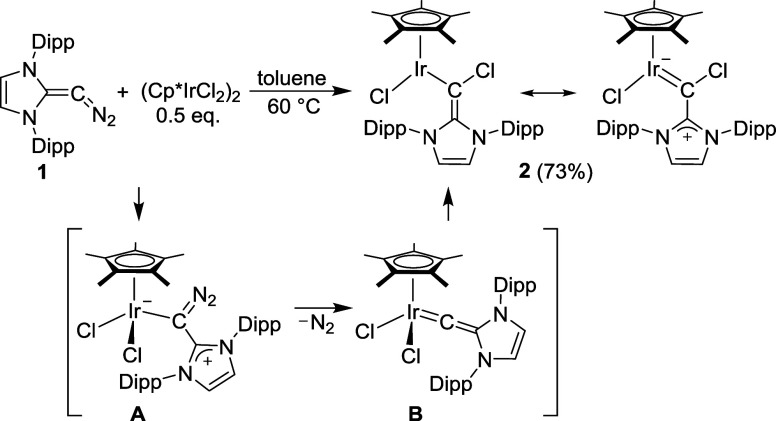
Synthesis of Complex **2** and the Proposed Intermediates A and B

The formal oxidation of NHOs gives *N*-heterocyclic vinylidenes (Scheme 1b). In contrast to the rich chemistry of NHOs, the coordination chemistry of *N*-heterocyclic vinylidenes is largely unexplored. The scarcity of studies in this area is not surprising, given the lack of suited precursors for *N*-heterocyclic vinylidene ligands.^3^ The situation changed in 2021, when the synthesis of thermally stable *N*-heterocyclic diazoolefins was reported by Hansmann and co-workers^4^ and by our group.^5,6^ The conversion of *N*-heterocyclic diazoolefins into vinylidenes requires loss of dinitrogen, which can be induced by photochemical or thermal activation.^4–7^

Due to the electron-donating nature of the azolylidene fragment, *N*-heterocyclic vinylidenes are expected to be strong C-donor ligands. This proposition was substantiated by the synthesis of a VCl_3_ complex with an imidazole-based *N*-heterocyclic vinylidene ligand.^8^ A structural analysis of this complex revealed a very short V–C bond. A different situation was encountered for a CuCl complex.^9^ An X-ray diffraction (XRD) analysis was achieved by *in crystallo* photolysis of a diazoolefin adduct at low temperature. The Cu–C bond length was found to be in the range of a single bond, and DFT calculations suggested a triplet ground state. In solution, the CuCl complex displayed high reactivity, and attempts to isolate it were not successful.

Intrigued by these first results, we have explored if *N*-heterocyclic vinylidene complexes can be obtained with other transition metals.^10,11^ Below, we describe the syntheses, the structures, and the reactivity of iridium complexes with terminal *N*-heterocyclic vinylidene ligands (Figure 1c). The strong donor character of the ligands is corroborated by the isolation of a low-coordinate Cp*IrL complex with a “pogo stick” geometry. Moreover, we show for the first time that *N*-heterocyclic vinylidene ligands can engage in coupling reactions with nucleophiles and electrophiles. The resulting complexes feature unusual C-donor ligands such as anionic *N*-heterocyclic olefins and alkylidene ketenes.

**Figure 1:**
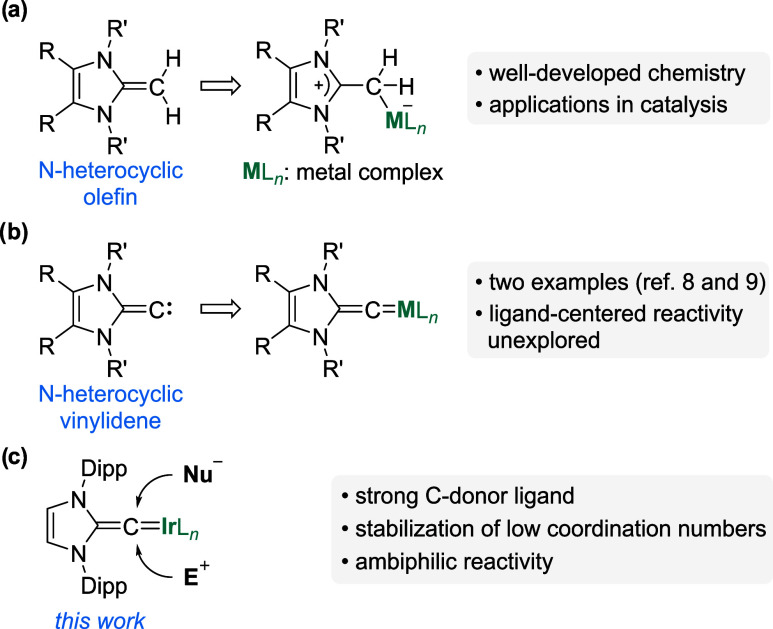
Imidazole-based *N*-heterocyclic olefins represent versatile C-donor ligands (a). In contrast, the coordination chemistry of *N*-heterocyclic vinylidenes is largely unexplored (b). Herein, we describe the syntheses and the reactivity of iridium complexes with *N*-heterocyclic vinylidene ligands (Dipp = 2,6-C_6_H_3_ *i*Pr_2_) (c).

## Results and Discussion

For our investigations, we used a diazoolefin with 2,6-C_6_H_3_ *i*Pr_2_ (Dipp) wing-tip groups (**1**). This compound can be obtained from the corresponding NHO by reaction with the diazo transfer reagent nitrous oxide,^12^ as described earlier.^5^

When the diazoolefin **1** was combined with 0.5 equiv of (Cp*IrCl_2_)_2_ in deuterated toluene, a mixture of products was obtained, as evidenced by ^1^H NMR spectroscopy (see the Supporting Information, Figure S50). A cleaner reaction was observed when a solution of **1** in toluene was added to a solution of (Cp*IrCl_2_)_2_ at 60 °C. Under these conditions, complex **2** was formed as the main product (Scheme 1). Isolation of complex **2** was achieved by extraction with pentane, followed by solvent removal (yield: 73%). Slow evaporation of a concentrated solution of **2** in pentane gave single crystals, which were analyzed by XRD. The results revealed that a complex with an *N*-heterocyclic vinyl ligand had formed (Figure 2).

**Figure 2:**
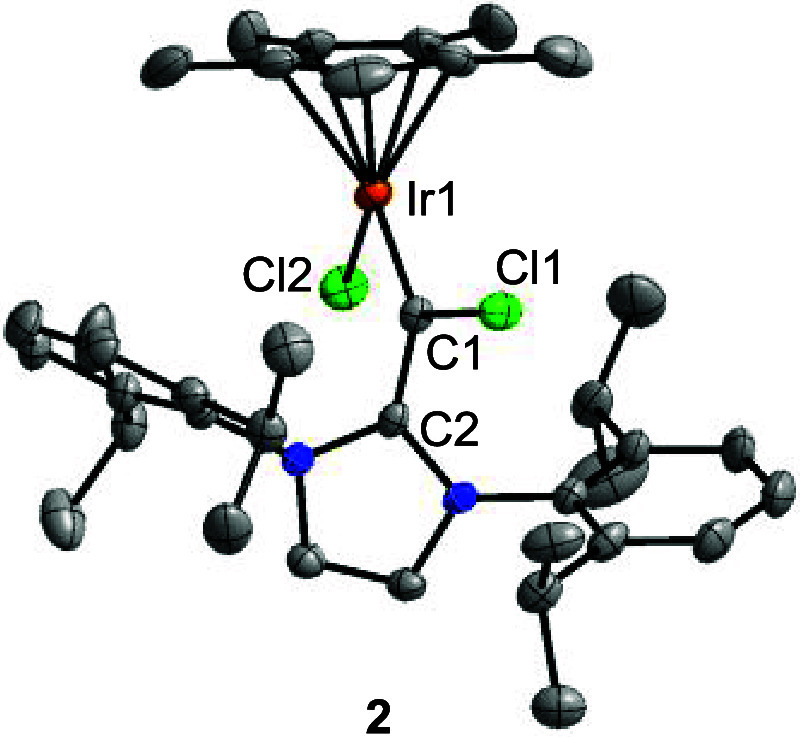
Molecular structure of complex **2** in the crystal with thermal ellipsoids at 50% probability. Selected bond lengths (Å): Ir1–C1 1.890(3), Ir1–Cl2 2.3818(12), C1–Cl1 1.815(3), C1–C2 1.448(5). Only one of the two crystallographically independent molecules is shown. Hydrogen atoms are omitted for clarity.

Thus far, there are only a few examples of transition metal complexes with *N*-heterocyclic vinyl ligands (also referred to as ‘anionic *N*-heterocyclic olefins’). Rivard and co-workers have prepared Zn, Ti, Zr, and Hf complexes by salt metathesis reactions involving a lithiated *N*-heterocyclic olefin.^13^ A different approach was reported by Fischer and co-workers. They demonstrated the formation an *N*-heterocyclic vinyl ligand in the coordination sphere of titanium by formal intramolecular proton transfer to a pentafulvene ligand.^14^ For the resulting Ti complex, a significant contribution of an alkylidene resonance structure was noted. A similar situation is found for complex **2** (Scheme 1). There are two crystallographically independent molecules in the unit cell. The lengths of the Ir–C bonds, 1.890(3) Å and 1.893(4) Å, are in the range found for Ir carbene complexes,^15^ and the lengths of the C–C bonds between the metalated C atom and the heterocycle, 1.448(5) and 1.452(5) Å, indicate significant single bond character. It is also worth noting that complex **2** is structurally similar to the phosphinidenide complex Cp*IrCl­(P=IDipp) (IDipp = 1,3-bis­(2,6-diisopropylphenyl)­imidazolin-2-ylidene), previously reported by Tamm and co-workers.^16^

A computational analysis of complex **2** substantiated the description as a carbene complex. The geometry of **2** could be reproduced with good accuracy by density functional theory (DFT) calculations at M062X/LanL2DZ,6–31G­(d,p) level of theory (for details, see the Supporting Information, section 7). A natural bond orbital (NBO) analysis of the optimized structure revealed two NBOs associated with the Ir–C bond: one of σ-type and one of π-type character, with occupation numbers of 1.87 and 1.85, respectively. The Wiberg bond index (WBI) of the Ir–C bond is 1.24.

The ^1^H NMR spectrum of complex **2** in *d*_8_-THF was found to be temperature dependent (see the Supporting Information, Figure S6). At 60 °C, one broad signal for the CHMe_2_ protons of the four isopropyl groups was observed. This signal splits into two at temperatures below −10 °C. These results imply restricted rotation of the N–C_Dipp_ bonds.

To allow for an unambiguous assignment of the ^13^C NMR signal of the metalated carbon atom, we repeated the synthesis of complex **2** with a ^13^C-labeled diazoolefin (for details, see the Supporting Information). The labeling showed that C_α_ resonates at 128.7 ppm (*d*_8_-THF, – 50 °C). The ^1^ *J*(Ir^13^C–^13^C) coupling constant of 62 Hz indicates a C_sp^2^_–C_sp^2^_ bond.

The formation of complex **2** could proceed via the diazoolefin adduct **A** (Scheme 1). Similar adducts have been reported for reactions of **1** with CuCl, AuCl, RhCl­(CO)_2_, PdCl­(allyl), or Cr­(CO)_5_.^5,9,17^ Loss of dinitrogen would provide intermediate **B**. Chloride migration from Ir to C_α_ then gives the final product **2**.

The C–Cl bond formation could be reversed by the addition of AgSbF_6_ or Ph_2_I­(OTf), respectively (Scheme 2).^18^ The resulting complexes **3** and **4** represent the first examples of iridium complexes with *N*-heterocyclic vinylidene ligands.

**Scheme 2:**
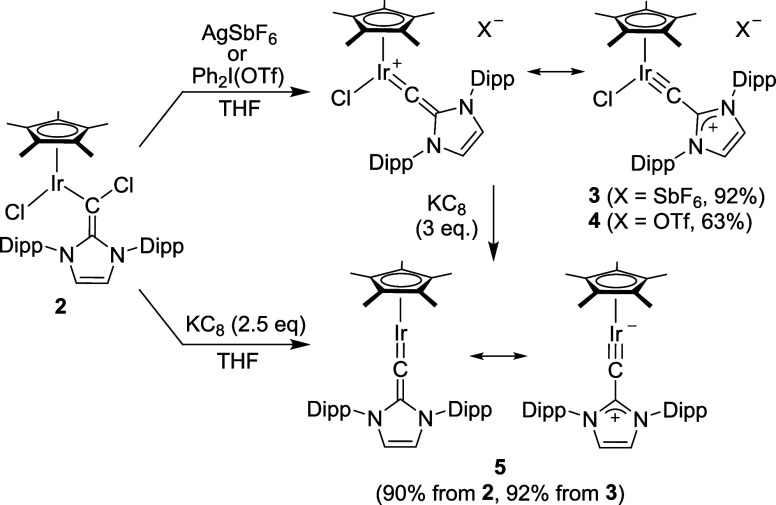
Synthesis of the Complexes **3–5**

In terms of spectroscopic properties, we were particularly interested in the chemical shift of the ^13^C NMR signal of the metalated carbon atom of the vinylidene ligand. ^13^C labeling revealed that the NMR signal of the metalated C atom resonates at 303.8 ppm. A closely related value, 296.9 ppm, was reported by Luecke and Bergman for the iridium carbyne complex (η^5^-C_5_Me_4_Et)­Ir­(PMe_3_)­(CPh).^19^

The complexes **3** and **4** were both analyzed by single-crystal X-ray diffraction (Figure 3). As expected, the structural parameters for the cationic Ir complexes are similar. The vinylidene ligand is coordinated to the metal in a nearly linear fashion, with Ir–C–C bond angles of 172.4(2)° for complex **3** and 172.3(3)° for complex **4**. Both complexes exhibit very short Ir–C bonds with lengths of 1.750(3) Å (**3**) and 1.731(4) Å (**4**), respectively. These values are small when compared to what has been reported for nonheterocyclic iridium vinylidene complexes (1.76–1.83 Å).^20^ The above-mentioned carbyne complex (η^5^-C_5_Me_4_Et)­Ir­(PMe_3_)­(CPh) shows an Ir–C bond distance of 1.734(6) Å.^19^ This comparison suggests that the Ir–C bonds in complexes **3** and **4** possess triple-bond character, consistent with the resonance structures depicted in Scheme 2.

**Figure 3:**
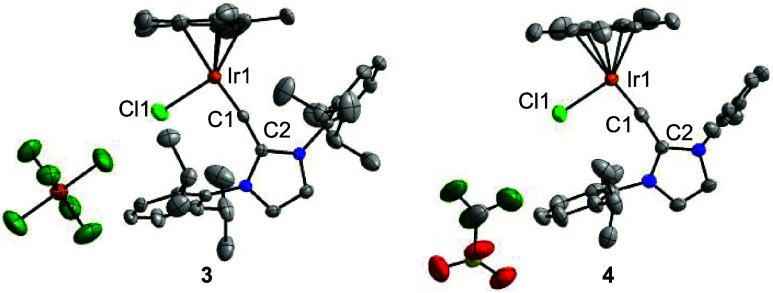
Molecular structures of the complexes **3** and **4** in the crystal with thermal ellipsoids at 50% probability. Selected bond lengths (Å): **3**: Ir1–C1 1.750(3), Ir1–Cl1 2.3065(9), C1–C2 1.411(4); **4**: Ir1–C1 1.731(4), Ir1–Cl1 2.3155(11), C1–C2 1.424(5). For complex **4**, only one of the two crystallographically independent molecules is shown. Hydrogen atoms and cocrystallized solvent molecules are not shown for clarity.

The description of **3** and **4** as carbyne complexes is supported by computational results (for details, see the Supporting Information, section 7). An NBO analysis of the optimized structure of the cationic part of complex **3**/**4** revealed one σ-type NBO and two π-type NBOs for the Ir–C bond (occupation numbers: 1.96, 1.90, and 1.88). The WBI of the Ir–C bond is 1.96.

Reduction of complex **2** with 2.5 equiv of KC_8_ gave the vinylidene complex **5** in high yield (Scheme 2). Alternatively, complex **5** is accessible by reduction of **3** with KC_8_. Complex **5** shows a “pogo stick” geometry, as evidenced by single-crystal XRD (Figure 4). Similar to what was observed for the cationic complexes **3** and **4**, the vinylidene ligand in **5** is coordinated to the metal in a nearly linear fashion, with an Ir–C–C bond angle of 167.0(6)°. The Ir–C bond lengths of 1.747(7) Å indicates again a partial triple bond character. Accordingly, we found three NBOs associated with the Ir–C bond and a high WBI of 1.97. A notable difference between the “pogo stick” complex **5** and the chloro complexes **3**/**4** is the calculated charge density at C_α_, as revealed by a natural population analysis (NPA). For complex **5**, we calculated a value of −0.46, whereas a value of 0.01 was obtained for **3**/**4**.

**Figure 4:**
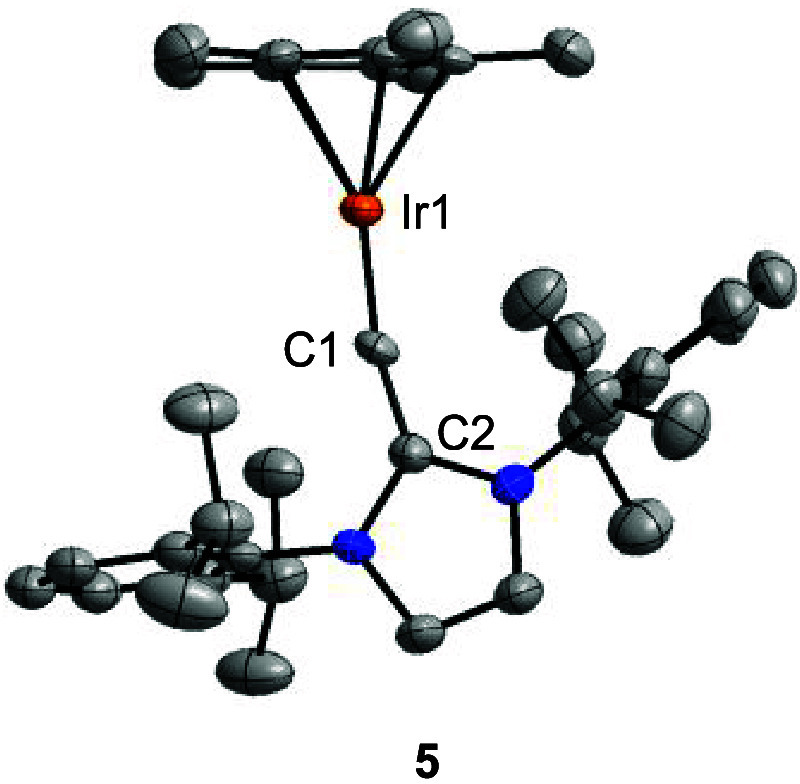
Molecular structure of complex **5** in the crystal with thermal ellipsoids at 50% probability. Selected bond lengths (Å) and angles (°): Ir1–C1 1.744(7), C1–C2 1.402(10), Ir1–C1–C2 167.0(6). Hydrogen atoms are not shown for clarity.

The ^13^C NMR signal of the metalated C_α_ atom in complex **5** resonates at 167.7 ppm. The ^1^ *J*(Ir^13^C–^13^C) coupling constant of 59 Hz is similar to what was found for complex **2**.

Cp*Ir complexes with a “pogo stick” geometry have been reported with sterically demanding imido^21^ or hydrazido ligands.^22^ The overall geometry of these Cp*Ir­(NR) complexes is comparable to that of **5**, with a nearly linear coordination of the NR ligands.

With two distinct vinylidene complexes at hand, we studied their reactivity. Since complex **3** was obtained by chloride abstraction, we suspected that we could convert it back into complex **2** by addition of chloride. Indeed, upon mixture of (NBu_4_)Cl with complex **3** in THF, complex **2** was formed (Scheme 3). The chloride adduct could be separated from the salt byproduct by solvent removal followed by extraction with pentane (isolated yield: 72%). A nucleophilic addition to the C_α_ atom could also be achieved with fluoride, cyanide, and isocyanide to give the complexes **6**–**8**.

**Scheme 3:**
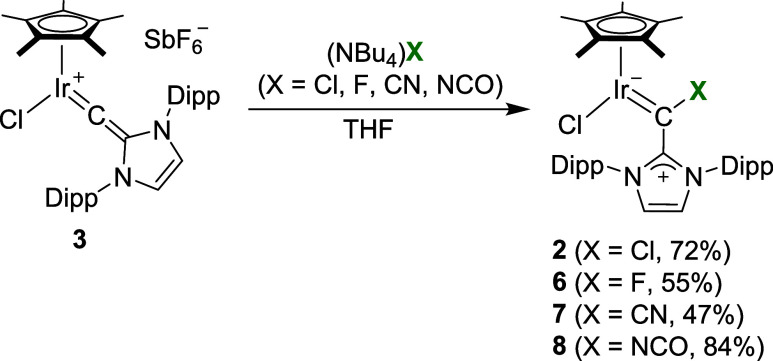
Synthesis of the Complexes **2** and **6–8**

The reactions with halides and pseudohalides demonstrate that the metalated carbon of the vinylidene ligand in complex **3** is electrophilic. The experimental data are in line with computational results, which show that complex **3** displays a low-lying LUMO with a large coefficient at C_α_ (for details, see the Supporting Information, section 7).

The adducts **6**–**8** were all characterized by single-crystal XRD, and the solid-state structures are depicted in Figure 5. The structural data imply that the compounds are best described as carbene complexes, and not as vinyl complexes, with short Ir–C bonds (1.81–1.92 Å) and long IrC–C bonds (1.43–1.50 Å; for details, see the Supporting Information, Table S11). Moreover, the plane defined by the imidazolium heterocycle is strongly tilted with respect to the plane defined by the Ir–C–X atoms, as evidenced by Ir–C–C–N torsion angles of 51° or higher. For a vinyl complex, these angles would be expected to be close to zero.

**Figure 5:**
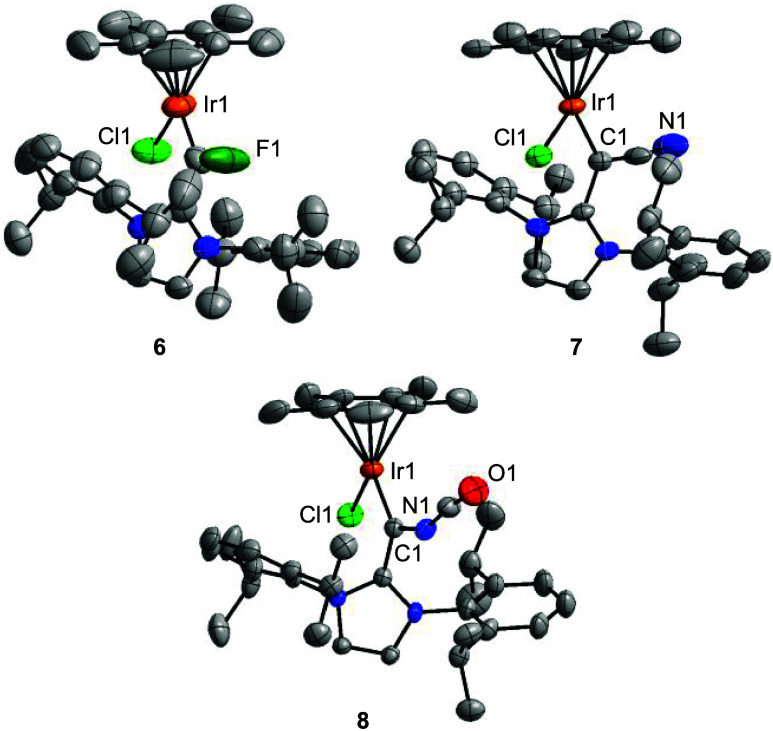
Molecular structures of the complexes **6**–**8** in the crystal with thermal ellipsoids at 50% probability. Hydrogen atoms are not shown for clarity.

Reactivity studies with the “pogo stick” complex **5** revealed that C_α_ is nucleophilic, rather than electrophilic. The reaction of complex **5** with methyl iodide resulted in alkylation of C_α_ (Scheme 4). The corresponding adduct **9** was found to be a salt, as evidenced by a crystallographic analysis (Figure 6). Instead of iodide, one Dipp group is bound to the metal via its π-system (“π-face donation”).^23^ A related coordination of one Dipp group has previously been observed for Rh^I^ and Ir^I^ complexes with thione and selenone ligands derived from *N*-heterocyclic carbenes^24^ or with an anionic diazoolefin ligand.^25^ In solution, complex **9** displayed low stability, and a characterization by ^13^C NMR spectroscopy was not achieved.

**Figure 6:**
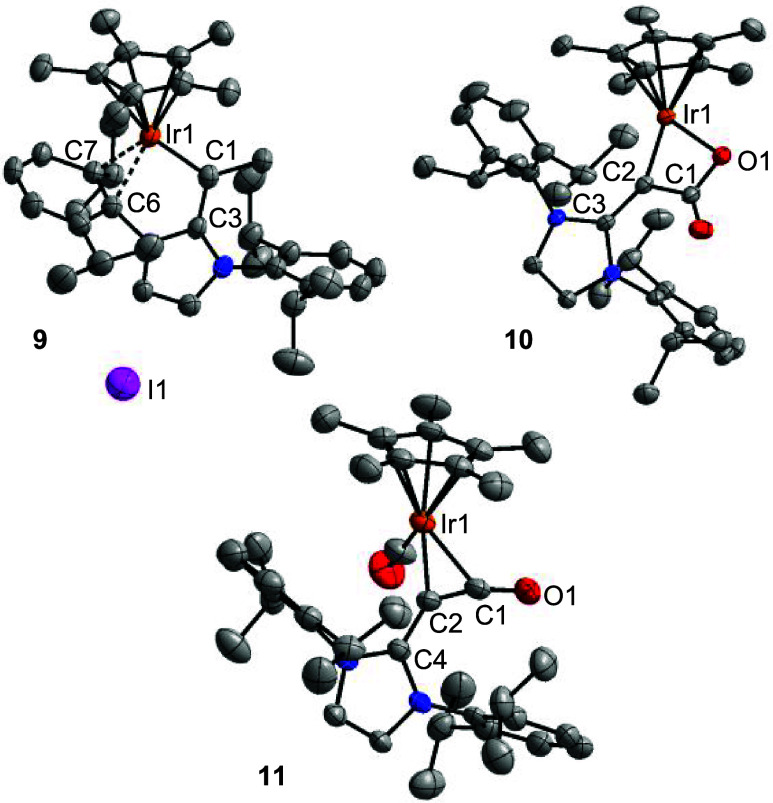
Molecular structures of the complexes **9**–**11** in the crystal with thermal ellipsoids at 50% probability. Hydrogen atoms are not shown for clarity. Selected bond lengths (Å): **9**: Ir1–C1 1.942(5), Ir1–C6 2.150(5), Ir1–C7 2.242(5), C1–C3 1.435(7); **10**: Ir1–O1 2.091(2), Ir1–C2 1.946(3), O1–C1 1.320(4), C1–C2 1.473(4), C2–C3 1.436(4); **11**: Ir1–C1 2.142(10), Ir1–C2 2.096(9), O1–C1 1.224(12), C1–C2 1.302(13), C2–C4 1.402(12).

**Scheme 4:**
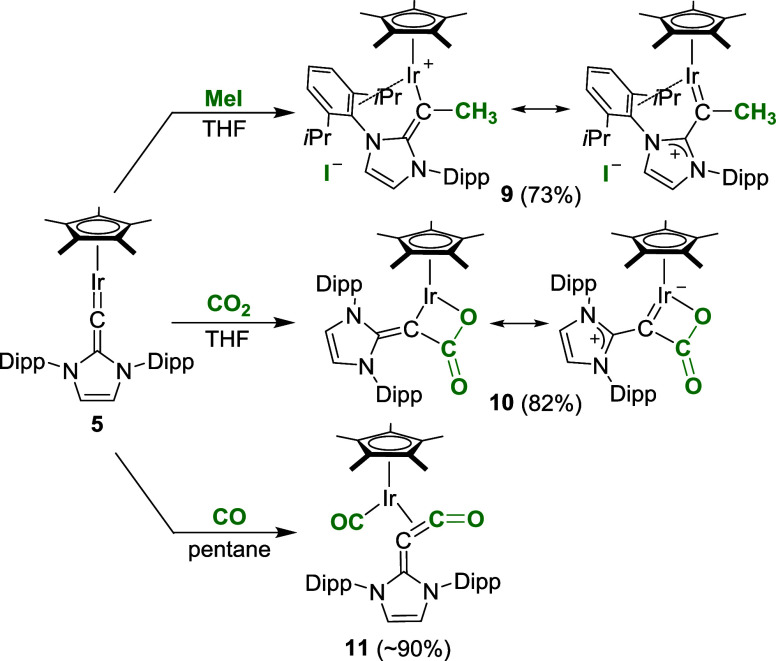
Synthesis of the Complexes **9–11**

Further evidence for the nucleophilic character of complex **5** was its ability to activate CO_2_. When a solution of complex **5** in pentane was exposed to 1 atm of CO_2_, complex **10** was obtained in high yield (Scheme 4). A single-crystal XRD analysis of **10** revealed the presence of a 4-membered C,O-chelate ligand resulting from coupling of C_α_ with the carbon atom of CO_2_ (Figure 6).

Upon addition of 1 atm of CO, complex **5** converted into the carbonyl complex **11** (Scheme 4), along with minor amounts of the known complex Cp*Ir­(CO)_2_.^26^ In addition to a terminal CO ligand, complex **11** features a side-on-bound alkylidene ketene ligand (Figure 6). *N*-Heterocyclic alkylidene ketenes are isolable, room-temperature stable compounds, which can be obtained by N_2_→CO exchange from *N*-heterocyclic diazoolefins.^27–29^ We have previously shown that these heterocumulenes can act as κ^1^-C-donor ligands for transition metals.^28^ A side-on η^2^-coordination, as observed for complex **11**, is unprecedented.

In its ‘free’ form, the alkylidene ketene ligand of complex **11** shows a bent structure, with a C–C–CO angle of 160.1(12)° and a C–C–O angle of 175.5(3)°.^28^ The side-on coordination to the Cp*Ir­(CO) fragment accentuates the bending. For crystalline **11**, one finds a C–C–CO angle of 133.5(9)° and a C–C–O angle of 158.4(11)°.

The depiction of complex **11** as a side-on olefin complex is supported by the similar Ir–C1/2 distances (2.142(10) and 2.096(9) Å), and by the pronounced double bond character of the bond between C1 and C2 (1.302(13) Å; WBI = 1.31). However, the bonding situation in compound **11** is complex, as indicated by the partial single bond character of the bond between C2 and C4 (1.402(12) Å), and the pyramidalization of C2 (the sum of the angles is 348.5°). A more detailed discussion of relevant valence formulas is given in the Supporting Information (section 7).

The reactivity of the “pogo stick” complex **5** matches closely what has been reported for Bergman’s imido complex Cp*Ir­(N*t*Bu): a reaction with MeI was found to result in alkylation of the N atom, a reaction with CO_2_ gave the N,O-chelate Cp*Ir­(O_2_CN*t*Bu), and the addition of CO resulted in the formation of a carbonyl complex with a side-on bound *t*BuNCO ligand.^21b,21c^ The comparison suggests that *N*-heterocyclic vinylidenes can act as structural and functional analogs of imido ligands.

## Conclusions

Thus far, there is very limited information about the coordination chemistry of *N*-heterocyclic vinylidene ligands, and the reactivity of these ligands is unexplored. Herein, we report two types of iridium complexes with terminal *N*-heterocyclic vinylidene ligands: the cationic complexes **3** and **4** and the neutral complex **5**. The cationic complex **3** reacts with halides and pseudohalides to give neutral adducts via C_α_–X coupling reactions. This reactivity is akin to what was reported for Fischer carbyne complexes.^30^ The halide and pseudohalide adducts **2** and **6**–**8** feature unusual C-donor ligands, which can be described as anionic *N*-heterocyclic olefins with strong carbene character.

A completely different reactivity was observed for complex **5**. The metalated carbon in **5** is nucleophilic, as evidenced by reactions with MeI, CO_2_, and CO. In terms of structure and reactivity, complex **5** closely resembles Bergman’s seminal imido complex Cp*Ir­(N*t*Bu). The similarity suggests that complex **5** can be described as an Ir^III^ complex with a dianionic alkenylidene ligand. Accordingly, the two-electron reduction that converts complex **3** into complex **5** is ligand-centered.

Overall, we have shown that *N*-heterocyclic vinylidene ligands are well suited to stabilize low-coordinate Cp*Ir complexes. The reactivity of the vinylidene ligands is controlled by the redox state of the complexes. In the reduced form, *N*-heterocyclic vinylidenes can serve as structural and functional analogs of imido ligands. The possibility to use *N*-heterocyclic vinylidenes as surrogates for imido ligands represents an interesting perspective for future studies, because imido ligands are found in a variety of transition metal catalysts.^31^

## Supplementary Material



## Notes

Experimental details; spectroscopic data; structural data, and computational details (PDF)
