# Supplementary material for: Ambiphilic Reactivity of Iridium Complexes with N‑Heterocyclic Vinylidene Ligands
Source: J Am Chem Soc. 2025 May 28;147(23):19911–7. doi: 10.1021/jacs.5c04371 (PMC12164273; doi:10.1021/jacs.5c04371)
Supplement: Supplementary file 1 [file ja5c04371_si_001.pdf]

Supporting Information

Ambiphilic Reactivity of Iridium Complexes with *N*-Heterocyclic Vinylidene Ligands

Tak Hin Wong,[a] Paul Varava,[a] Farzaneh Fadaei-Tirani,[a] Rosario Scopelliti,[a] and Kay Severin*[a]

[a] Institut des Sciences et Ingénierie Chimiques, Ecole Polytechnique Fédérale de Lausanne (EPFL), 1015 Lausanne, Switzerland, e-mail: kay.severin@epfl.ch

Table of Contents

	Page
1. General	S2
2. Synthesis of the complexes **2** – **11**	S4
3. NMR spectra	S17
4. Additional NMR experiment	S42
5. IR spectra	S43
6. Single crystal X-ray analyses	S44
7. Computational details	S76
8. References	S98

1. General

Unless stated otherwise, the reactions were performed under an atmosphere of dry dinitrogen using a glovebox. All the non-deuterated solvents were purified by using an Innovative Technology SPS solvent system. THF (non-stabilized) was taken from the solvent system and mixed with freshly dried 4 Å molecular sieves (dried from a diffusion pump at 200 °C overnight) overnight before use. Deuterated solvents were purified by storing them over molecular sieves (4 Å) for 24 h, before degassing by three freeze-pump-thaw cycles. The reagents were obtained from commercial sources and used directly in glovebox after flushing for 10 minutes. N_2O (99.999%) was purchased from Air-liquide. CO_2 (99.999%) and CO (99.95%, N47 Bt-S 10/200) were purchased from Carbagas. ^{13}CO (93.13% ^{13}C) was purchased from UPLC and transferred to a flask equipped with a J-Young valve containing activated 3 Å molecular sieves prior to use. Precise amounts of labelled gases were added to NMR tubes equipped with a J-Young valve using a short connector of known volume adapted on a Schlenk line equipped with a pressure sensor.

All analytical measurements were performed at 25 °C, if not stated otherwise. The NMR spectra were measured on a Bruker Avance DPX-400 (^1H: 400 MHz), Bruker Avance IIIHD-600 (^1H: 600 MHz), or Bruker Avance II (^1H: 800 MHz) spectrometer with BBFOz ATMA probe. Chemical shifts are given in parts per million (ppm) relative to tetramethylsilane (TMS).

Mass spectrometry analyses were performed on a LTQ Orbitrap FTMS instrument (LTQ Orbitrap Elite FTMS, Thermo Scientific, Bremen, Germany) operated in the positive mode coupled with a robotic chip-based nano-ESI source (TriVersa Nanomate, Advion Biosciences, Ithaca, NY, U.S.A.). A standard data acquisition and instrument control system was utilized (Thermo Scientific) whereas the ion source was controlled by Chipsoft 8.3.1 software (Advion BioScience). Samples were loaded onto a 96-well plate (Eppendorf, Hamburg, Germany) within an injection volume of 5 µl. The experimental conditions for the ionization voltage were +1.4 kV and the gas pressure was set at 0.30 psi. The temperature of ion transfer capillary was 80 °C. FTMS spectra were obtained in the 100-1000 *m/z* range in the reduce profile mode with a resolution set to 120,000. In all spectra one microscan was acquired with a maximum injection time value of 1000 ms.

FT-IR spectra of powdered bulk samples were recorded on a Perkin-Elmer Spectrum One instrument using the ATR (attenuated total reflection) technique with diamond-anvil configuration.

The diazoolefin starting material **1**[1] and KC_8[2] were prepared according to the procedures described in the literature.

2. Synthesis of the complexes 2 – 11

Warning: Although we have not encountered any problems during the synthesis of complex **2**, it should be noted that diazo compounds can liberate dinitrogen. Hence, we recommend that special safety precautions are taken when synthesizing (or working with) diazoolefin **1**.

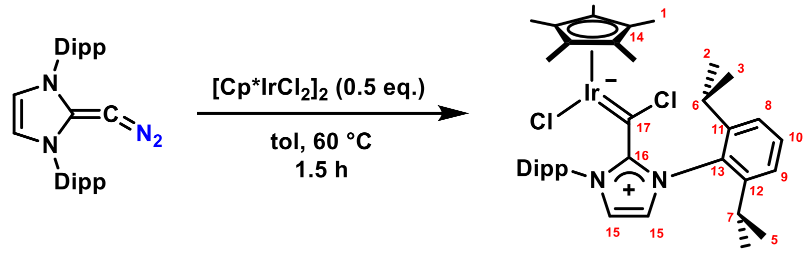

1 **2** 73%

Complex 2: A solution of diazoolefin **1** (70.0 mg, 163 μmol) in toluene (0.5 mL) was added dropwise under stirring to a solution of $[Cp^*IrCl_2]_2$ (65.1 mg, 82 μmol) in toluene (0.5 mL) at 60 °C. The mixture was stirred at 60 °C for 1.5 h. The mixture was allowed to cool down to rt and the solvent was removed under vacuum. The crude was extracted with pentane (2 × 5 mL). The solution was filtered and the solvent was removed under vacuum to afford complex **2** as a deep blue powder. Yield of **2**: 48.1 mg (73%).

The synthesis of **^{13}C-2** was performed similarly to **2** from **^{13}C-1**. The yield and NMR data were matching those of **2**.

Crystals, suitable for X-ray analysis, were obtained by slow evaporation of a concentrated solution of **2** in pentane at rt. The crystals appeared deep brown.

^{1}H NMR (400 MHz, d_8-THF, –50 °C) δ 7.58 (s, 2H, C*H*, imidazole, H15), 7.39 (t, *J* = 7.7 Hz, 2H, CH_{arom}, Dipp, *para*, H10), 7.28 (dd, *J* = 11.6, 7.5 Hz, 4H, CH_{arom}, Dipp, *meta*, H8/9), 3.74 – 3.65 (m, 2H, Dipp, C*H*$(CH_3)_2$, H6/7), 2.78 – 2.66 (m, 2H, Dipp, C*H*$(CH_3)_2$, H6/7), 1.55 (s, 15H, Cp*, H1), 1.33 – 1.25 (m, 17H, with pentane, Dipp, CH$(CH_3)_2$, H2-5), 1.06 (dd, *J* = 18.9, 6.7 Hz, 12H, Dipp, CH$(CH_3)_2$, H2-5).

^{13}C NMR (101 MHz, d_8-THF, –50 °C) δ 167.69 (C16), 148.00 (C11/12), 146.54 (C11/12), 135.48 (C13), 130.40 (C10), 128.68 (C17, confirmed with ^{13}C-labelling), 125.55 (C8/9), 123.82 (C8/9), 122.66 (C15), 85.14 (C14), 29.94 (C6/7), 28.85 (C6/7), 26.63 (C2-5), 26.22 (C2-5), 23.78 (C2-5), 22.98 (C2-5), 10.07 (C1). $^{1}J(^{13}C–C16)$ = 62.1 Hz.

HRMS (nanochip-ESI/LTQ-Orbitrap) *m/z*: $[M - Cl]^+$ Calcd. for $C_{38}H_{51}ClIrN_2^+$ 763.3365; Found 763.3326.

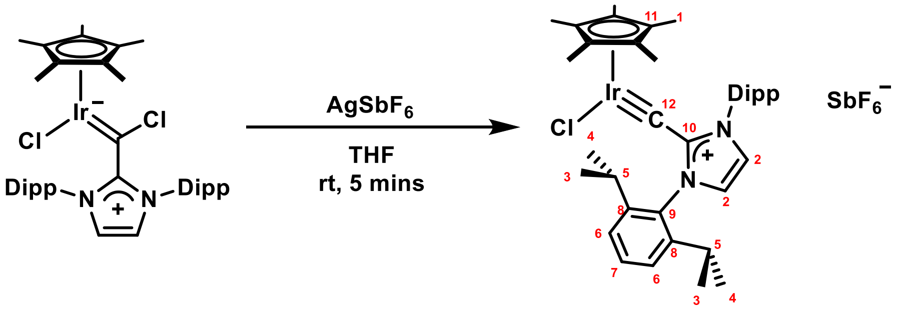

Complex 3: A solution of $AgSbF_6$ (21.5 mg, 63 μmol) in THF (0.5 mL) was added dropwise under stirring to a solution of complex **2** (50.0 mg, 63 μmol) in THF (1 mL). The mixture was stirred for 5 min. The solution was filtered and the solvent was removed under vacuum. The residue was washed with pentane (2 × 2 mL). The remaining solid was dried under vacuum to afford complex **3** as a red-purple powder. Yield of **3**: 57.2 mg (92%).

The synthesis of ^{13}**C-3** was performed similarly to **3** from ^{13}**C-2**. The yield and NMR data were matching those of **3**.

Due to the low intensity of C10 in ^{13}**C-3**, we were not able to resolve $^{1}J(^{13}C–C10)$.

Crystals, suitable for X-ray analysis, were obtained by layering pentane onto a concentrated solution of **3** in THF at –40 °C. The crystals appeared dark red.

^{1}H NMR (800 MHz, d_8-THF) δ 8.22 (s, 2H, C*H*, imidazole, H2), 7.62 (t, *J* = 7.8 Hz, 2H, C*H*$_{arom}$, Dipp, *para*, H7), 7.46 (d, *J* = 7.8 Hz, 4H, C*H*$_{arom}$, Dipp, *meta*, H6), 2.66 (hept, *J* = 6.9 Hz, 4H, Dipp, C*H*$(CH_3)_2$, H5), 1.90 (s, 15H, Cp*, H1), 1.38 (d, *J* = 6.9 Hz, 12H, Dipp, CH(C*H*$_3$)$_2$, H3/4), 1.26 (d, *J* = 6.8 Hz, 12H, Dipp, CH(C*H*$_3$)$_2$, H3/4).

^{13}C NMR (201 MHz, d_8-THF) δ 153.22 (C10), 146.29 (C8), 133.24 (C7), 131.40 (C9), 129.07 (C2), 126.01 (C6), 105.65 (C11), 30.49 (C5), 24.55 (C3/4), 24.03 (C3/4), 10.17 (C1). Note: C–C≡Ir was not observed, however ^{13}C-labelling studies suggested that C12 has a shift of 303.88 ppm.

HRMS (nanochip-ESI/LTQ-Orbitrap) *m/z*: $[M]^+$ Calcd. for $C_{38}H_{51}ClIrN_2^+$ 763.3365; Found 763.3347.

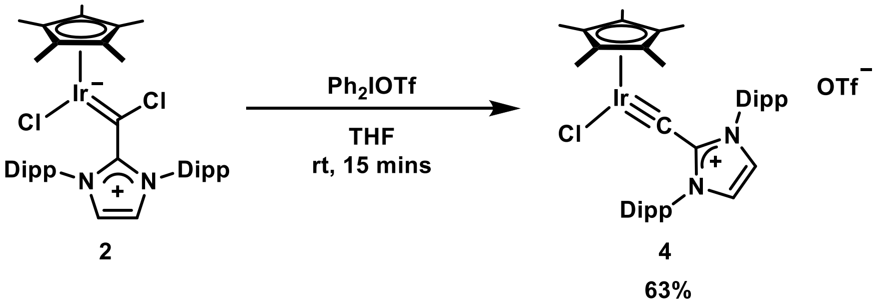

Complex 4: A solution of Ph_2IOTf (5.38 mg, 13 µmol) in THF (0.5 mL) was added dropwise under stirring to a solution of complex **2** (10.0 mg, 13 µmol) in THF (0.5 mL). The mixture was stirred for 15 min. The solution was filtered and the volatiles were removed under vacuum under a 40 °C bath for 5 h. The residue was washed with pentane (2 × 2 mL). The remaining solid was dried under vacuum to afford complex **4** as a red powder. Yield of **4**: 7.2 mg (63%).

Crystals, suitable for X-ray analysis, were obtained by layering pentane onto a concentrated solution of **4** in THF at –40 °C. The crystals appeared dark red.

The MS and NMR data were matching those of **3**.

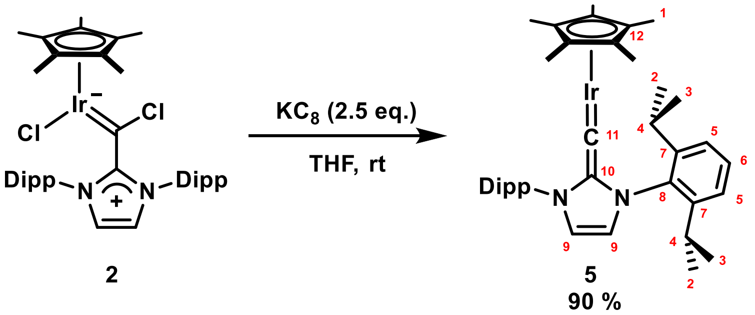

Complex 5: A mixture of KC_8 (21.1 mg, 156 µmol) in THF (0.5 mL) was added dropwise under stirring (stirring bars with borosilicate glass) to a solution of complex **2** (50.0 mg, 62 µmol) in THF (1 mL). The mixture was stirred for 3 h at rt. The solution was filtered and the solvent was removed under vacuum. The crude was extracted with pentane (3 × 5 mL). The solution was filtered and the solvent was removed under vacuum to afford complex **5** as an orange powder. Yield of **5**: 41.0 mg (90%).

The synthesis of **^{13}C-5** was performed similarly to **5** from **^{13}C-2**. The yield and NMR data were matching those of **5**.

Crystals, suitable for X-ray analysis, were obtained by slow evaporation of a concentrated solution of **5** in pentane at –40 °C. The crystals appeared orange to colorless.

^{1}H NMR (800 MHz, d_8-THF) δ 7.38 (t, *J* = 7.7 Hz, 2H, C*H*$_{arom}$, Dipp, *para*, H6), 7.21 (d, *J* = 7.7 Hz, 4H, C*H*$_{arom}$, Dipp, *meta*, H5), 7.00 (s, 2H, C*H*, imidazole, H9), 2.73 (hept, *J* = 6.9 Hz, 4H, Dipp, C*H*(CH_3)$_2$, H4), 1.86 (s, 15H, Cp*, H1), 1.41 (d, *J* = 6.9 Hz, 12H, Dipp, CH(C*H*$_3$)$_2$, H2/3), 1.17 (d, *J* = 7.0 Hz, 12H, Dipp, CH(C*H*$_3$)$_2$, H2/3).
^{13}C NMR (201 MHz, d_8-THF) δ 153.97 (C10), 147.19 (C7), 135.08 (C8), 130.20 (C6), 124.23 (C5), 119.83 (C9), 83.27 (C12), 30.08 (C4), 24.81 (C2/3), 24.23 (C2/3), 12.55 (C1). Note: the signal for C=<u>C</u>=Ir was not observed. However, ^{13}C-labelling studies suggested that C11 has a shift of 167.74 ppm. $^{1}J(^{13}$C–C11) = 58.9 Hz, $^{2}J(^{13}$C–C12) = 2.5 Hz.
HRMS (nanochip-ESI/LTQ-Orbitrap) *m*/*z*: [M + H]$^+$ Calcd. for $C_{38}H_{52}IrN_2^+$ 729.3754; Found 729.3784.

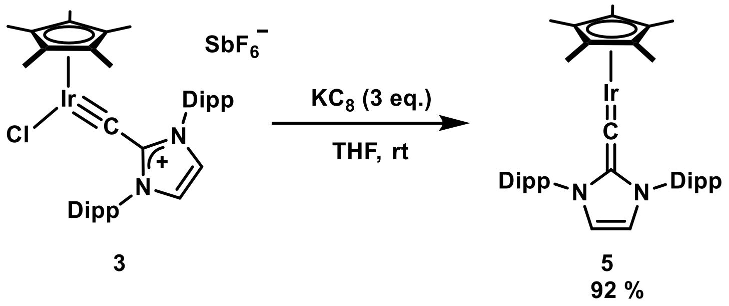

Complex 5: A mixture of KC_8 (8.1 mg, 60 µmol) in THF (0.5 mL) was added dropwise under stirring (stirring bars with borosilicate glass) to a solution of complex **3** (20.0 mg, 20 µmol) in THF (1 mL). The mixture was stirred for 2 h at rt. The solution was filtered and the solvent was removed under vacuum. The crude was extracted with pentane (3 × 5 mL). The solution was filtered and the solvent was removed under vacuum to afford complex **5** as an orange powder. Yield of **5**: 13.4 mg (92%)
The MS and NMR data were matching those of **5**.

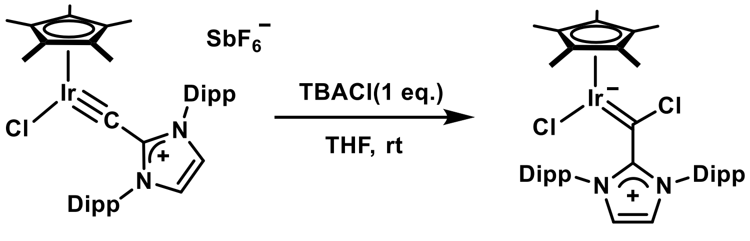

3 **2** **72%**

Complex 2: A solution of TBACl (2.78 mg, 10 µmol) in THF (0.5 mL) was added dropwise under stirring to a solution of complex **3** (10.0 mg, 10 µmol) in THF (1 mL). The mixture was stirred for 5 min. The solution was filtered and the solvent was removed under vacuum. The crude was extracted with pentane (2 × 5 mL). The solution was filtered and the solvent was removed under vacuum to afford complex **2** as a deep blue powder. Yield of **2**: 5.8 mg (72%)

The MS and NMR data were matching those of **2**.

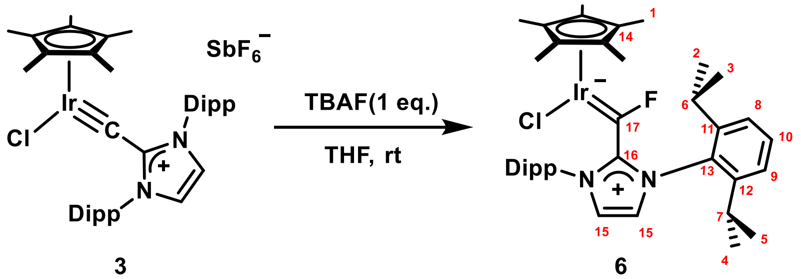

3 **6** **55 %**

Complex 6: A solution of TBAF (1M in THF, 20.0 µL, 20 µmol) was added dropwise under stirring to a solution of complex **3** (20.0 mg, 10 µmol) in THF (1 mL). The mixture was stirred for 15 min. The solution was filtered and the solvent was removed under vacuum. The crude was extracted with pentane (2 × 5 mL). The solution was filtered and the solvent was removed under vacuum to afford complex **6** as a deep purple powder. Yield of **6**: 8.6 mg (55%). The solid contains pentane and unknown impurities after drying as indicated by the $^{1}H/^{13}C\{^{1}H\}$-NMR spectra.

Crystals, suitable for X-ray analysis, were obtained by slow evaporation of a concentrated solution of **6** in pentane at rt. The crystals appeared dark brown.

^{1}H NMR (800 MHz, d_8-THF) δ 7.37 – 7.35 (m, 4H, C*H*, imidazole, H15 and CH_{arom}, Dipp, *para*, H10), 7.24 (d, *J* = 7.7 Hz, 4H, CH_{arom}, Dipp, *meta*, H8/9), 3.05 (hept, *J* =

6.8 Hz, 4H, Dipp, C*H*(CH_3)$_2$, H6/7), 1.65 (s, 15H, Cp*, H1), 1.35 (d, *J* = 6.7 Hz, 12H, Dipp, CH(C*H*$_3$)$_2$, H2-5), 1.07 (d, *J* = 6.9 Hz, 12H, Dipp, CH(C*H*$_3$)$_2$, H2-5).

^{1}H NMR (400 MHz, *d*$_8$-THF, –50 °C) δ 7.63 (s, 2H, C*H*, imidazole, H15), 7.41 (t, *J* = 7.8 Hz, 2H, C*H*$_{arom}$, Dipp, *para*, H10), 7.29 (d, *J* = 7.7 Hz, 4H, C*H*$_{arom}$, Dipp, *meta*, H8/9), 1.65 (s, 15H, Cp*, H1), 1.33 (d, *J* = 6.6 Hz, 14H, with grease and pentane, Dipp, CH(C*H*$_3$)$_2$, H2-5), 1.07 (d, *J* = 6.9 Hz, 12H, Dipp, CH(C*H*$_3$)$_2$, H2-5). Note: H6/7 was not observed.

^{13}C NMR (201 MHz, *d*$_8$-THF) δ 147.39 (C11/12), 134.72 (C13), 130.41 (C10), 124.57 (C8/9), 122.63 (C15), 84.97 (C14), 29.49 (C6/7), 23.93 (C2-5) 10.57 (C1). The signals of C2-5 overlapped with the signal of *d*$_8$-THF.

^{13}C NMR (101 MHz, *d*$_8$-THF, –50 °C) δ 164.73 (C16), 146.98 (C11/12), 134.37 (C13), 130.47(C10), 124.64 (C8/9), 122.88 (C15), 84.60 (C14), 29.44 (C6/7), 23.82 (C2-5), 10.75 (C1). Note: the signal for C17 was not observed. The signals of C2-5 overlapped with the signal of *d*$_8$-THF.

^{19}F NMR (376 MHz, *d*$_8$-THF, –50 °C) δ –44.52.

HRMS (nanochip-ESI/LTQ-Orbitrap) *m/z*: [M + H]$^+$ Calcd. for $C_{38}H_{52}ClFIrN_2^+$ 783.3427; Found 783.3455.

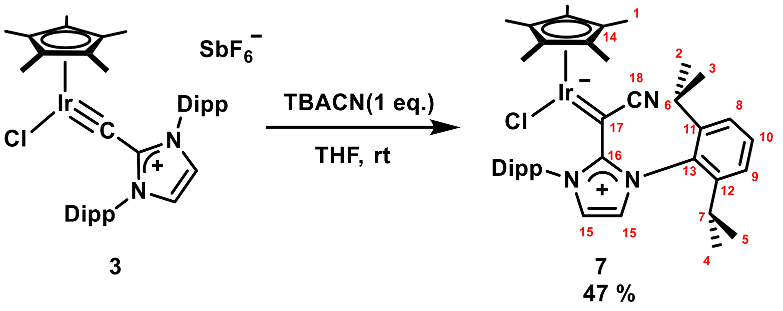

Complex 7: A solution of TBACN (5.4 mg, 20 μmol) in THF (0.5 mL) was added dropwise under stirring to a solution of complex **3** (20.0 mg, 20 μmol) in THF (1 mL). The mixture was stirred for 1 h at rt. The solution was filtered and the solvent was removed under vacuum. The crude was extracted with pentane (3 × 5 mL). The solution was filtered and the solvent was removed under vacuum to afford complex **7** as a deep blue powder. Yield of **7**: 9.3 mg (47%). The solid contains grease after drying as indicated by the ^{1}H/^{13}C{^{1}H}-NMR spectra.

Crystals, suitable for X-ray analysis, were obtained by slow evaporation of a concentrated solution of **7** in pentane at –40 °C. The crystals appeared black.

^{1}H NMR (600 MHz, d_8-THF) δ 7.33 (t, J = 7.6 Hz, 2H, CH_{arom}, Dipp, *para*, H10), 7.30 (s, 2H, CH, imidazole, H15), 7.23 (d, 4H, CH_{arom}, Dipp, *meta*, H8/9), 3.51 (br m, 2H, Dipp, CH(CH$_3$)$_2$, H6/7), 2.66 (br m, 2H, Dipp, CH(CH$_3$)$_2$, H6/7), 1.44-1.26 (m, 21H, Cp*, H1 and Dipp, CH(CH_3)$_2$, H2-5), 1.30-1.23 (m, 7H, with grease, Dipp, CH(CH_3)$_2$, H2-5), 1.10-1.01 (m, 12H, Dipp, CH(CH_3)$_2$, H2-5).

^{1}H NMR (400 MHz, d_8-THF, –50 °C) δ 7.57 (s, 2H, CH, imidazole, H15), 7.37 (t, J = 7.6 Hz, 2H, CH_{arom}, Dipp, *para*, H10), 7.31 – 7.24 (m, 4H, CH_{arom}, Dipp, *meta*, H8/9), 3.50 (m, 2H, Dipp, CH(CH$_3$)$_2$, H6/7), 2.64 – 2.53 (m, 2H, Dipp, CH(CH$_3$)$_2$, H6/7), 1.44 (s, 15H, Cp*, H1), 1.40 (d, J = 6.6 Hz, 6H, Dipp, CH(CH_3)$_2$, H2-5), 1.24 (d, J = 6.4 Hz, 9H, with grease, Dipp, CH(CH_3)$_2$, H2-5), 1.06 (dd, J = 22.8, 6.7 Hz, 13H, with grease, Dipp, CH(CH_3)$_2$, H2-5).

^{13}C NMR (151 MHz, d_8-THF) δ 170.22 (C16), 148.31 (C11/12), 147.06 (C11/12), 135.05 (C13), 131.88 (C17), 130.44 (C10), 125.39 (C8/9), 124.27 (C8/9), 122.30 (C15), 96.05 (C18), 85.51 (C14), 30.12 (C6/7), 28.97 (C6/7), 22.83 (C2-5), 8.89 (C1). Note: The signals of C2-5 overlapped with the signal of d_8-THF.

^{13}C NMR (101 MHz, d_8-THF, –50 °C) δ 169.63 (C16), 147.85 (C11/12), 146.74 (C11/12), 134.80 (C13), 130.48 (C10), 125.50 (C8/9), 124.20 (C8/9), 122.60 (C15), 85.20 (C14), 30.13 (C6/7), 28.86 (C6/7), 26.46 (C2-5), 26.38 (C2-5), 22.71 (C2-5), 8.91 (C1). Note: The signals of C2-5 overlapped with the signal of d_8-THF. The signals of N<u>C</u>-<u>C</u>=Ir (C17 and C18) were not observed.

HRMS (nanochip-ESI/LTQ-Orbitrap) *m*/*z*: [M + H]$^+$ Calcd. for $C_{39}H_{52}ClIrN_3^+$ 790.3473; Found 790.3507.

FT-IR: $\tilde{\nu}$/cm^{-1} = 2115 ν(C≡N).

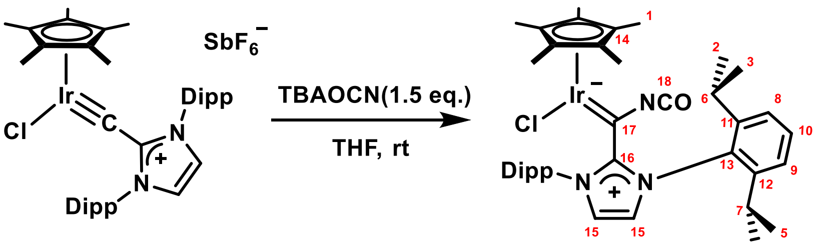

3

8
84 %

Complex 8: A solution of TBAOCN (8.5 mg, 30 μmol) in THF (0.5 mL) was added dropwise under stirring to a solution of complex **3** (20.0 mg, 20 μmol) in THF (1 mL). The mixture was stirred for 1 h at rt. The solution was filtered and the solvent was

removed under vacuum. The crude was extracted with pentane (3 × 5 mL). The solution was filtered, and the solvent was removed under vacuum to afford complex **8** as a deep blue powder. Yield of **8**: 13.5 mg (84%). The solid contained pentane and grease after drying, as indicated by the $^{1}H/^{13}C\{^{1}H\}$-NMR spectra.
Crystals, suitable for X-ray analysis, were obtained by slow evaporation of a concentrated solution of **8** in pentane at –40 °C. The crystals appeared dark green.

^{1}H NMR (800 MHz, d_8-THF) δ 7.36 (t, *J* = 7.7 Hz, 2H, CH_{arom}, Dipp, *para*, H10), 7.33 (s, 2H, C*H*, imidazole, H15), 7.27 (dd, *J* = 23.1, 7.8 Hz, 4H, CH_{arom}, Dipp, *meta*, H8/9), 3.64 – 3.60 (m, 2H, Dipp, C*H*(CH_3)$_2$, H6/7), 2.62 (m, 2H, Dipp, C*H*(CH_3)$_2$, H6/7), 1.57 (s, 15H, Cp*, H1), 1.35 (m, 12H, Dipp, CH(CH_3)$_2$, H2-5), 1.09 – 1.03 (m, 12H, Dipp, CH(CH_3)$_2$, H2-5).
^{1}H NMR (400 MHz, d_8-THF, –50 °C) δ 7.58 (s, 2H, C*H*, imidazole, H15), 7.41 (t, *J* = 7.6 Hz, 2H, CH_{arom}, Dipp, *para*, H10), 7.32 (t, *J* = 7.3 Hz, 4H, CH_{arom}, Dipp, *meta*, H8/9), 2.60 – 2.49 (m, 2H, Dipp, C*H*(CH_3)$_2$, H6/7), 1.57 (s, 15H, Cp*, H1), 1.34 (t, *J* = 7.2 Hz, 14H, with grease, Dipp, CH(CH_3)$_2$, H2-5), 1.07 (dd, *J* = 21.9, 6.7 Hz, 14H, with grease, Dipp, CH(CH_3)$_2$, H2-5). Note: one pair of signals of H6/7 overlapped with the signal of d_8-THF.
^{13}C NMR (151 MHz, d_8-THF) δ 168.57 (C16), 148.24 (C11/12), 146.46 (C11/12),, 135.14 (C13), 130.48 (C10), 125.69 (C8/9), 124.25 (C8/9), 124.05 (C17/18), 123.48 (C17/18), 122.25 (C15), 84.70 (C14), 30.07 (C6/7), 29.01 (CC6/7), 23.38 (C2-5), 22.76 (C2-5), 10.37 (C1). Note: The signals of C2-5 overlapped with the signal of d_8-THF.
^{13}C NMR (101 MHz, d_8-THF, –50 °C) δ 167.83 (C16), 147.78 (C11/12), 146.26 (C11/12), 134.81 (C13), 130.55 (C10), 125.78 (C8/9), 124.03 (C8/9), 122.53 (C15), 84.33 (C14), 30.13 (C6/7), 28.90 (C6/7), 26.53 (C2-5), 26.29 (C2-5), 22.64 (C2-5), 10.52 (C1). Note: C17 and C18 were not observed. The signals of C2-5 overlapped with the signal of d_8-THF.
HRMS (nanochip-ESI/LTQ-Orbitrap) *m/z*: $[M]^+$ Calcd. for $C_{39}H_{51}ClIrN_3O^+$ 805.3344; Found 805.3373.

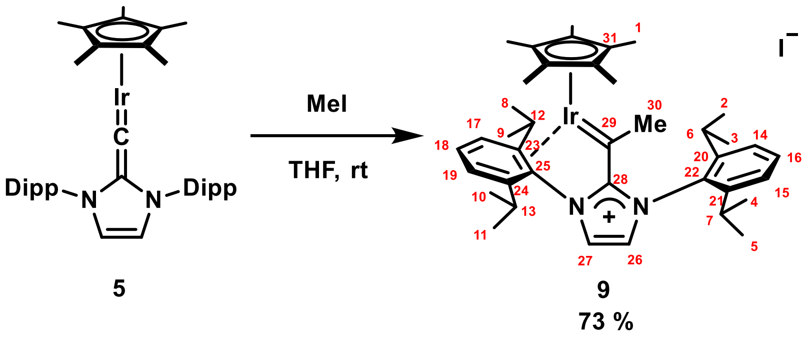

Complex 9: MeI (1.3 µL, 21 µmol) was added under stirring to a solution of complex **5** (15 mg, 21 µmol) in THF. An immediate colour change from deep red to dark purple was observed. The mixture was stirred for 10 min at rt. The mixture was filtered and the solid was washed with pentane (3 × 5 mL). The solid was dried under vacuum to afford complex **9** as a deep purple solid. Yield of **9**: 13.1 mg (73%).
Crystals, suitable for X-ray analysis, were obtained by layering pentane onto a concentrated solution of **9** in THF at –40 °C. The crystals appeared dark to deep red.

^{1}H NMR (600 MHz, d_8-THF) δ 8.76 (d, J = 2.0 Hz, 1H, CH, imidazole, H26/27), 8.44 (d, J = 2.1 Hz, 1H, CH, imidazole, H26/27), 7.57 (t, J = 7.8 Hz, 1H, CH_{arom}, Dipp, *para*, H16/18), 7.38 (d, J = 7.8 Hz, 2H, CH_{arom}, Dipp, *meta*, H14/15/17/19), 7.00 (d, J = 6.9 Hz, 2H, CH_{arom}, Dipp, *meta*, H14/15/17/19), 6.95 (dd, J = 8.7, 6.6 Hz, 1H, CH_{arom}, Dipp, *para*, H16/18), 2.60 (hept, J = 7.1 Hz, 2H, Dipp, CH(CH_3)$_2$, H6/7/12/13), 2.04 (hept, J = 6.7 Hz, 2H, Dipp, CH(CH_3)$_2$, H6/7/12/13), 1.58 (s, 15H, Cp*, H1), 1.24 (d, J = 6.9 Hz, 6H, Dipp, CH(CH_3)$_2$, H2-5/8-11), 1.17 (dd, J = 6.9, 3.4 Hz, 12H, Dipp, CH(CH_3)$_2$, H2-5/8-11), 1.05 (d, J = 6.7 Hz, 6H, Dipp, CH(CH_3)$_2$, H2-5/8-11), 0.40 (s, 3H, Me, H30).
Due to limited stability of the complex in solution, a full characterization via ^{13}C NMR was not achieved.
HRMS (nanochip-ESI/LTQ-Orbitrap) *m/z*: [M]$^+$ Calcd. for $C_{39}H_{54}IrN_2^+$ 743.3911; Found 743.3931.

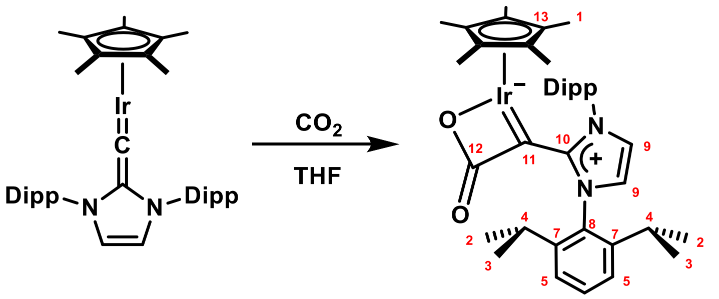

5 **10** **82 %**

Complex 10: A solution of complex **5** (10 mg, 14 μmol) in THF (0.5 mL) was added to a J-Young NMR tube. The solution was degassed by three freeze-pump-thaw cycles and then placed under an atmosphere of CO_2 and inverted several times. The resulting mixture was allowed to stand. Over the course of 24 h, the solution turned dark. The solution was decanted into a 10 mL vial and the volatiles were removed under vacuum to afford complex **10** as dark-brown solid. Yield of **10**: 8.7 mg (82%).

Crystals, suitable for X-ray analysis, were obtained by dissolving complex **5** in degassed pentane in a J-Young NMR tube under CO_2 and letting the mixture stand for 48 h. The crystals appeared dark brown.

^{1}H NMR (800 MHz, d_8-THF) δ 7.34 (t, J = 7.8 Hz, 2H, CH_{arom}, Dipp, *para*, H6), 7.28 – 7.25 (m, 4H, CH_{arom}, Dipp, *meta*, H5), 7.12 (d, J = 1.6 Hz, 2H, C*H*, imidazole, H9), 3.10 (hept, J = 6.8 Hz, 4H, Dipp, C*H*(CH_3)$_2$, H4), 1.50 (s, 15H, Cp*, H1), 1.41 (dd, J = 6.8, 1.8 Hz, 12H, Dipp, CH(CH_3)$_2$, H2/3), 1.09 (dd, J = 7.2, 1.8 Hz, 12H, Dipp, CH(CH_3)$_2$, H2/3).

^{13}C NMR (201 MHz, d_8-THF) δ 183.48 (C12), 159.70 (C10), 146.80 (C7), 136.06 (C8), 130.10 (C6), 125.30 (C5), 122.42 (C9), 115.84 (C11), 82.60 (C13), 29.98 (C4), 23.73 (C2/3),11.03 (C1). Note: The signals of C2/3 overlapped with the signal of d_8-THF.

HRMS (nanochip-ESI/LTQ-Orbitrap) *m*/*z*: $[M + H]^+$ Calcd. for $C_{39}H_{52}IrN_2O_2^+$ 773.3653; Found 773.3676.

FT-IR: $\tilde{\nu}$/cm^{-1} = 1621 v(C=O).

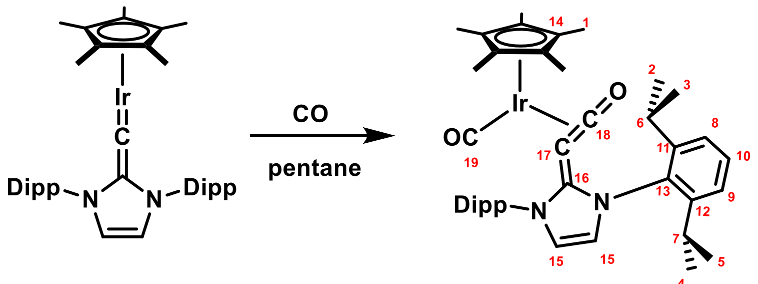

5 **11** **90%**

Complex 11: A solution of complex **5** (10 mg, 14 μmol) in pentane (0.5 mL) was added to a J-Young NMR tube. The solution was degassed by three freeze-pump-thaw cycles and then placed under an atmosphere of CO and inverted several times. A spontaneous colour change from red to yellow was observed. The resulting mixture was allowed to stand for 4 h. The solution was decanted into a 10 mL vial and the volatiles were removed under vacuum to afford complex **11** as a yellow oil. Yield of **11**: 9.7 mg (90%). The solid contains minor amounts of Cp*Ir(CO)$_2$ after work-up and drying as indicated by the ^{1}H/^{13}C{^{1}H} NMR and IR spectra.

The synthesis of **^{13}C-11** was performed similarly to **11** from **5** and 4 eq. ^{13}CO. The yield and NMR data were matching those of **11**.

Crystals, suitable for X-ray analysis, were obtained by slow evaporation of a concentrated solution of **11** in pentane at rt. The crystals appeared yellow.

^{1}H NMR (600 MHz, *d*$_8$-THF) δ 7.40 (t, *J* = 7.7 Hz, 2H, C*H*$_{arom}$, Dipp, *para*, H6), 7.34 – 7.27 (m, 2H, C*H*$_{arom}$, Dipp, *meta*, H8/9), 7.23 (m, 2H, C*H*$_{arom}$, Dipp, *meta*, H8/9), 6.76 (s, 2H, C*H*, imidazole, H9), 3.31 – 3.16 (m, 2H, Dipp, C*H*(CH$_3$)$_2$, H6/7), 2.71 (m, 2H, Dipp, C*H*(CH$_3$)$_2$, H6/7), 1.55 (s, 15H, Cp*, H1), 1.39 (m, 6H, Dipp, CH(C*H*$_3$)$_2$, H2-5), 1.24 (m, 8H, with grease, Dipp, CH(C*H*$_3$)$_2$, H2-5), 1.17 (d, *J* = 6.9 Hz, 12H, Dipp, CH(C*H*$_3$)$_2$, H2-5).

^{13}C NMR (151 MHz, *d*$_8$-THF) δ 178.26 (C19, confirmed with ^{13}C-labelling), 155.50 (C18, confirmed with ^{13}C-labelling), 148.77 (C11/12), 147.75 (C11/12), 143.09 (C16), 135.43 (C13), 130.61 (C15), 125.05 (C8/9), 124.58 (C8/9), 120.61 (C10), 97.67 (C14), 30.17 (C6/7), 29.58 (C6/7), 24.23 (C2-5), 23.14 (C2-5), 9.99 (C1). Note: The signals of C2-5 overlapped with the signal of *d*$_8$-THF.

HRMS (nanochip-ESI/LTQ-Orbitrap) *m/z*: [M]$^+$ Calcd. for $C_{40}H_{51}IrN_2O_2^+$ 784.3574; Found 784.3594.

FT-IR: $\tilde{\nu}$/cm^{-1} = 1917 *v*(Ir-CO), 1828 *v*(C=O).

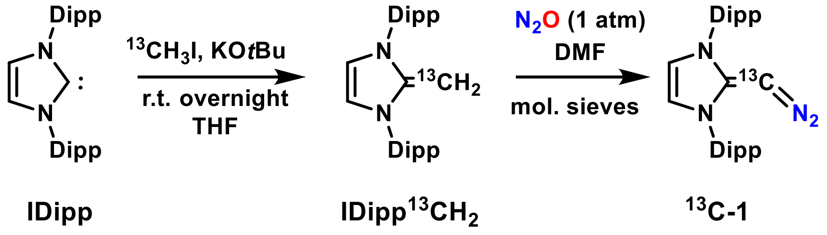

The synthesis was following a modified literature procedure reported by Varava *et al.*[1]

IDipp^{13}CH$_2$: KO*t*Bu (0.29 g, 2.60 mmol, 1.01 eq.) was added dropwise over 10 min to a solution of IDipp (1.00 g, 2.57 mmol, 1 eq.) in THF (20 mL). $^{13}CH_3I$ (0.16 mL, 2.62 mmol, 1.02 eq.) was added. The resulting mixture was stirred overnight at RT. Subsequently, the solvent was removed under reduced pressure. The resulting mixture was extracted with diethyl ether (3 × 20 mL). After removal of the solvent, the crude mixture was crystallized from pentane at –40°C overnight to afford white crystals. Yield of IDipp$^{13}CH_2$: 0.88 g (85%). The spectral data are in agreement with those for the non-labelled compound as described previously.[1]

^{1}H NMR (400 MHz, C_6D_6) δ 7.23 (dd, *J* = 8.6, 6.6 Hz, 2H, C*H*$_{arom}$, Dipp, *para*), 7.14 (q, *J* = 1.3 Hz, 4H, C*H*$_{arom}$, Dipp, *meta*), 5.85 (s, 2H, C*H*, imidazole), 3.35 (hept, *J* = 6.9 Hz, 4H, Dipp, C*H*(CH$_3$)$_2$), 1.36 (d, *J* = 6.9 Hz, 12H, Dipp, CH(C*H*$_3$)$_2$), 1.22 (d, *J* = 7.0 Hz, 12H, Dipp, CH(C*H*$_3$)$_2$).

^{13}C NMR (101 MHz, C_6D_6) δ 152.96 (N-*C*-N), 152.09 (N-*C*-N), 149.01 (Ar*C*), 134.98 (Ar*C*), 129.34 (Ar*C*), 124.60 (Ar*C*), 114.71 (-N-CH-), 56.22 (d, *J* = 49.5 Hz, =^{13}*C*H$_2$), 28.79 (*C*H(CH$_3$)$_2$), 24.37 (CH(*C*H$_3$)$_2$), 23.91(CH(*C*H$_3$)$_2$)

^{13}C-1: IDipp^{13}CH$_2$ (350 mg, 0.20 mmol) was dissolved in DMF (50 mL), and molecular sieves (4 Å, 1 g) were added to the flask. After three freeze-pump-thaw cycles, N_2O (~200 mL, 1 bar) was added at 0 °C. The resulting mixture was stirred for 2 days at RT, followed by removal of the sieves by filtration, and washing with DMF (10 mL). All solutions were combined and the solvent was removed under reduced pressure at 40 °C. The solid was crushed with a spatula. Pentane (10 mL) was added to the flask and the mixture was sonicated for 3 min. The solid was filtered out and washed with pentane (3 × 10 mL) and cold diethyl ether (1 mL), followed by extraction with toluene (50 mL). The solvent was removed under reduced pressure. The residue was washed

with pentane (3 × 5 mL) and dried under vacuum to afford a yellow powder. Yield of **^{13}C-1**: 315 mg (84%). The spectral data were in agreement with those of the non-labelled compound as described previously.[1]

^{1}H NMR (400 MHz, C_6D_6) δ 7.25 (t, *J* = 7.7 Hz, 2H, C*H*$_{arom}$, Dipp, *para*), 7.11 (d, *J* = 7.7 Hz, 4H, C*H*$_{arom}$, Dipp, *meta*), 5.92 (s, 2H, C*H*, imidazole), 2.99 (hept, *J* = 6.9 Hz, 4H, Dipp, C*H*(CH_3)$_2$), 1.42 (d, *J* = 6.8 Hz, 12H, Dipp, CH(CH_3)$_2$), 1.15 (d, *J* = 6.9 Hz, 12H, Dipp, CH(CH_3)$_2$).
^{13}C NMR (101 MHz, C_6D_6) δ 152.09 (d, *J* = 66.85 Hz, C, imidazole), 147.37 (C$_{arom}$, Dipp, *ortho*), 133.18 (C$_{arom}$, Dipp, *ipso*), 130.56 (HC$_{arom}$, Dipp, *para*), 124.18 (HC$_{arom}$, Dipp, *para*), 117.91 (HC, imidazole), 35.22 (=^{13}CN$_2$), 29.22 (*C*H(CH_3)$_2$), 24.05 (CH(*C*H$_3$)$_2$), 23.84 (CH(*C*H$_3$)$_2$).

3. NMR spectra

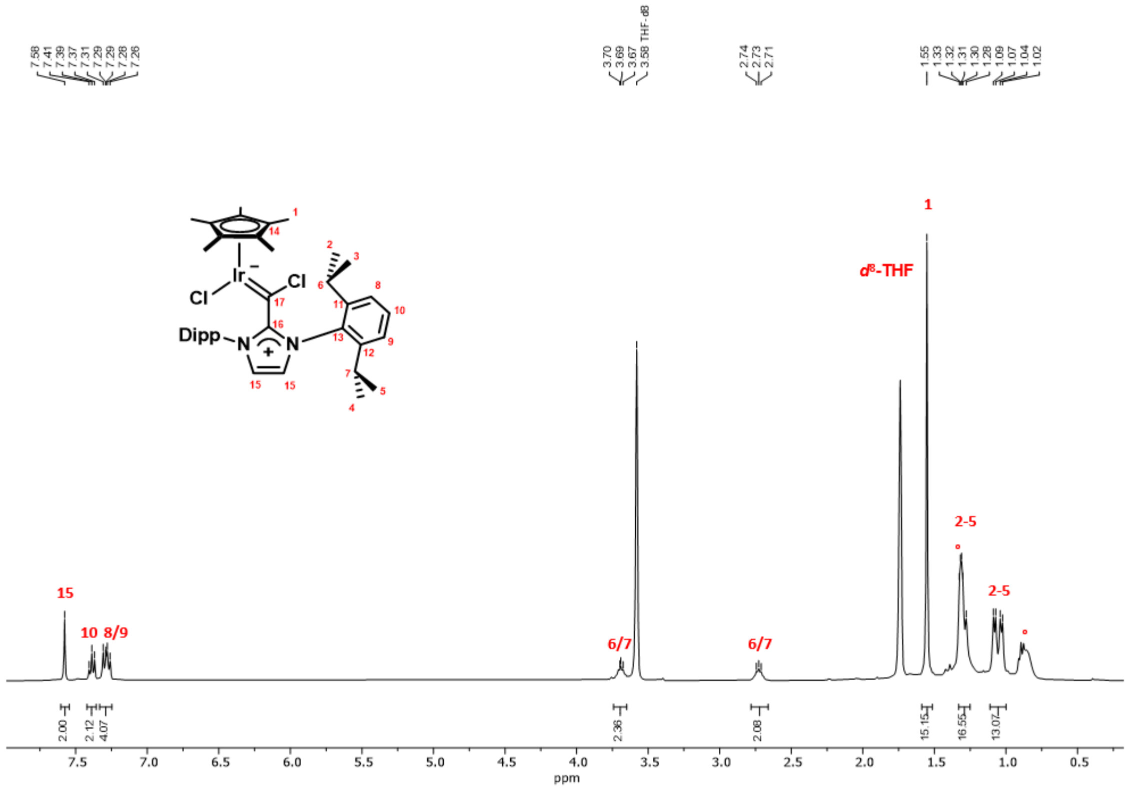

Figure S1 ^{1}H-NMR spectrum (400 MHz, d_8-THF, –50 °C) of complex **2**. °: pentane.

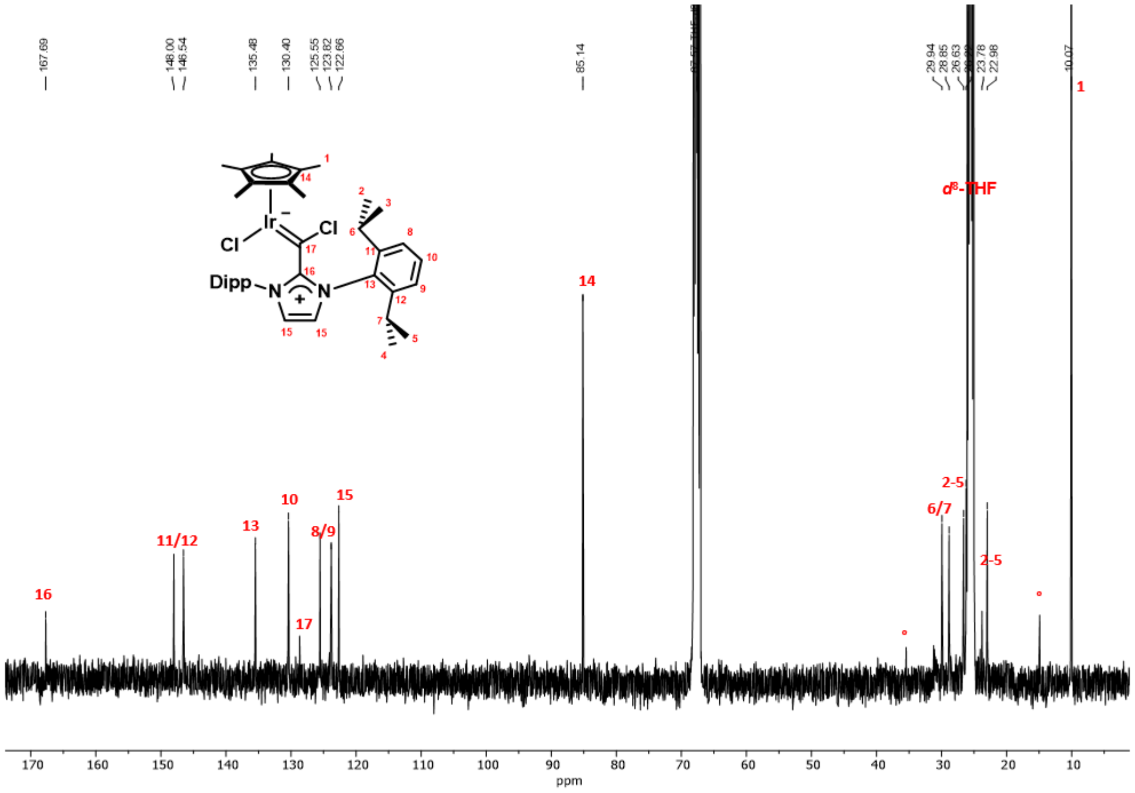

Figure S2 ^{13}C{^{1}H}-NMR spectrum (101 MHz, d_8-THF, –50 °C) of complex **2**. °: pentane.

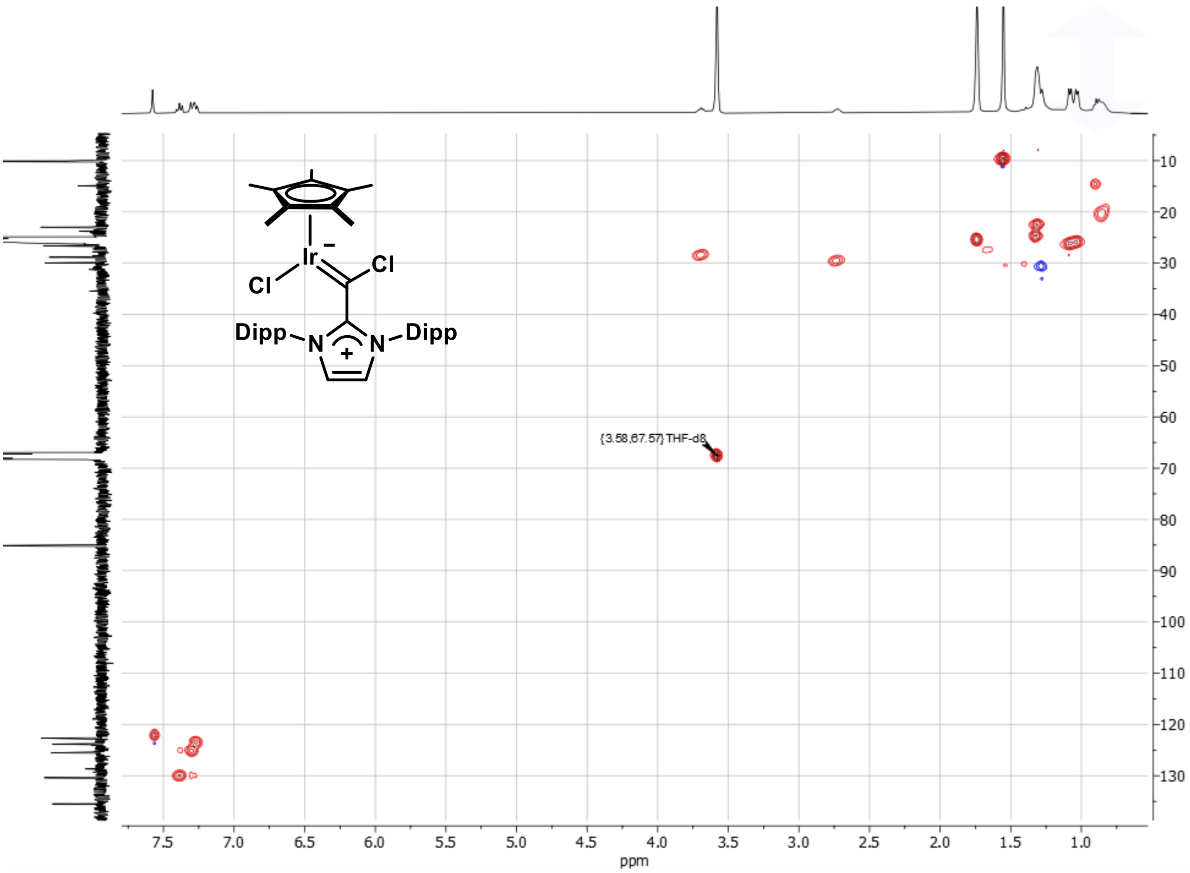

Figure S3 HSQC spectrum (101 MHz, d_8-THF, –50 °C) of complex **2**.

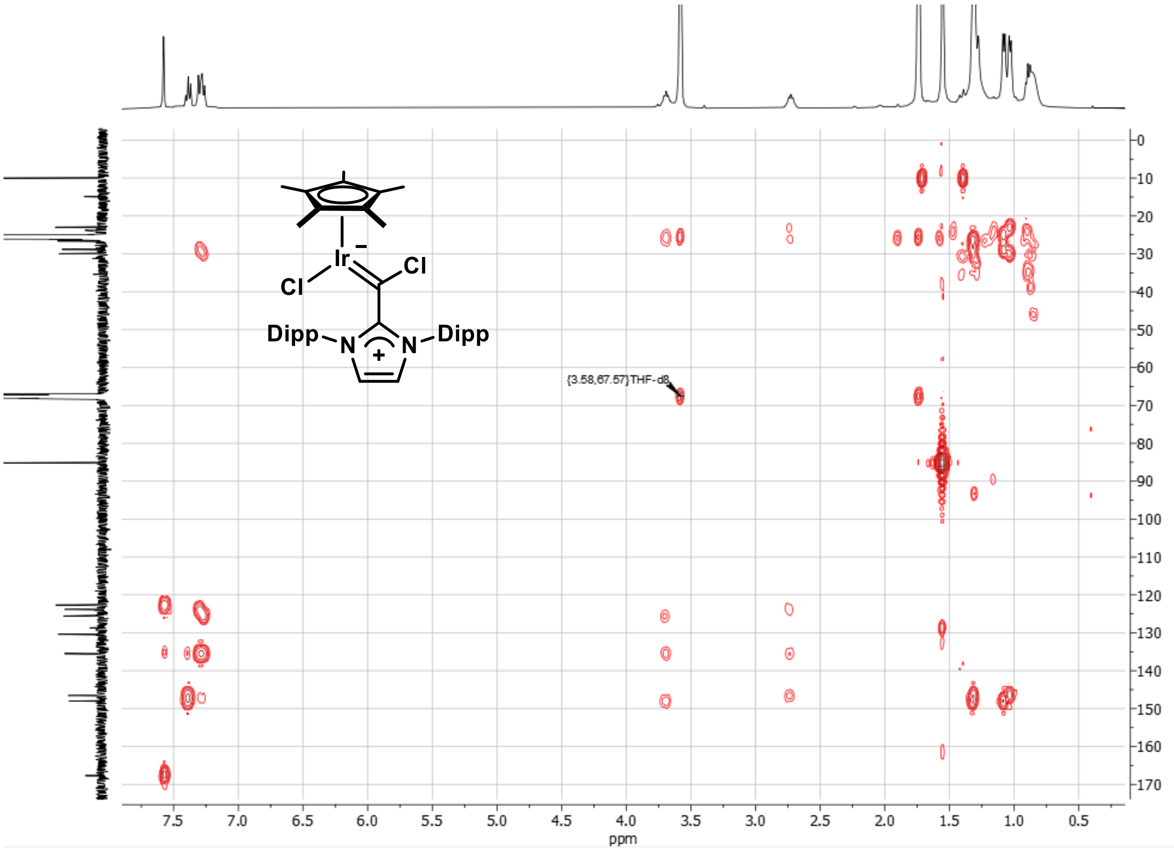

Figure S4 HMBC spectrum (101 MHz, d_8-THF, –50 °C) of complex **2**.

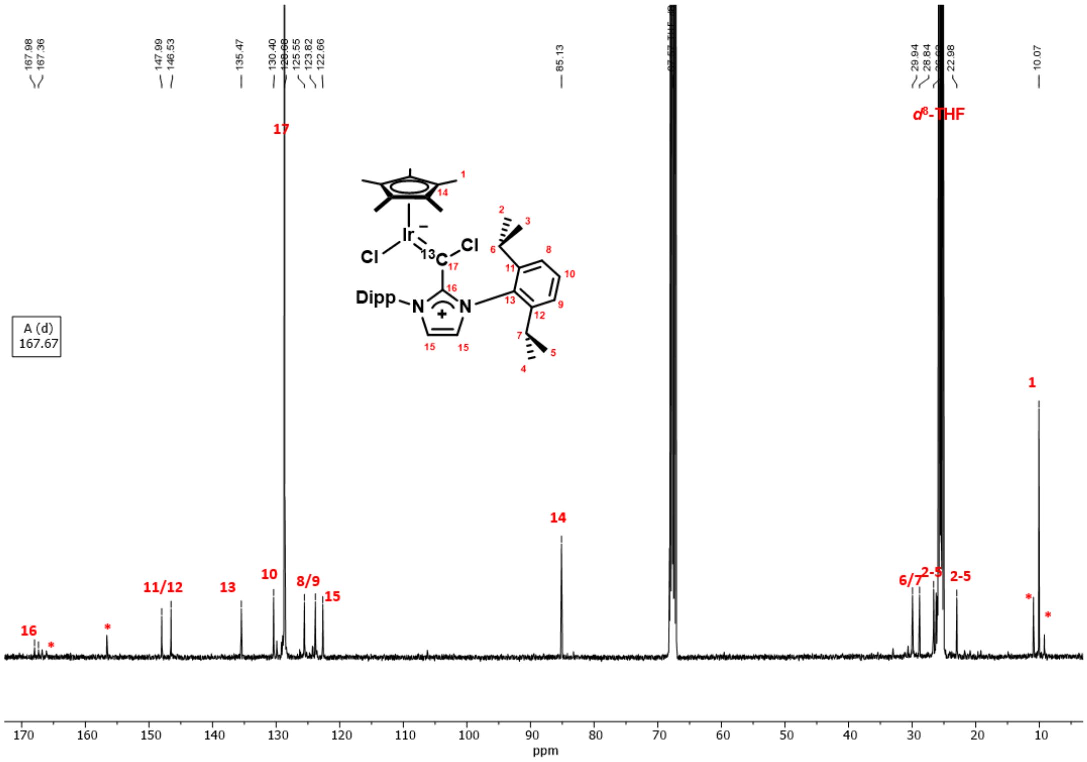

Figure S5 $^{13}C\{^{1}H\}$-NMR spectrum (101 MHz, d_8-THF, –50 °C) of complex **^{13}C-2**. The signal at 128.70 ppm indicated C–<u>C</u>=Ir. $^{1}J(^{13}C–C16)$ = 62.1 Hz. *: unknown impurities.

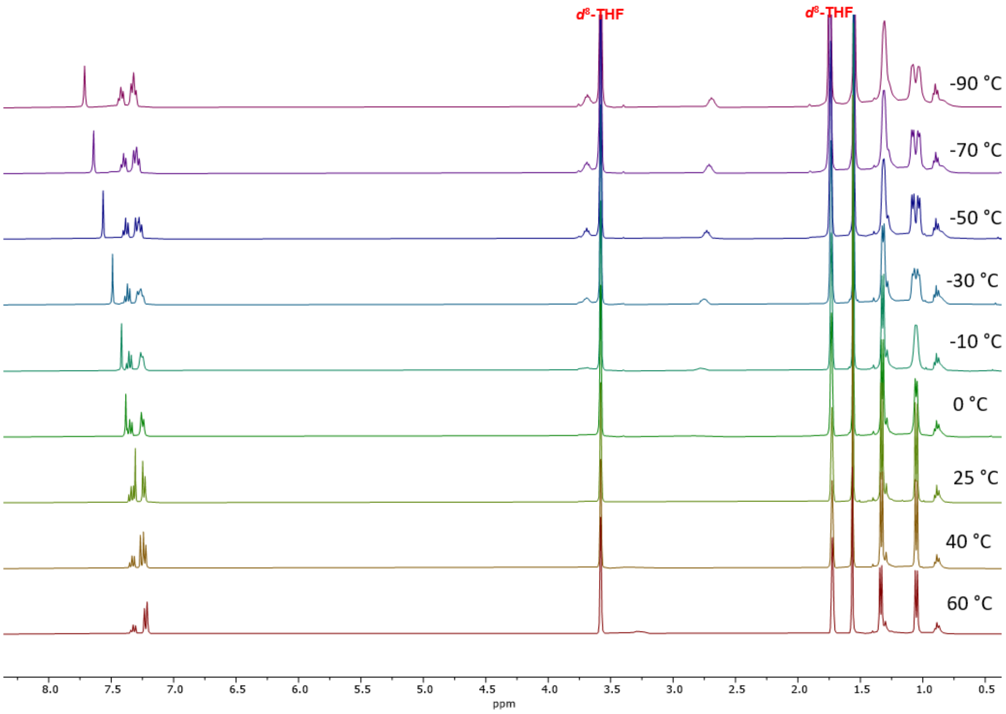

Figure S6 ^{1}H NMR spectra (400 MHz, d_8-THF) of complex **2** at different temperatures.

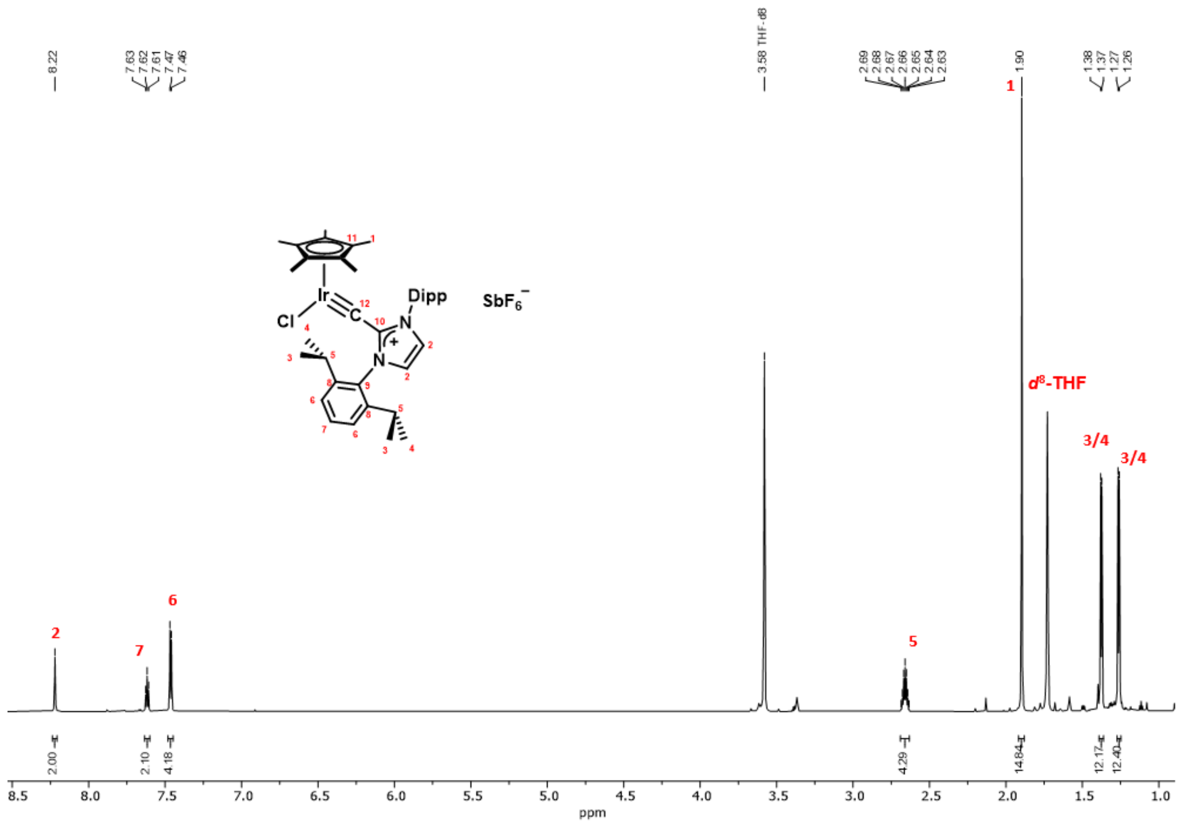

Figure S7 ^{1}H-NMR spectrum (800 MHz, d_8-THF) of complex **3**.

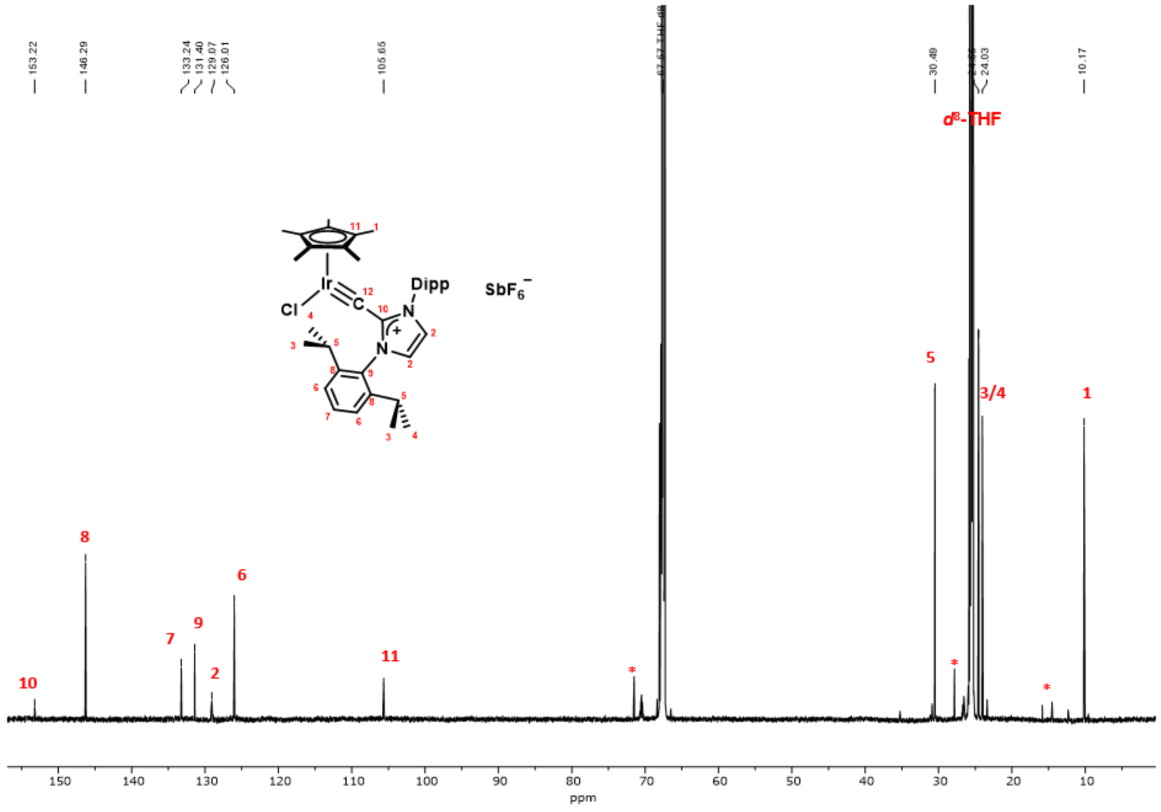

Figure S8 ^{13}C{^{1}H}-NMR spectrum (201 MHz, d_8-THF) of complex **3**. *: unknown impurities.

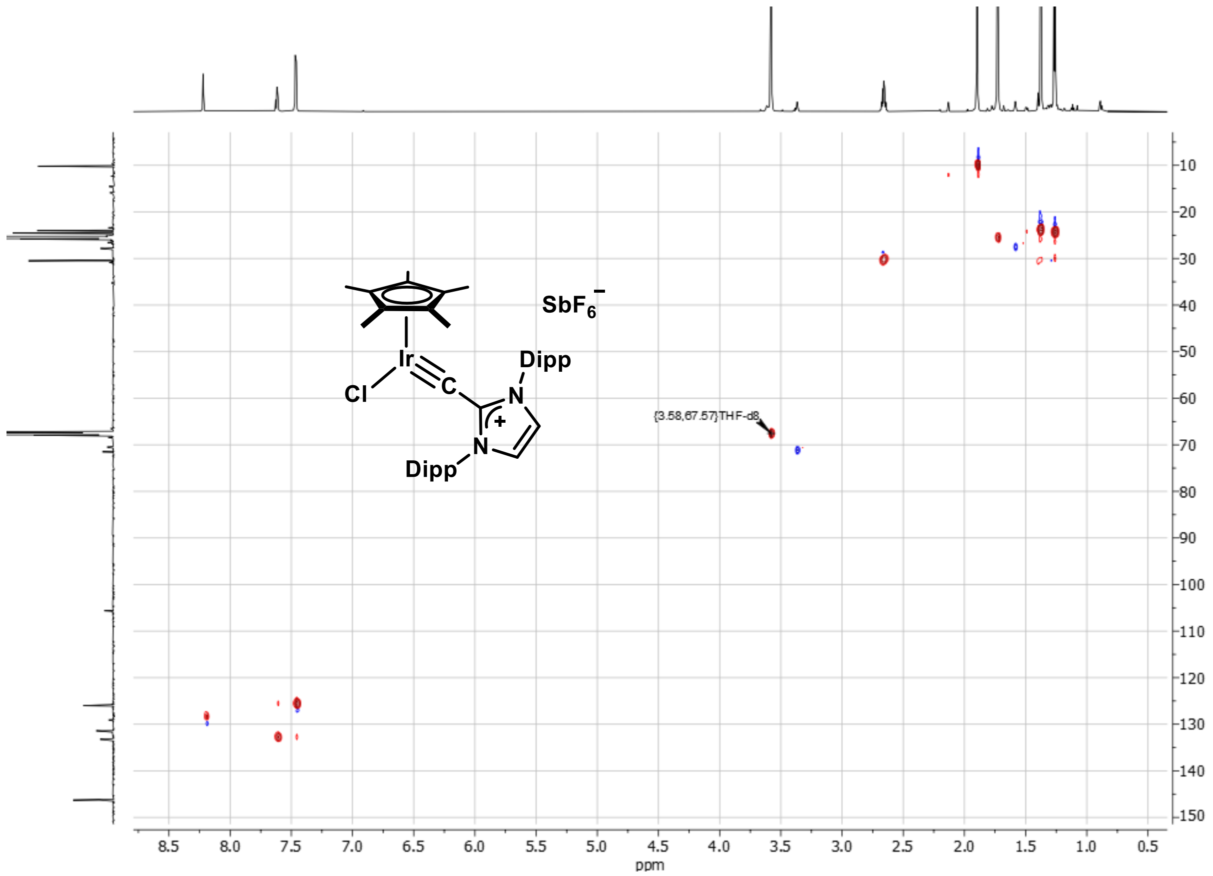

Figure S9 HSQC spectrum (201 MHz, d_8-THF) of complex **3**.

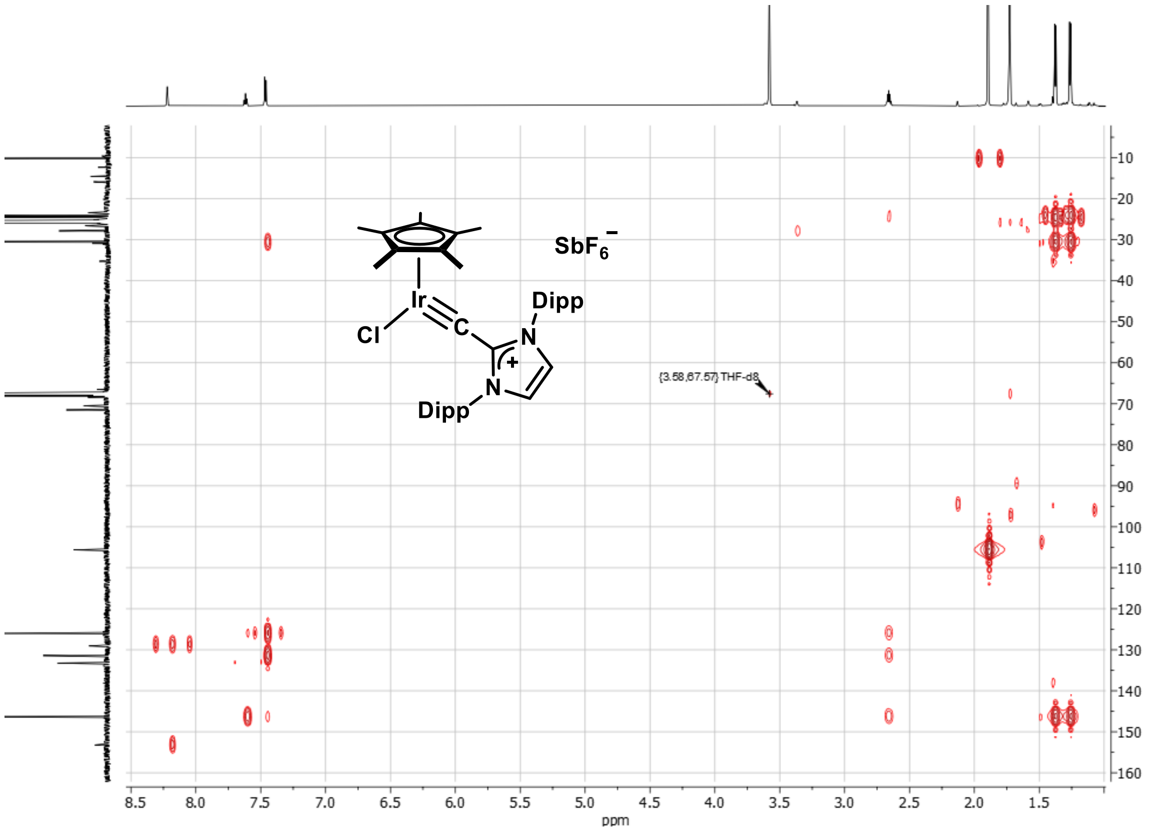

Figure S10 HMBC spectrum (201 MHz, d_8-THF) of complex **3**.

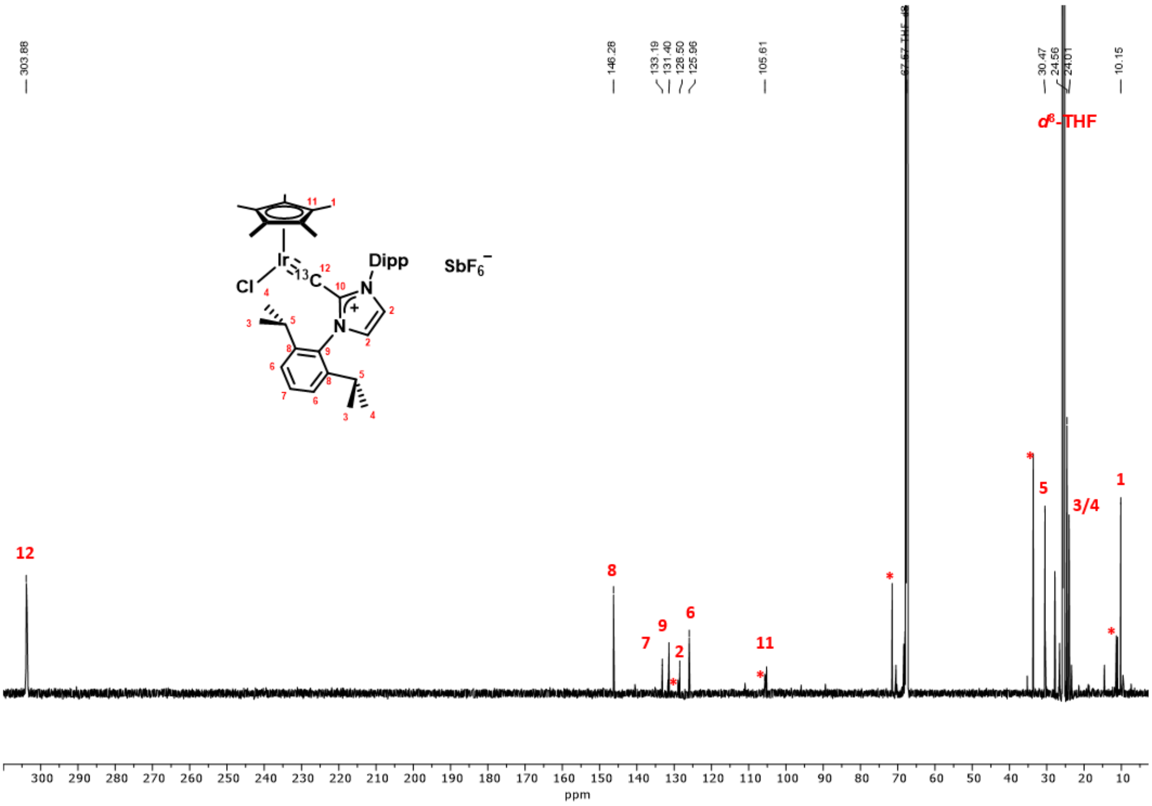

Figure S11 $^{13}C\{^{1}H\}$-NMR spectrum (201 MHz, *d*8-THF) of complex **^{13}C-3**. *: unknown impurities.

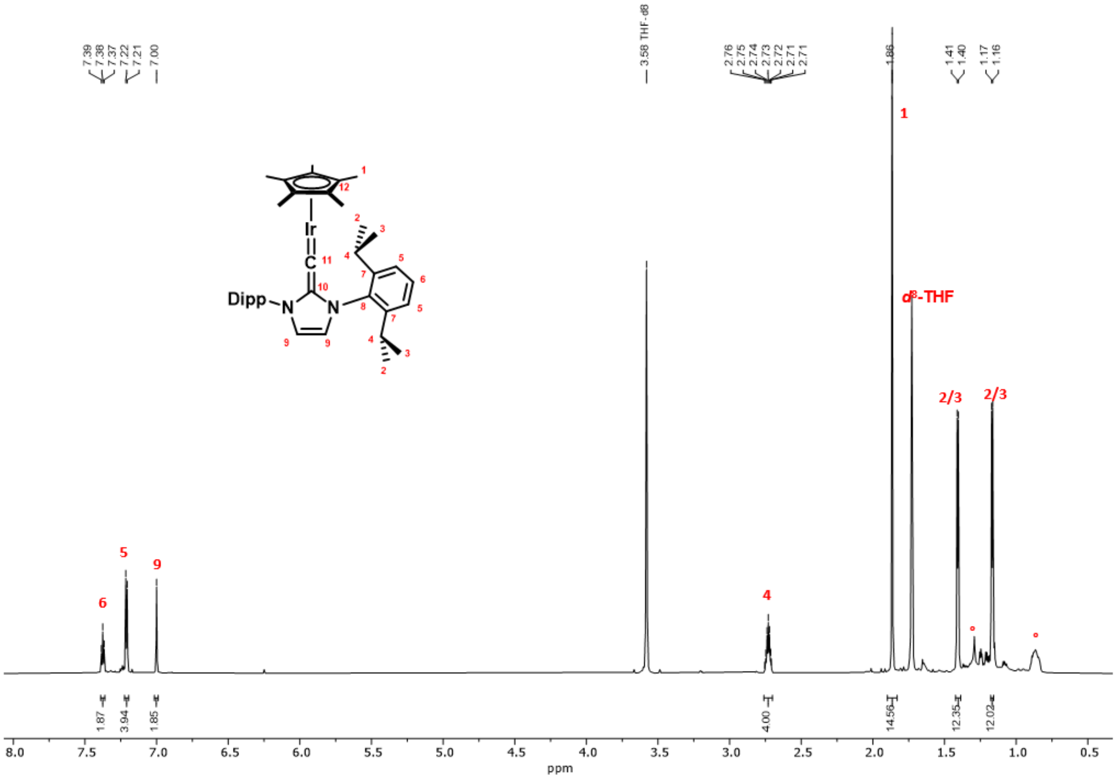

Figure S12 ^{1}H-NMR spectrum (800 MHz, *d*8-THF) of complex **5**. °: grease.

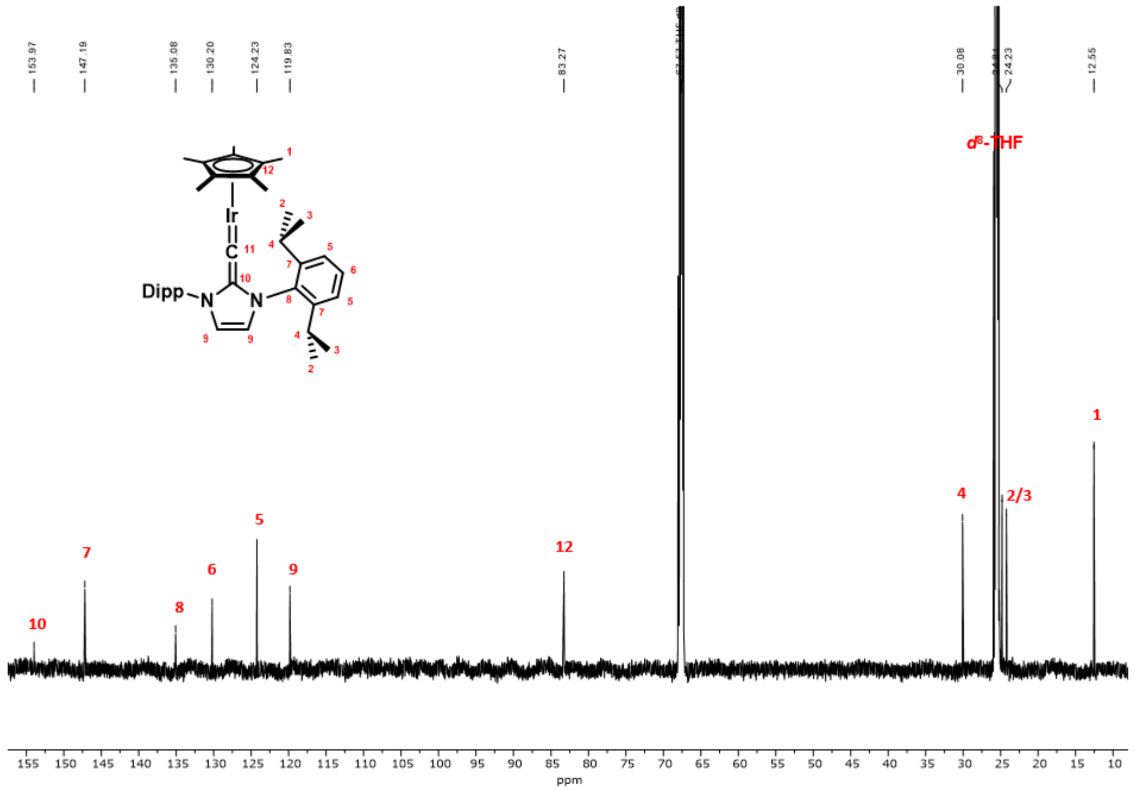

Figure S13 $^{13}C\{^{1}H\}$-NMR spectrum (201 MHz, d_8-THF) of complex **5**.

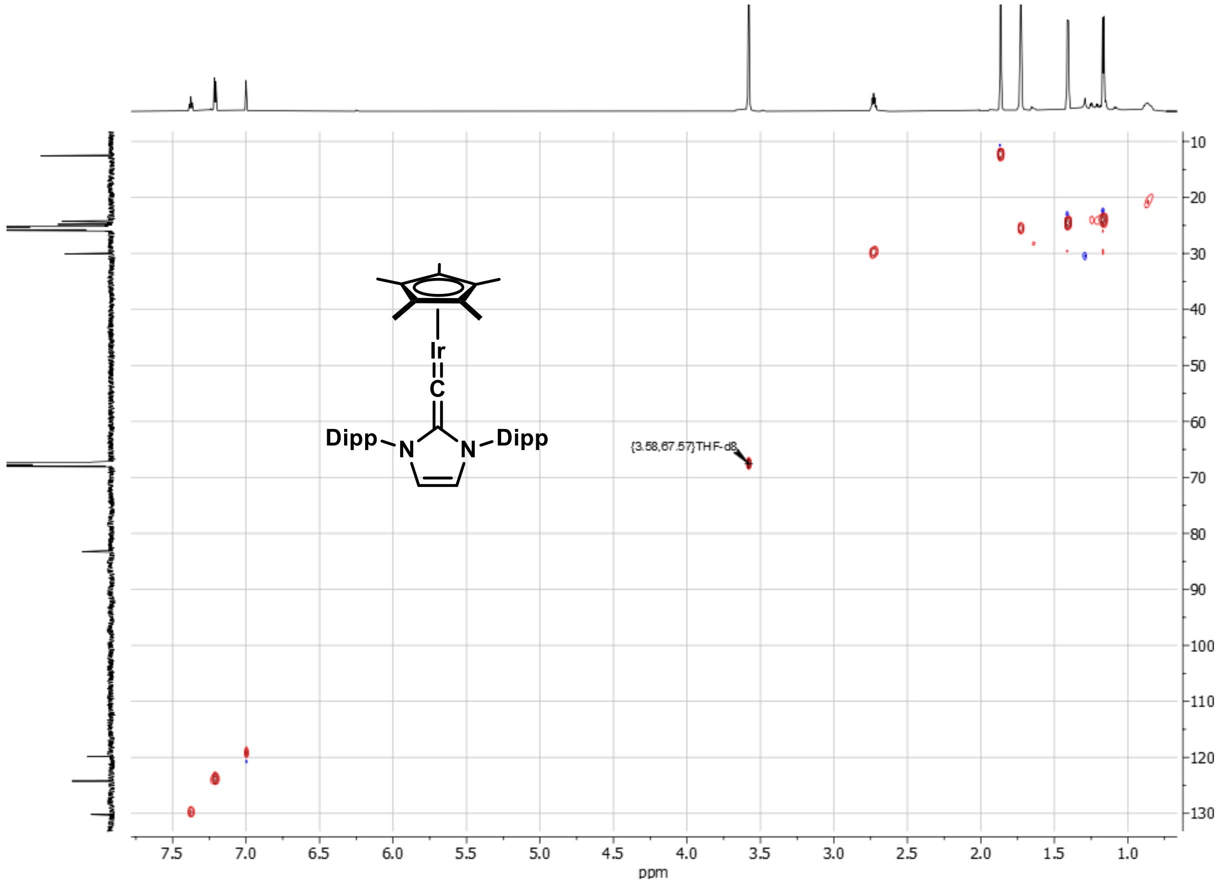

Figure S14 HSQC spectrum (201 MHz, d_8-THF) of complex **5**.

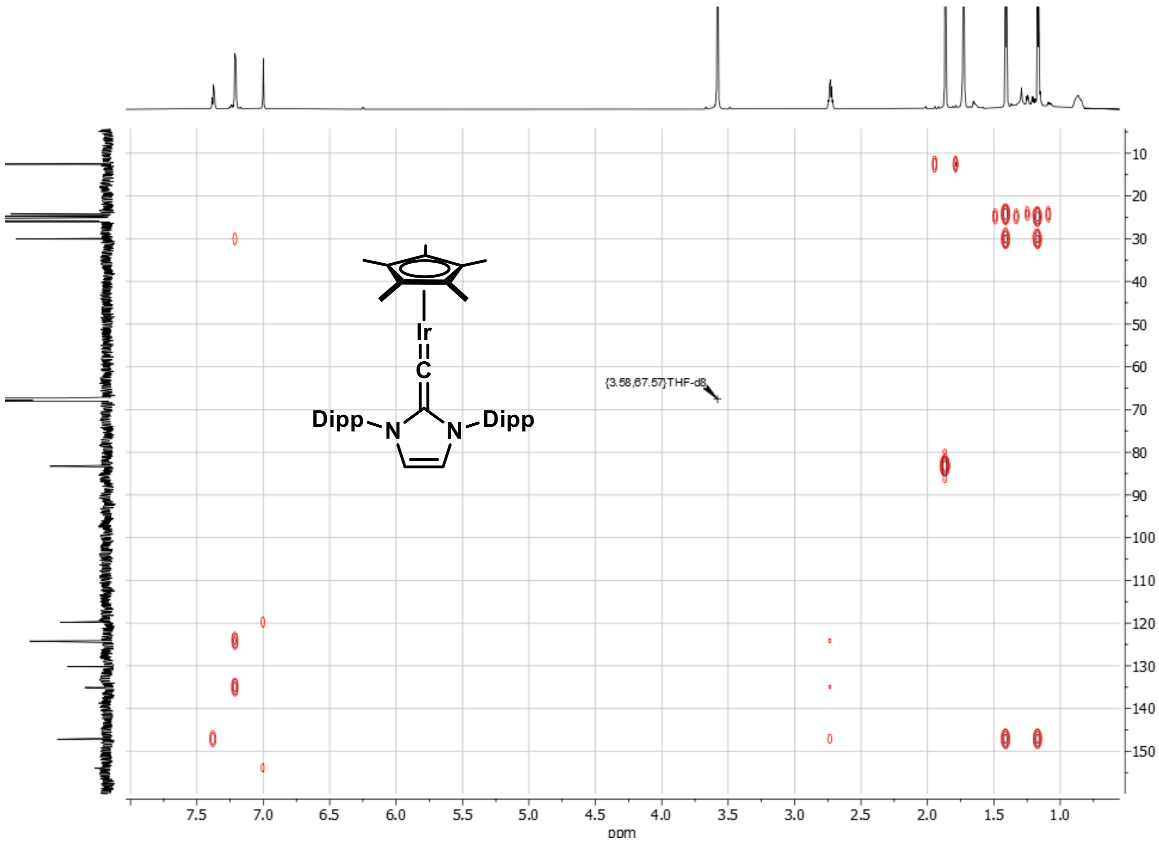

Figure S15 HMBC spectrum (201 MHz, d_8-THF) of complex **5**.

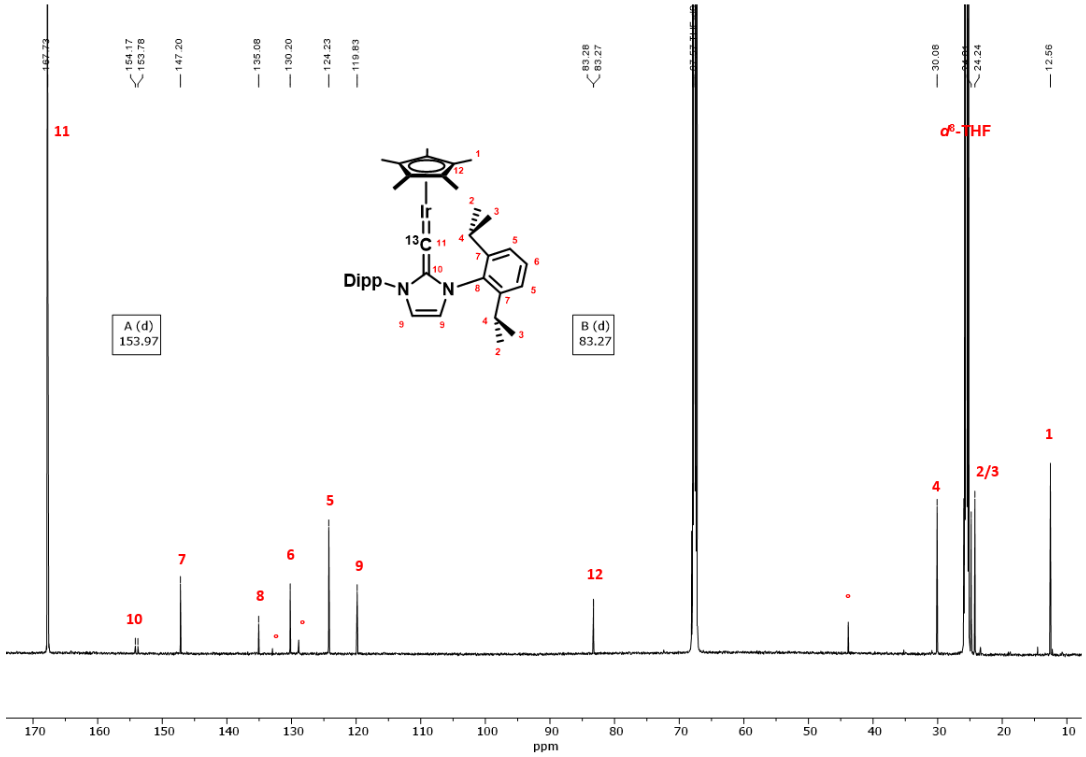

Figure S16 $^{13}C\{^1H\}$-NMR spectrum (151 MHz, d_8-THF) of complex **^{13}C-5**. The signal at 167.74 ppm indicated C=<u>C</u>=Ir. $^1J(^{13}C–C10)$ = 58.9 Hz, $^2J(^{13}C–C12)$ = 2.5 Hz. °: unknown impurities.

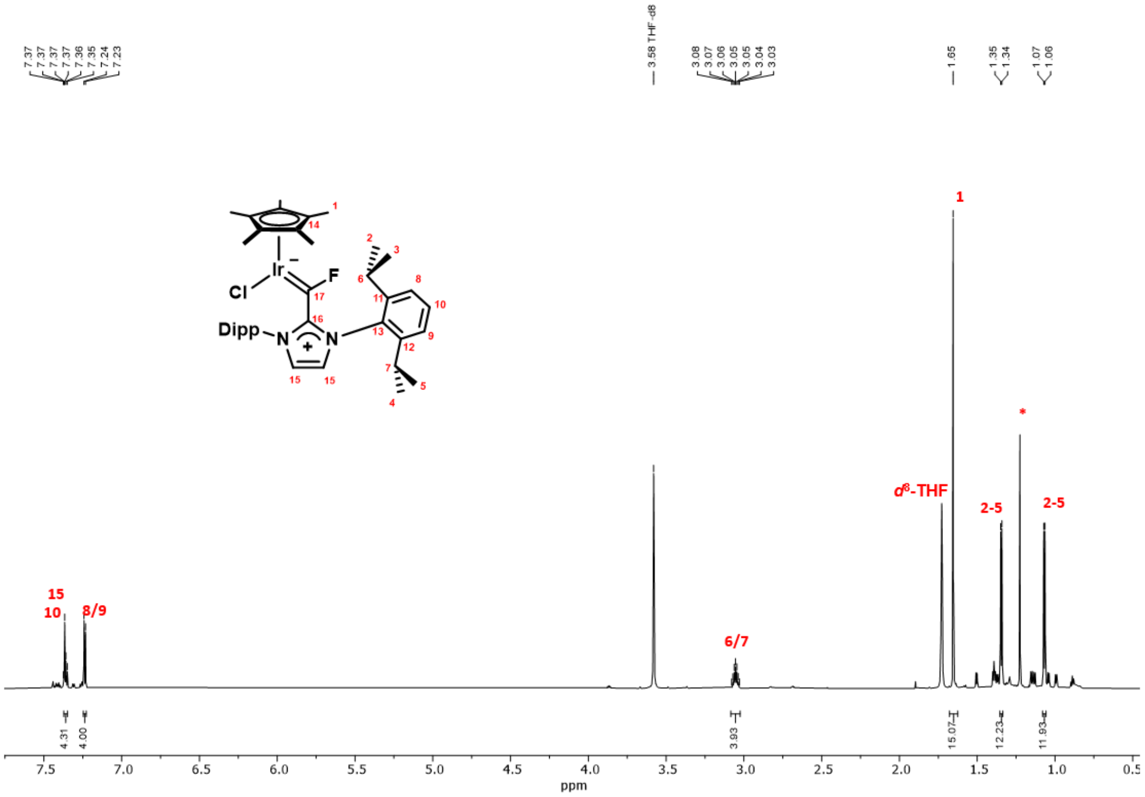

Figure S17 ^{1}H-NMR spectrum (800 MHz, d_8-THF) of complex **6**. *: unknown impurities.

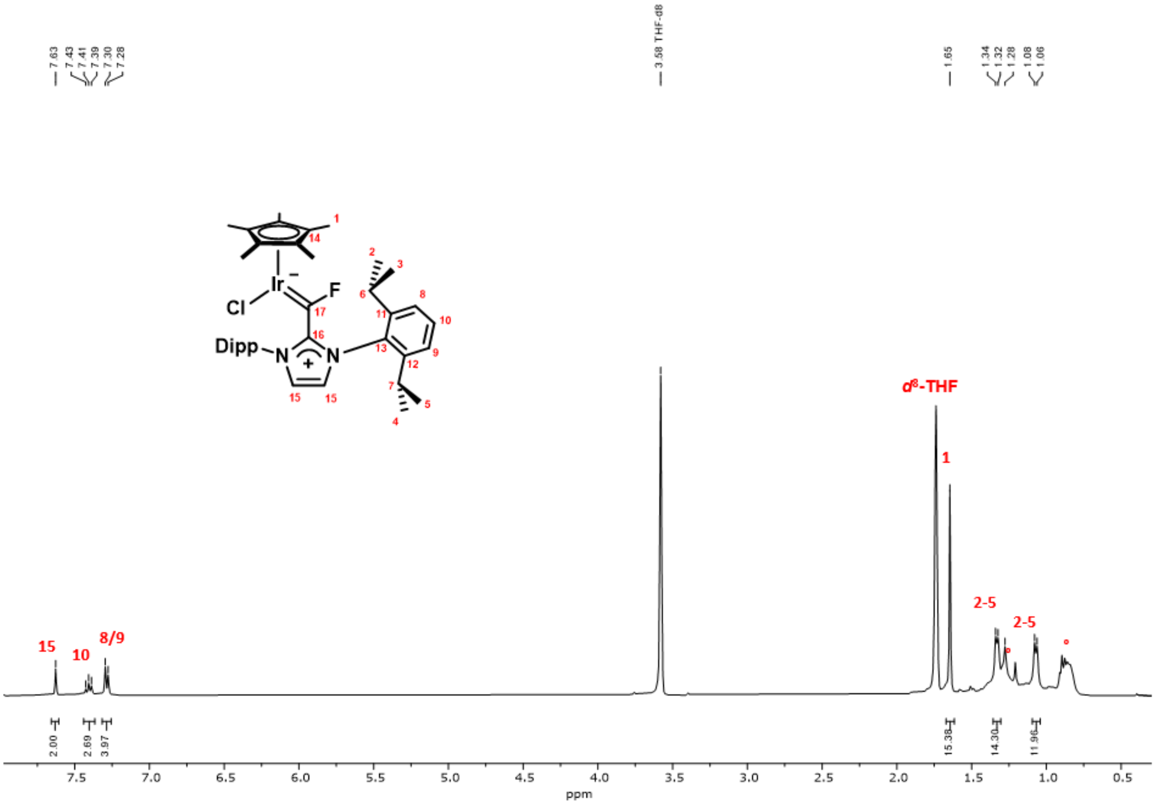

Figure S18 ^{1}H-NMR spectrum (400 MHz, d_8-THF, –50 °C) of complex **6**. °: pentane/grease..

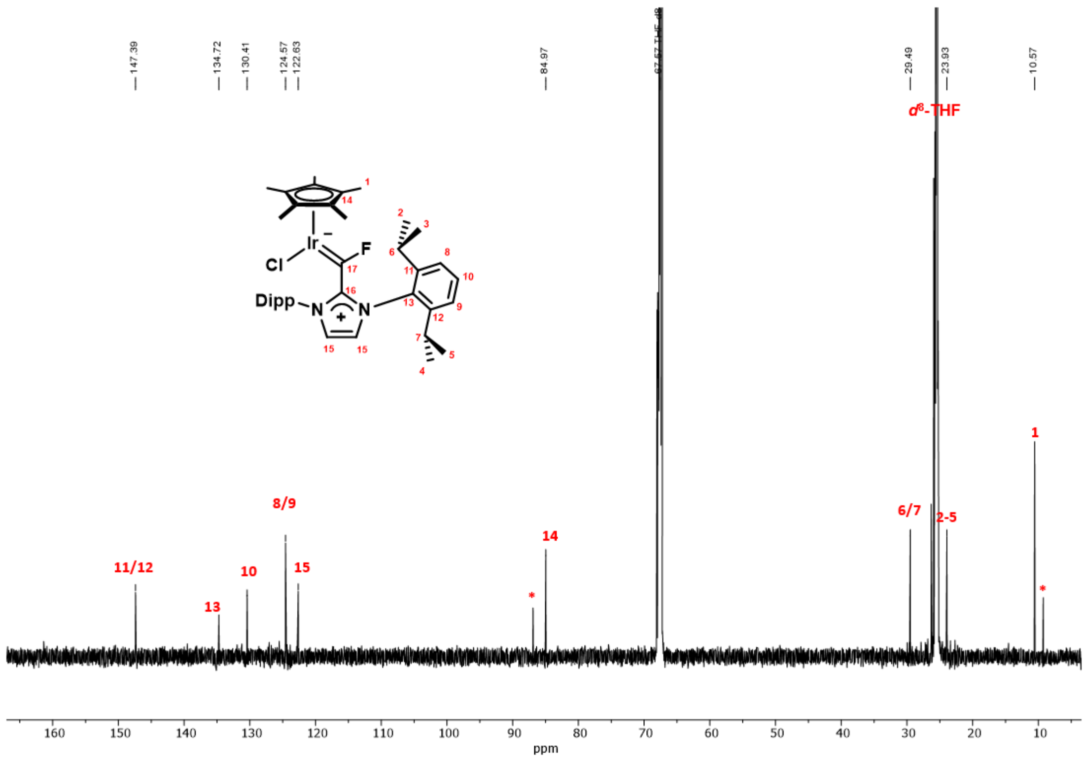

Figure S19 $^{13}C\{^1H\}$-NMR spectrum (201 MHz, d_8-THF) of complex **6**. *: decomposed product.

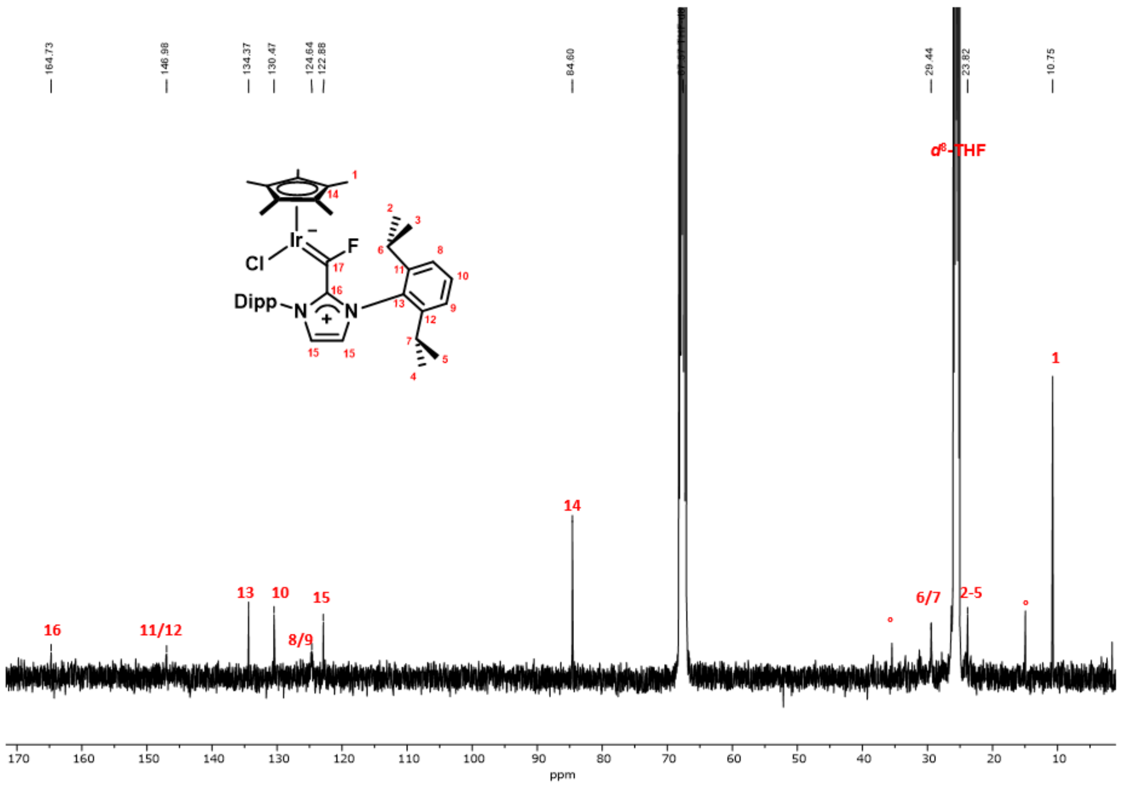

Figure S20 $^{13}C\{^1H\}$-NMR spectrum (101 MHz, d_8-THF, –50 °C) of complex **6**. °: pentane.

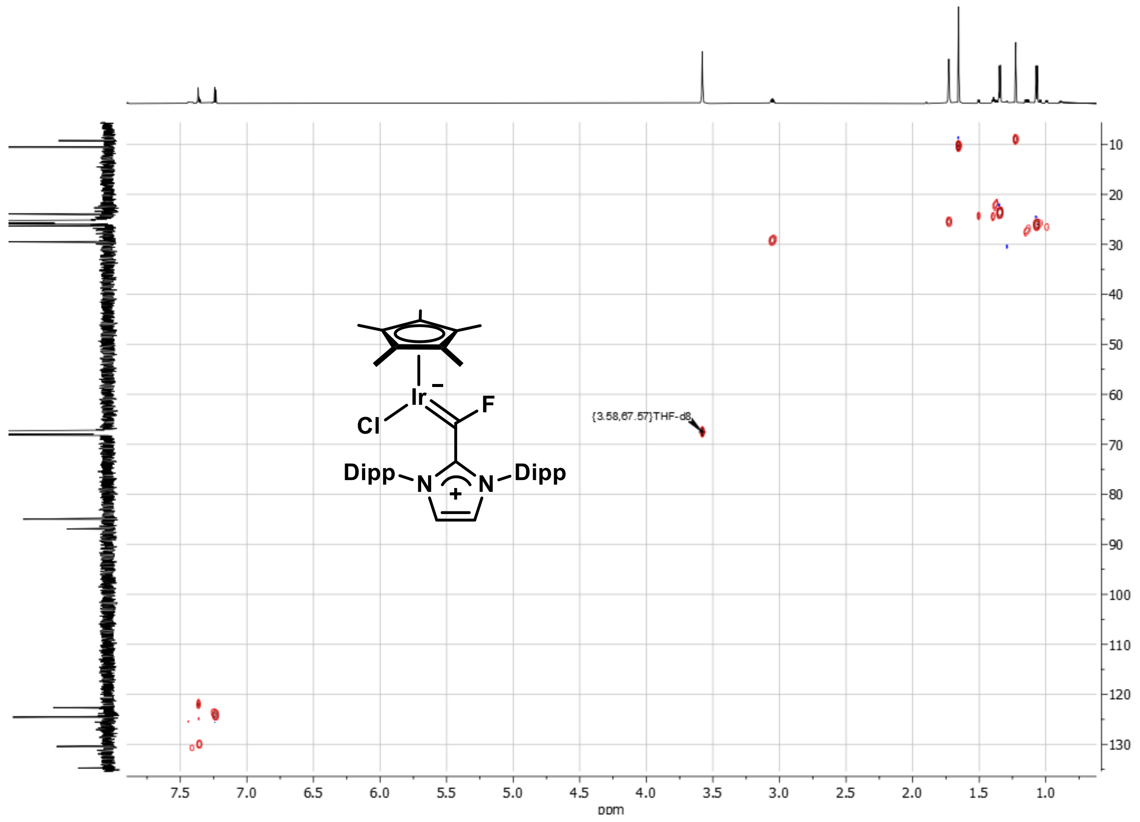

Figure S21 HSQC spectrum (201 MHz, *d*8-THF) of complex **6**.

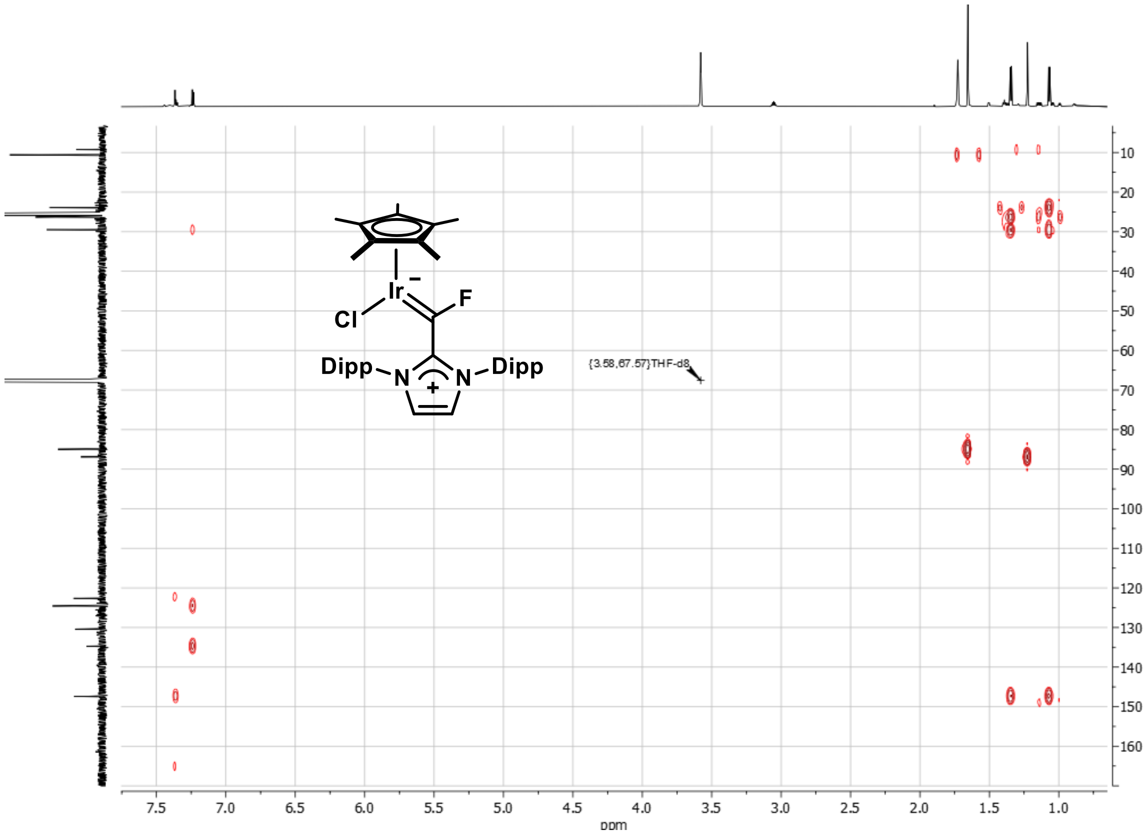

Figure S22 HSQC spectrum (201 MHz, *d*8-THF) of complex **6**.

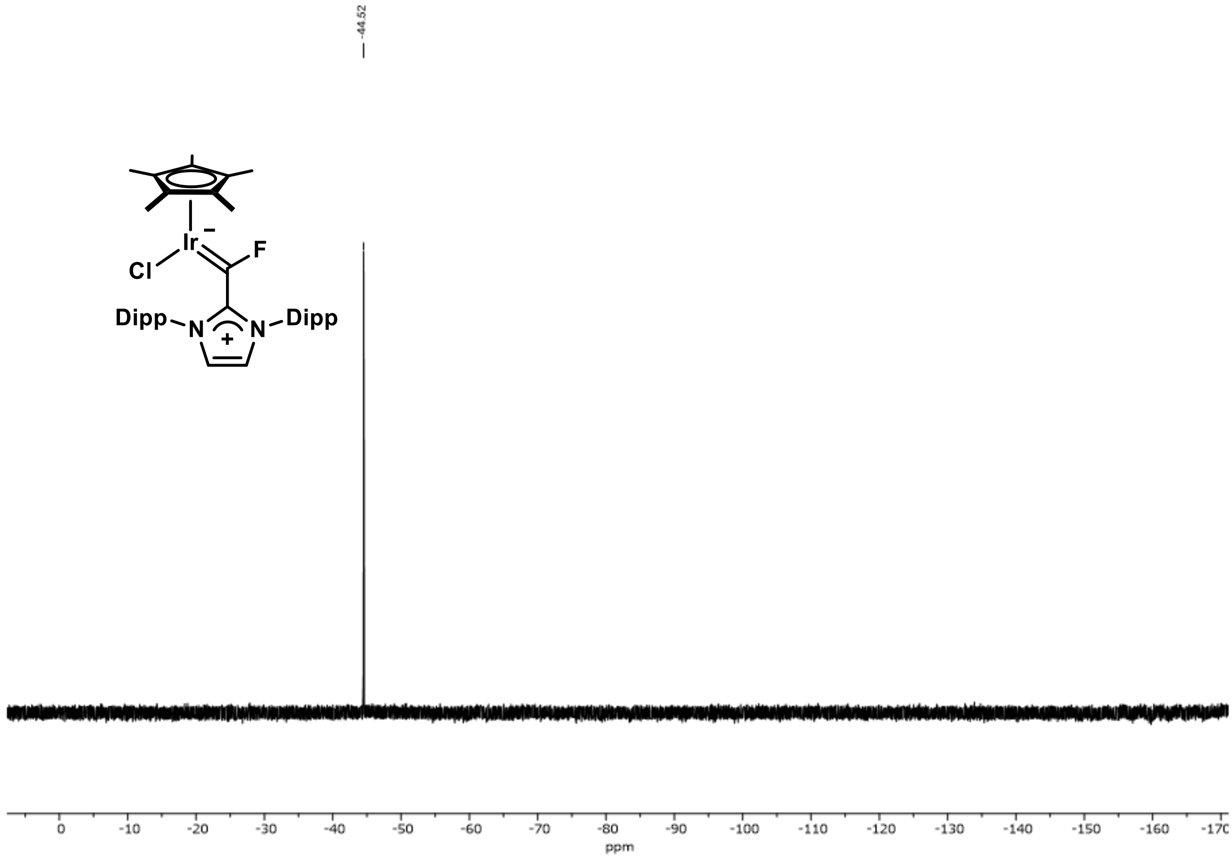

Figure S23 $^{19}F\{^{1}H\}$-NMR spectrum (376 MHz, d_8-THF, –50 °C) of complex **6**.

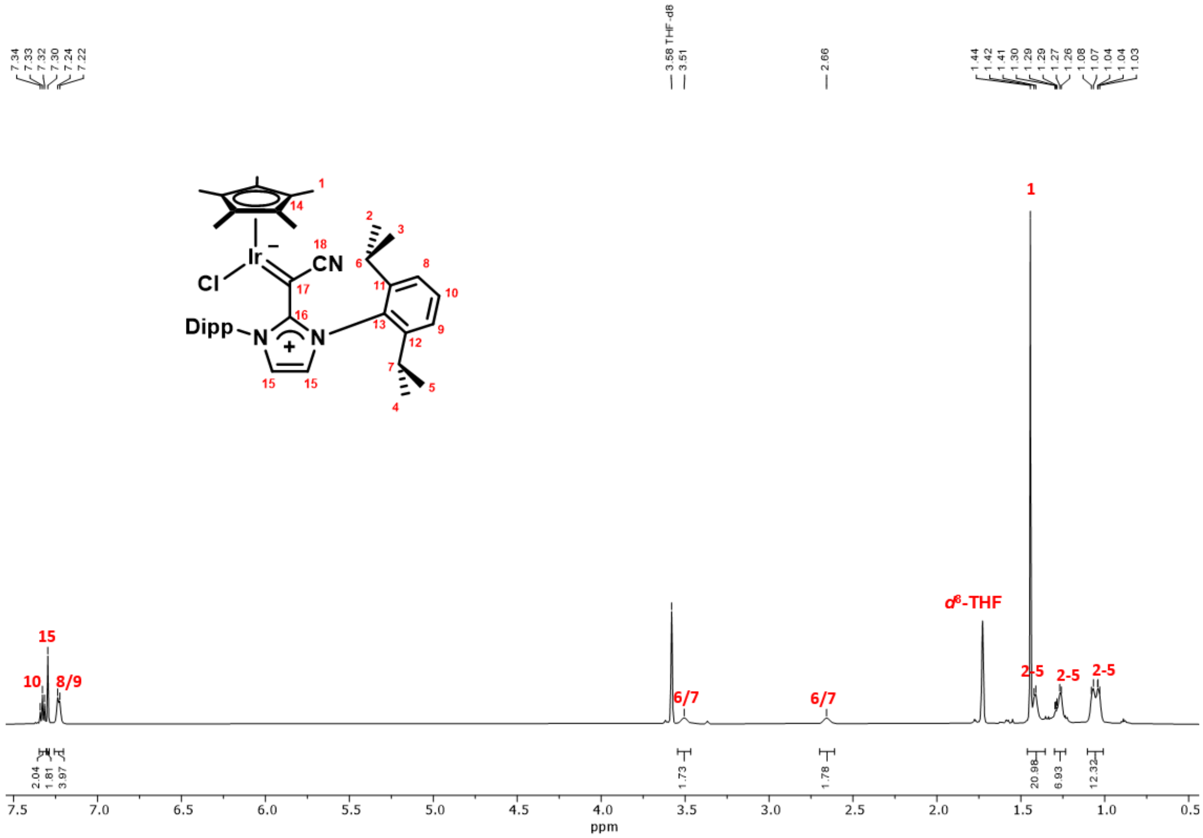

Figure S24 ^{1}H-NMR spectrum (600 MHz, d_8-THF) of complex **7**.

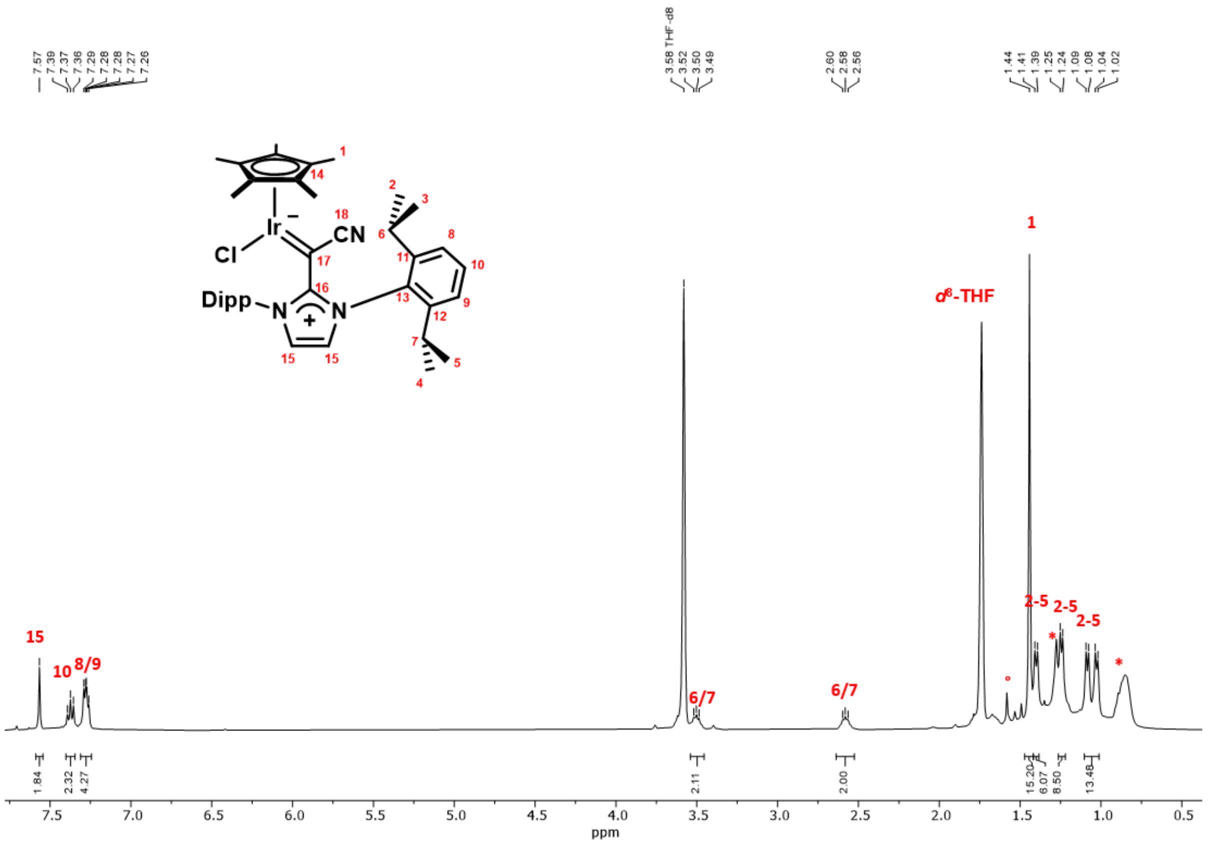

Figure S25 ^{1}H-NMR spectrum (400 MHz, d_8-THF, –50 °C) of complex **7**. *: grease, °: unknown impurities.

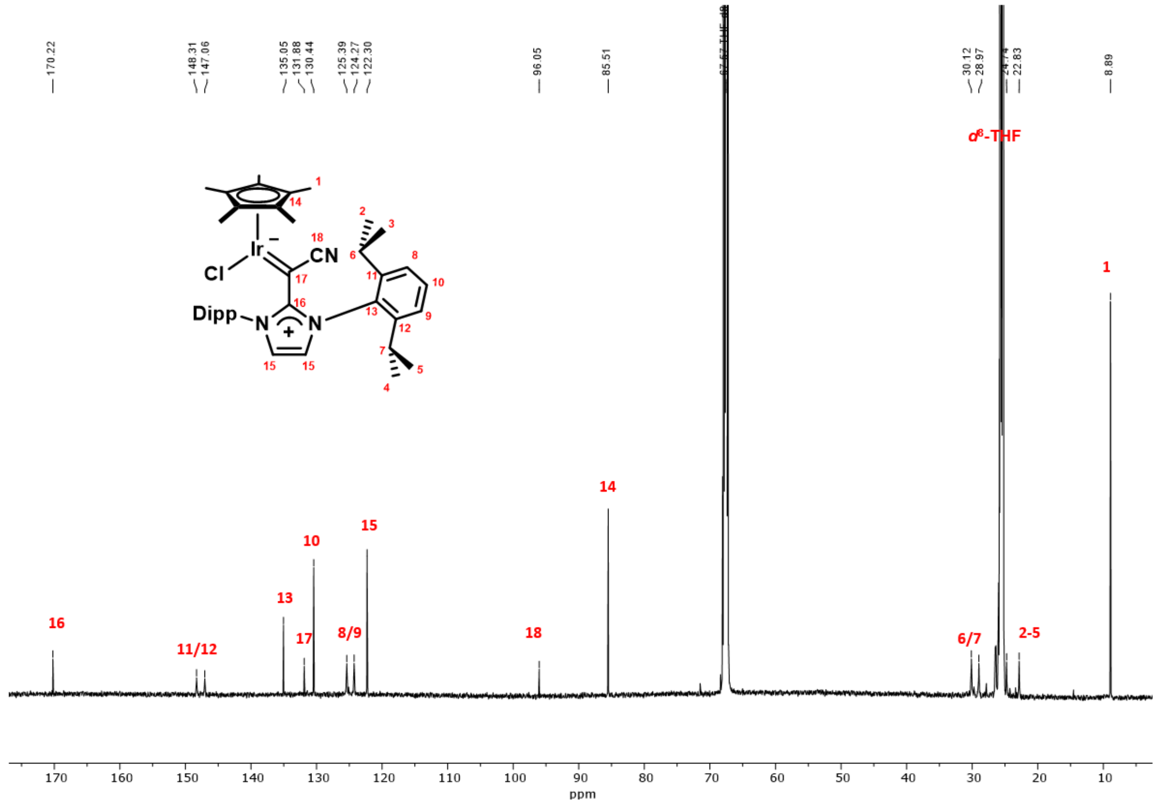

Figure S26 ^{13}C{^{1}H}-NMR spectrum (151 MHz, d_8-THF) of complex **7**.

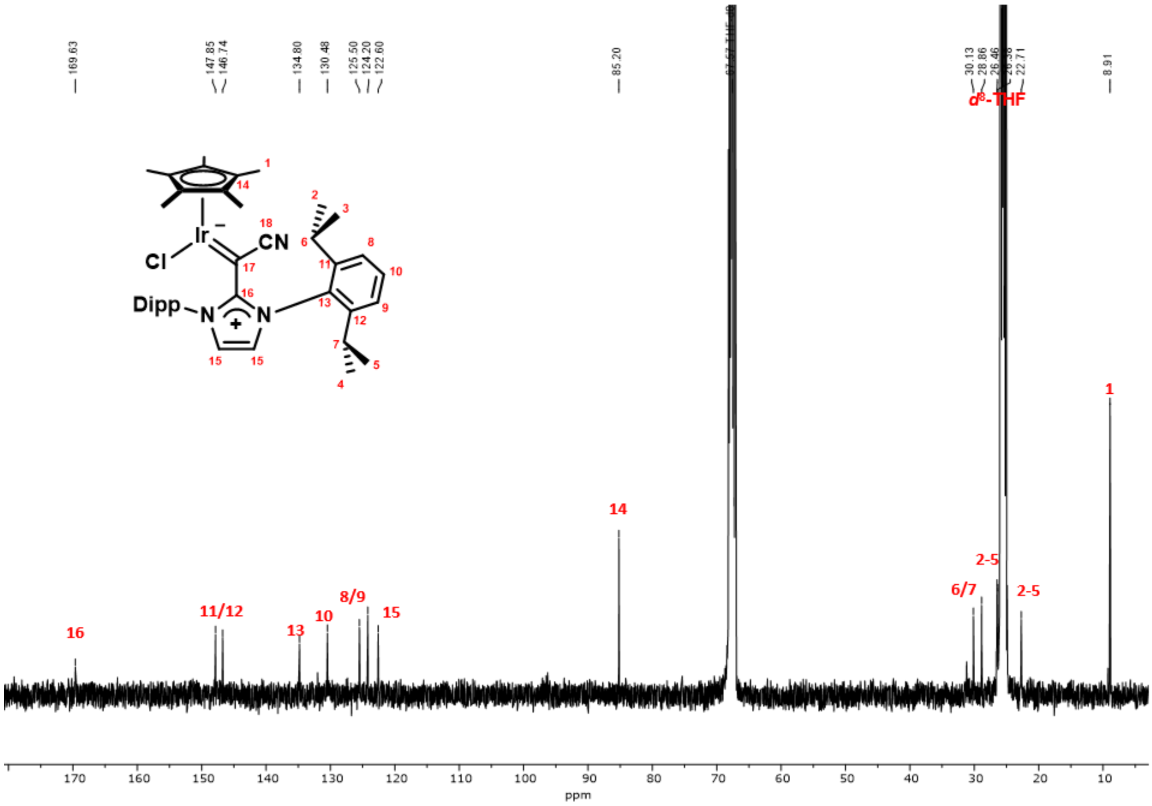

Figure S27 $^{13}C\{^1H\}$-NMR spectrum (101 MHz, *d*8-THF, –50 °C) of complex **7**.

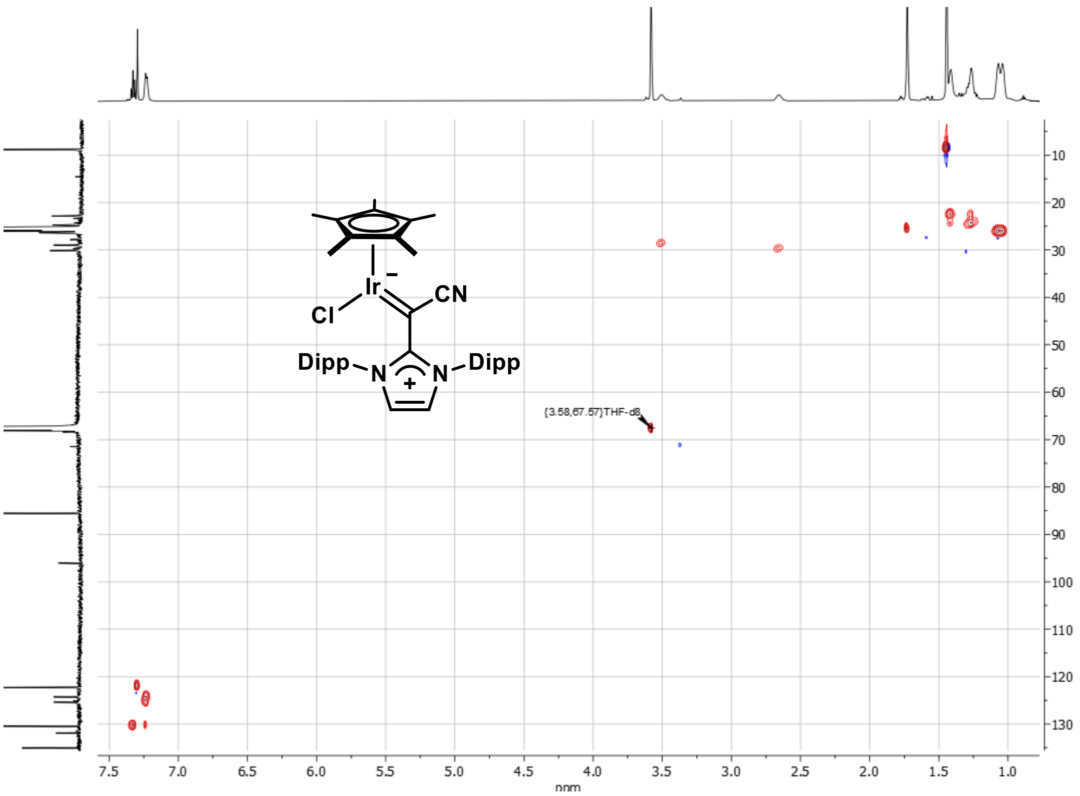

Figure S28 HSQC spectrum (201 MHz, *d*8-THF) of complex **7**.

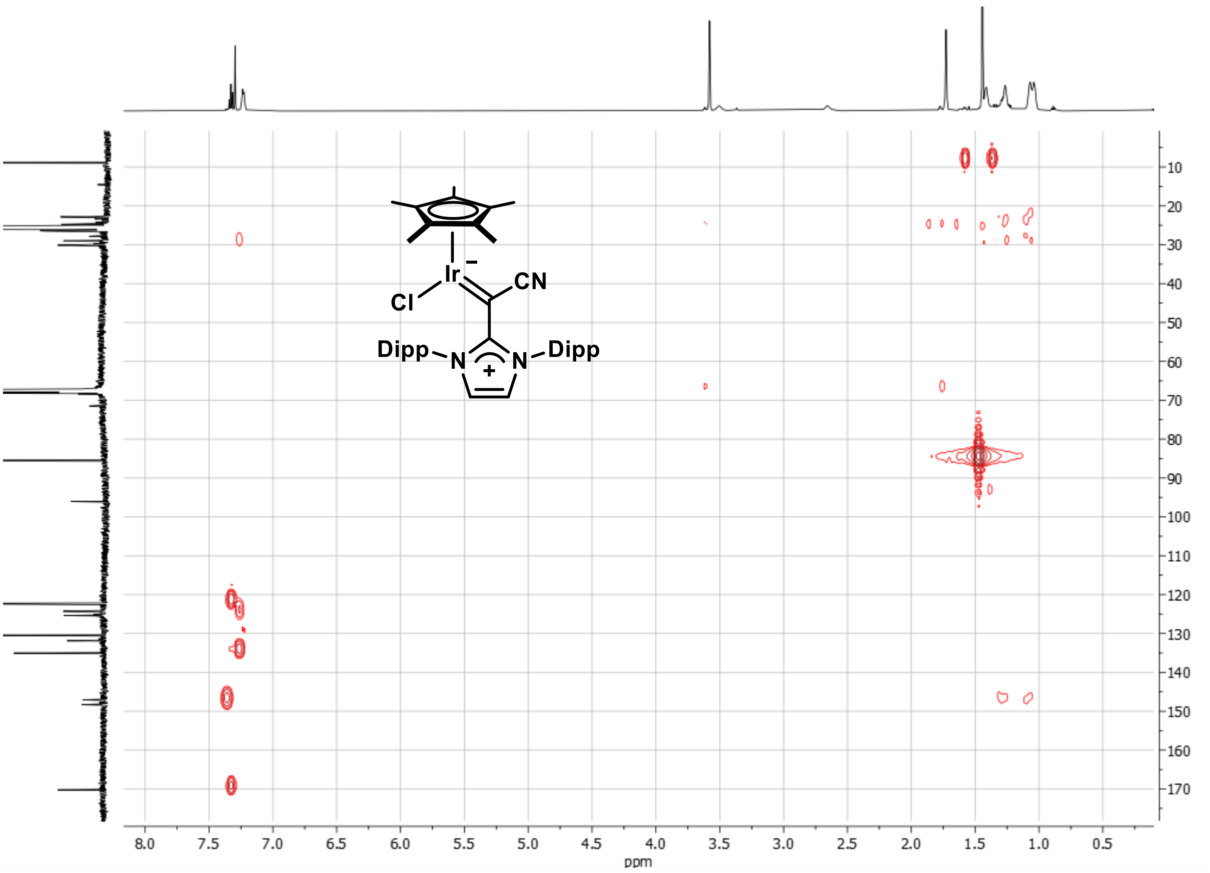

Figure S29 HMBC spectrum (151 MHz, d_8-THF) of complex **7**.

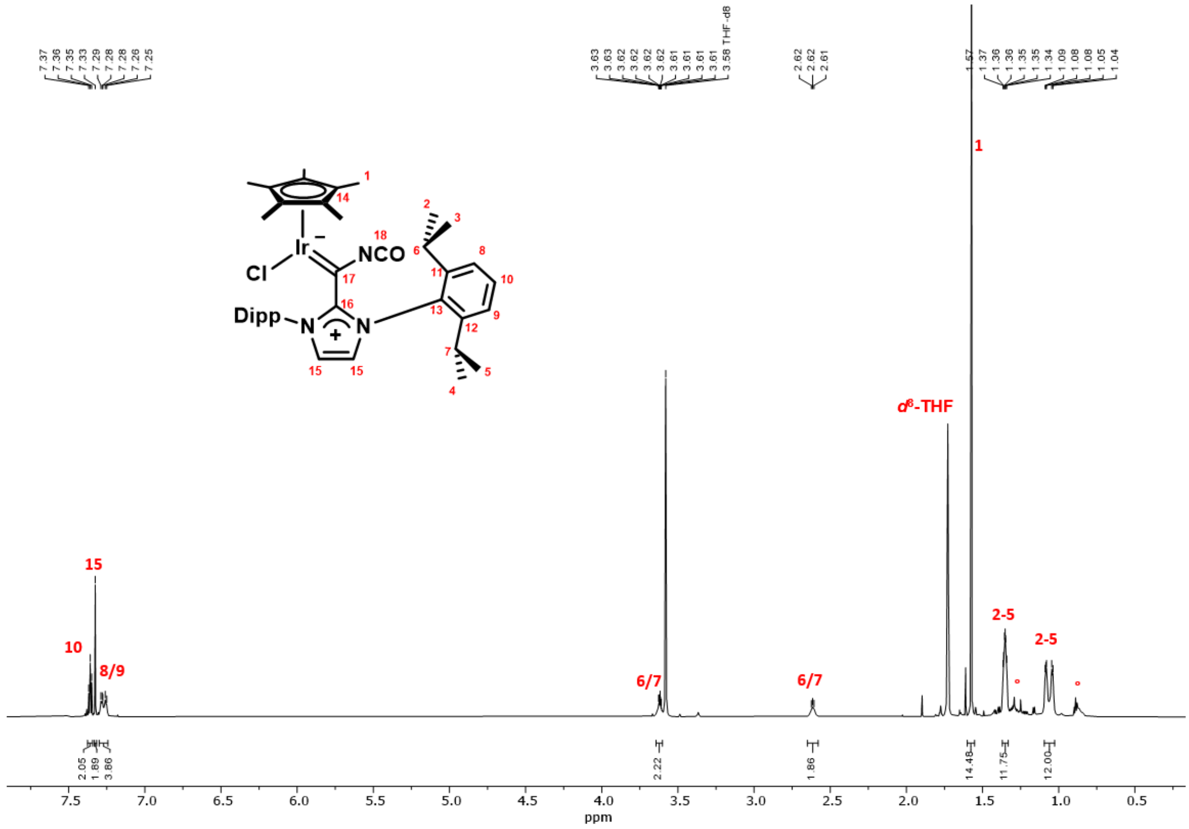

Figure S30 ^{1}H-NMR spectrum (800 MHz, d_8-THF) of complex **8**. °: grease.

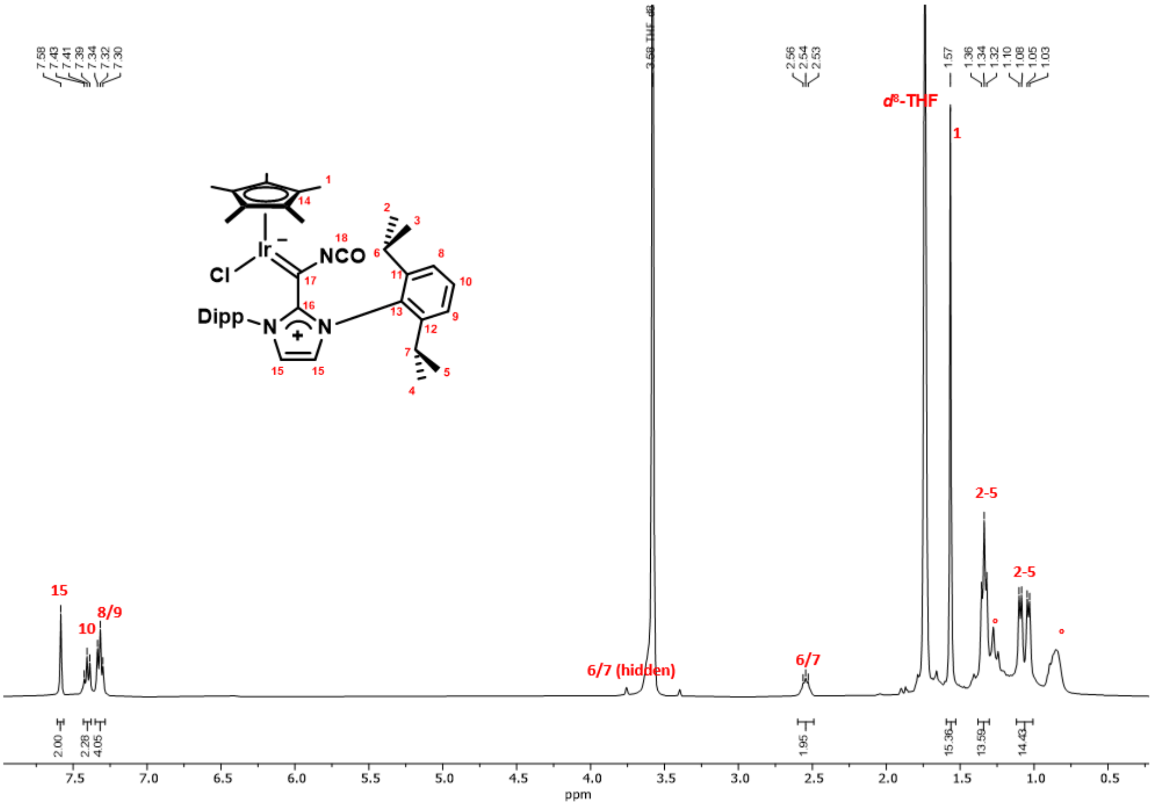

Figure S31 ^{1}H-NMR spectrum (400 MHz, *d*$_8$-THF, –50 °C) of complex **8**. °: grease.

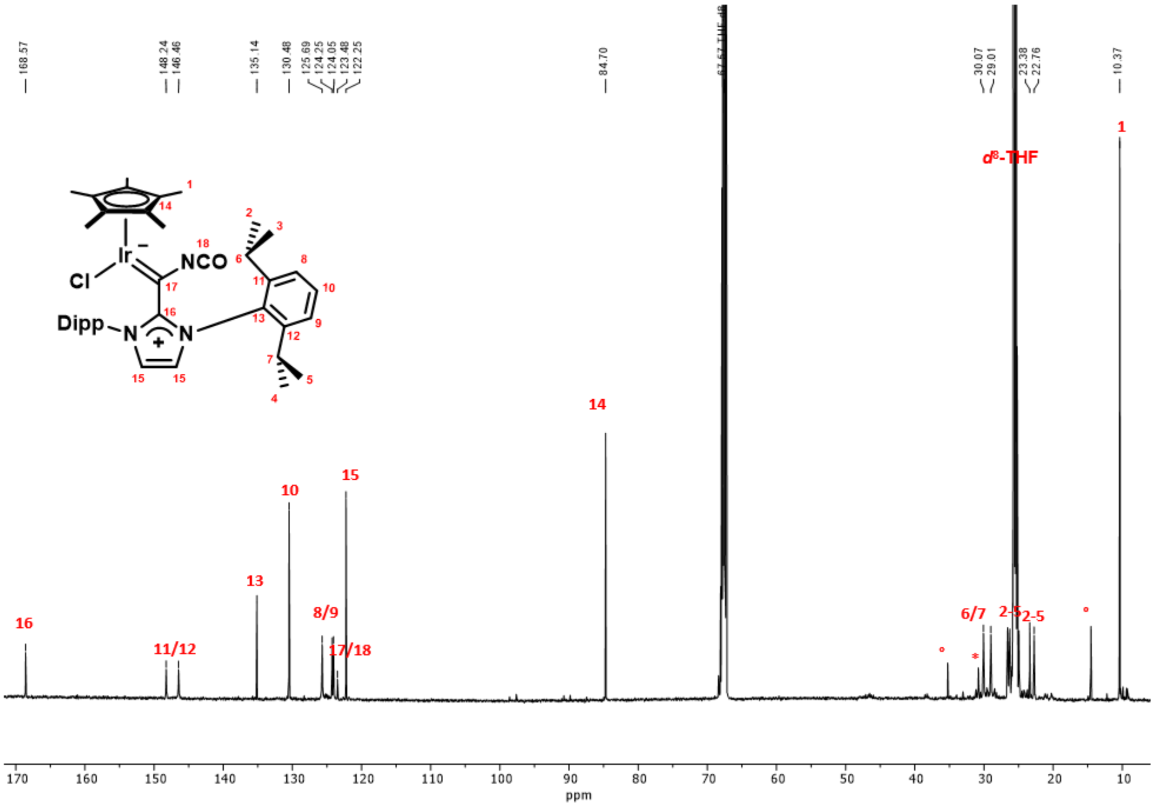

Figure S32 ^{13}C{^{1}H}-NMR spectrum (151 MHz, *d*$_8$-THF) of complex **8**. °: pentane, *: unknown impurities.

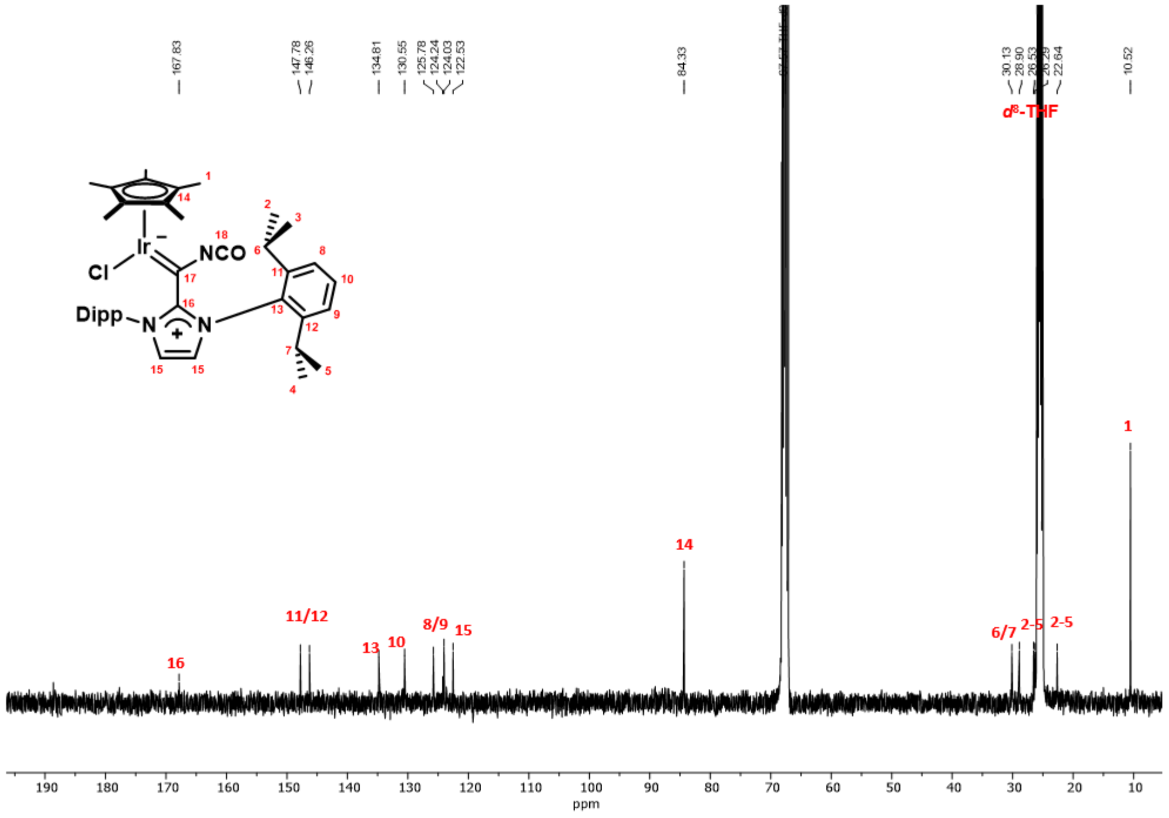

Figure S33 $^{13}C\{^1H\}$-NMR spectrum (101 MHz, *d*8-THF, –50 °C) of complex **8**.

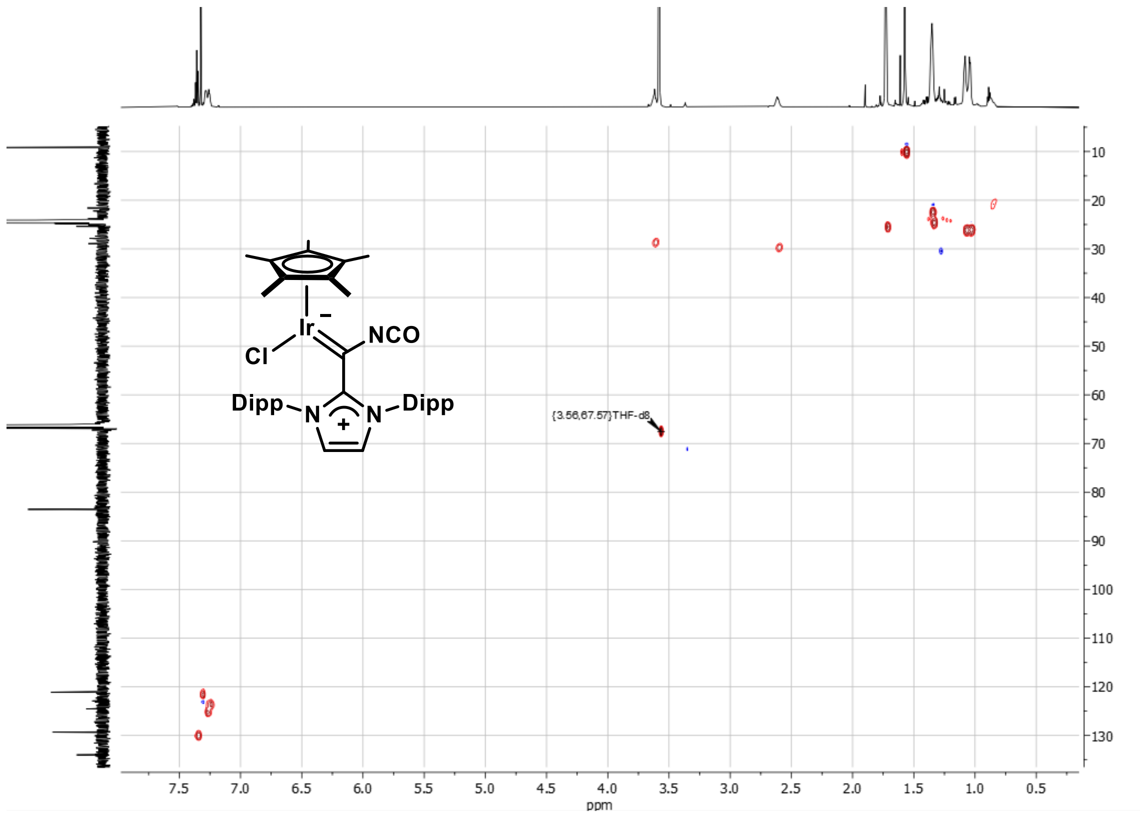

Figure S34 HSQC spectrum (201 MHz, *d*8-THF) of complex **8**.

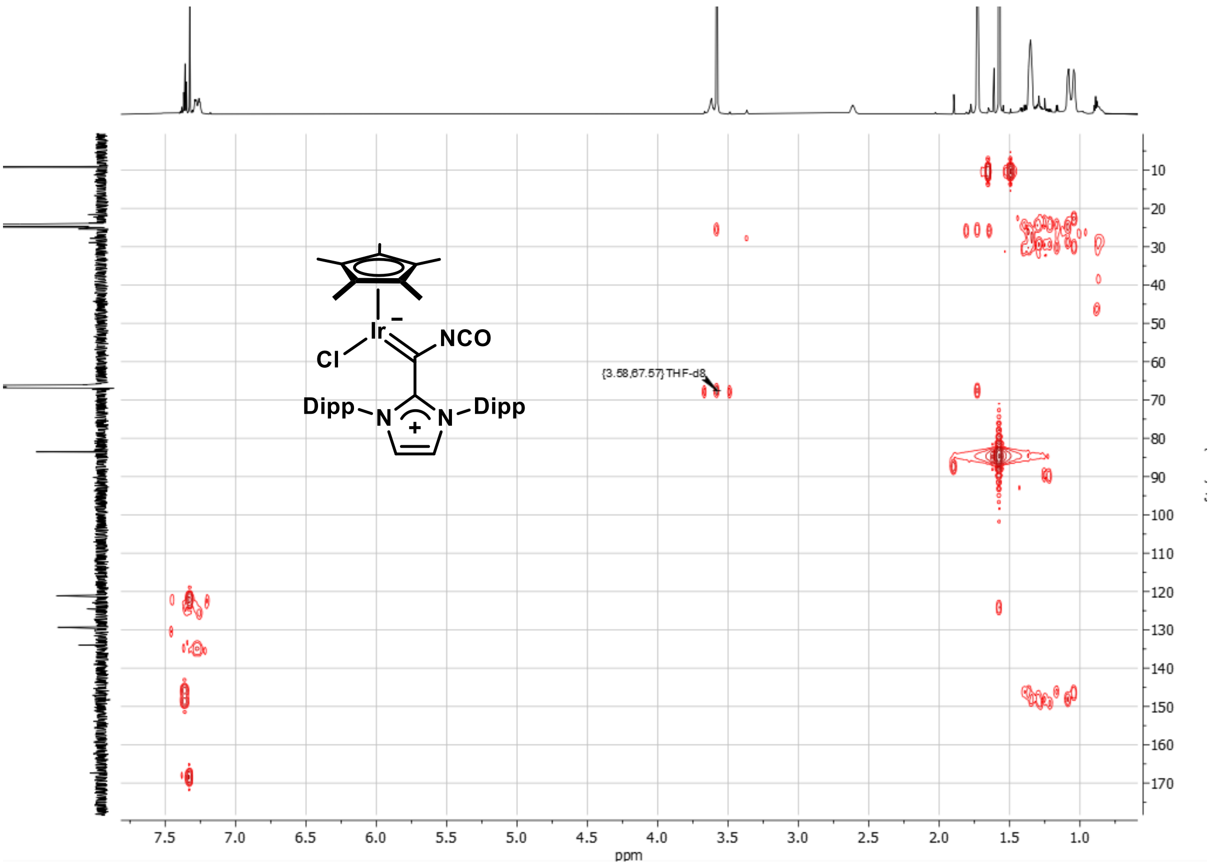

Figure S35 HMBC spectrum (201 MHz, d_8-THF) of complex **8**.

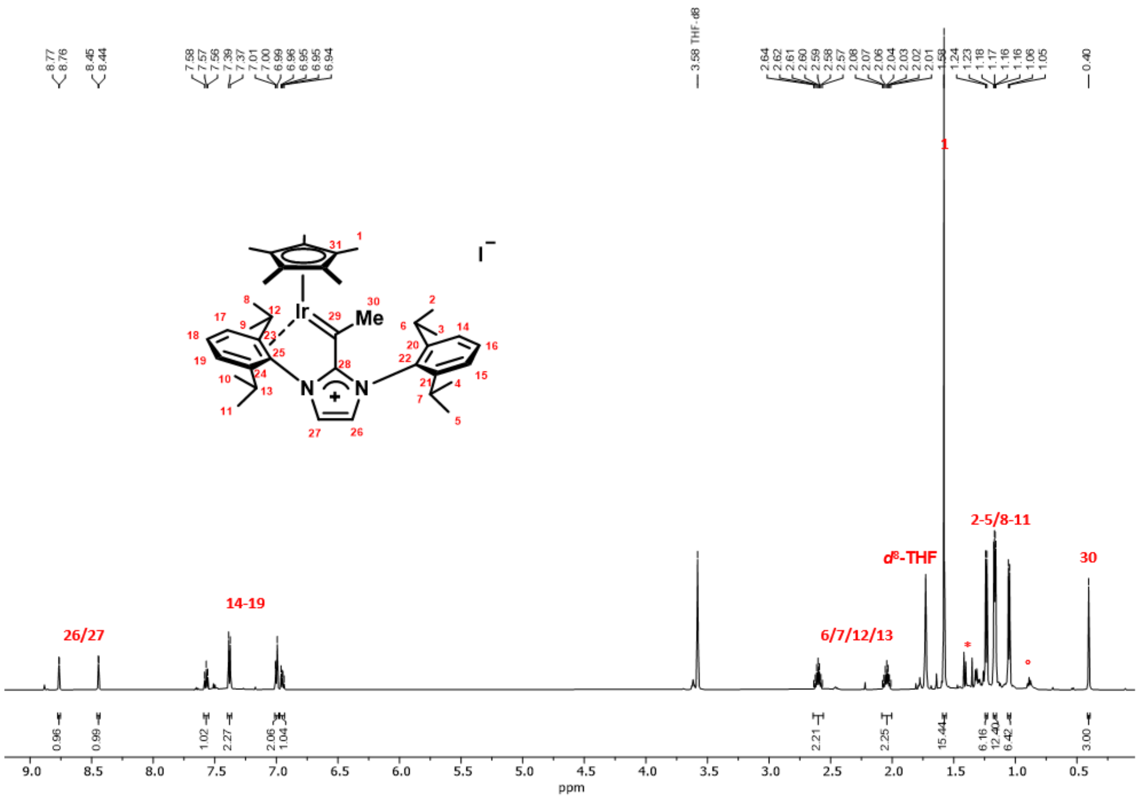

Figure S36 ^{1}H-NMR spectrum (600 MHz, d_8-THF) of complex **9**. °: pentane, *: unknown impurities.

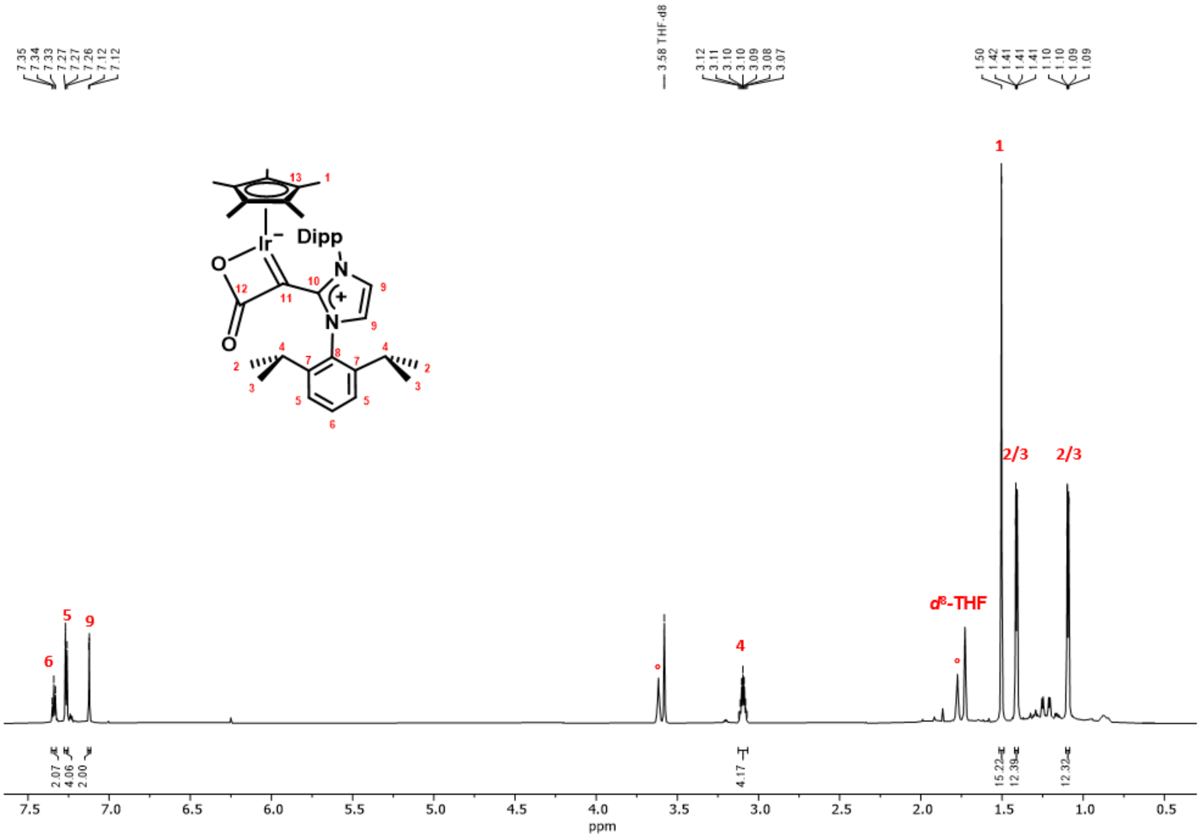

Figure S37 ^{1}H-NMR spectrum (800 MHz, d_8-THF) of complex **10**. °: THF.

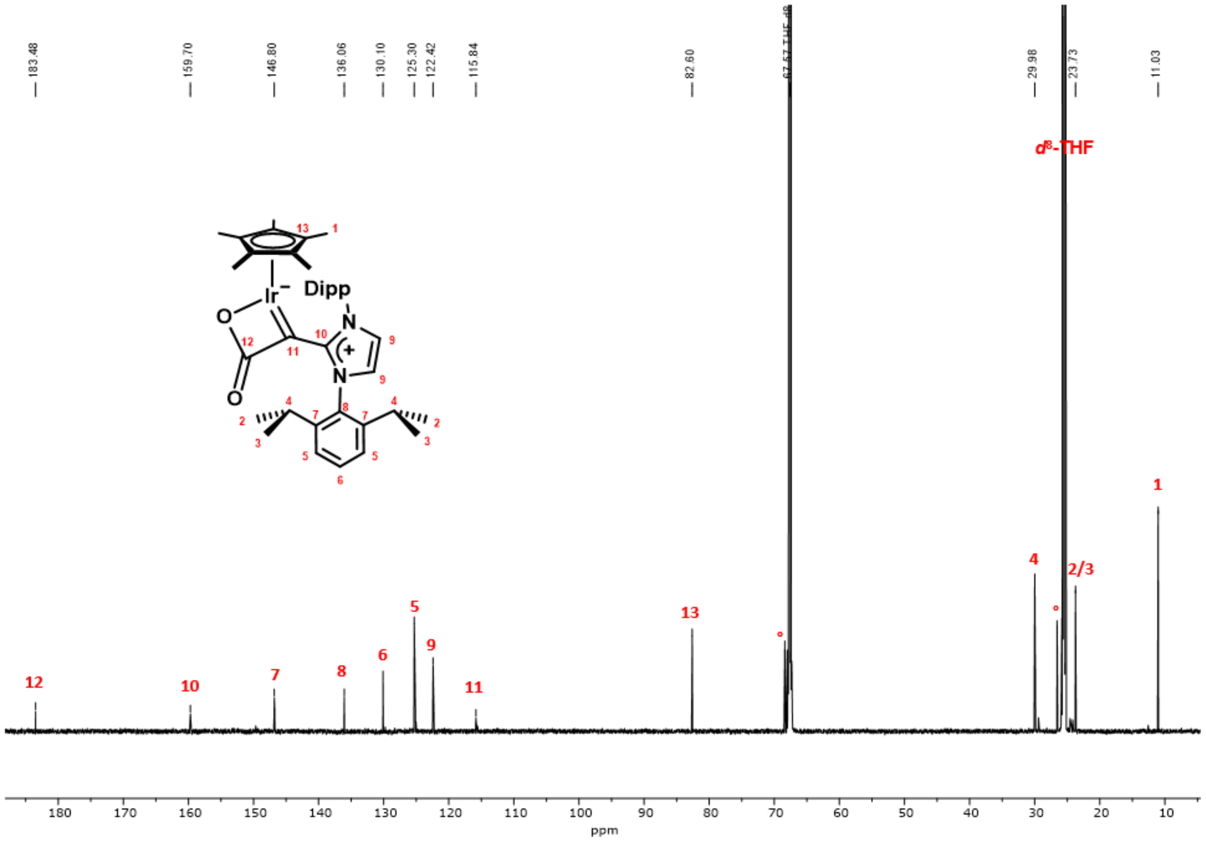

Figure S38 ^{13}C{^{1}H}-NMR spectrum (201 MHz, d_8-THF) of complex **10**. °: THF.

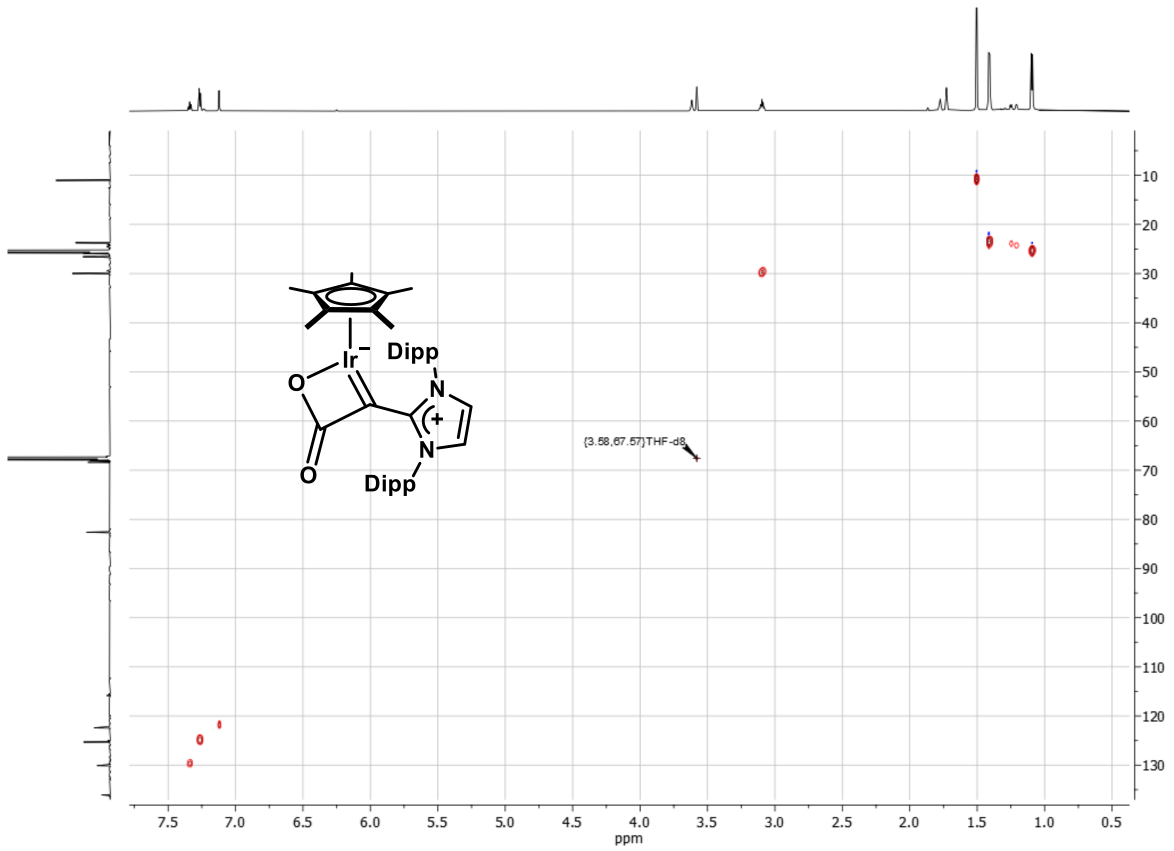

Figure S39 HSQC spectrum (201 MHz, *d*8-THF) of complex **10**.

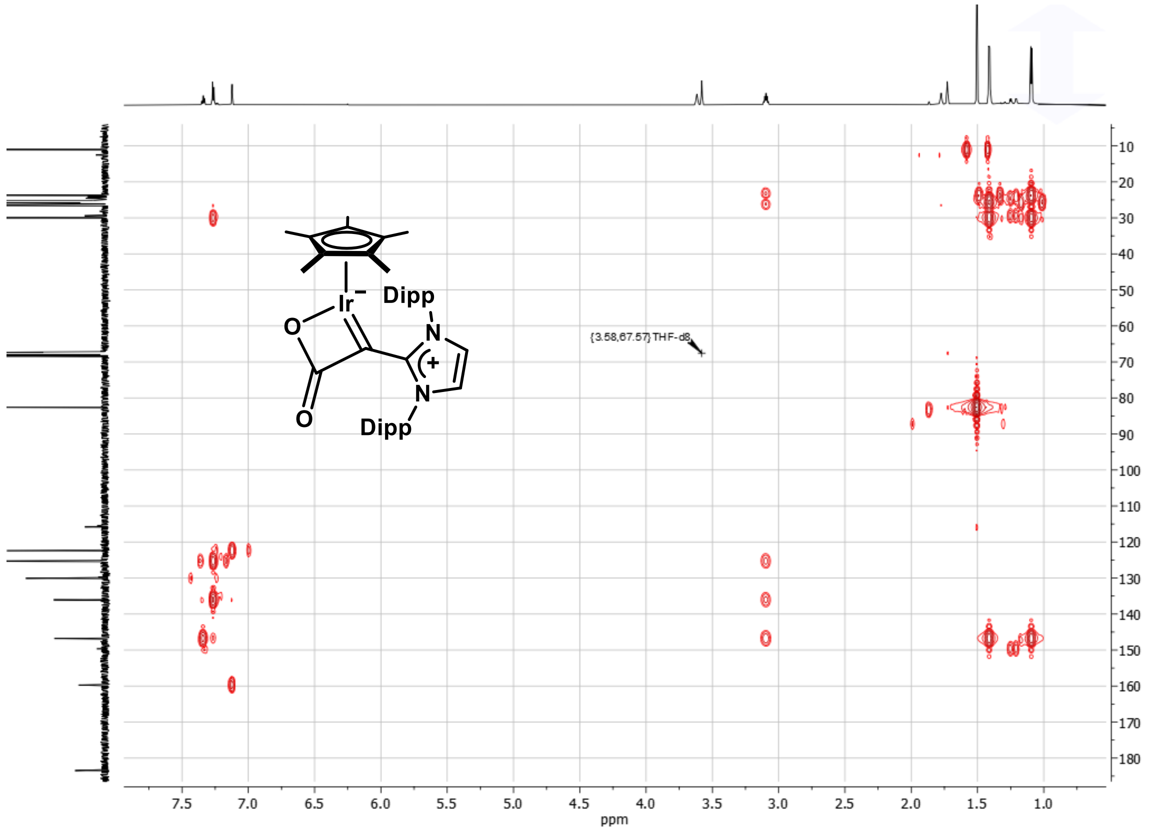

Figure S40 HMBC spectrum (201 MHz, *d*8-THF) of complex **10**.

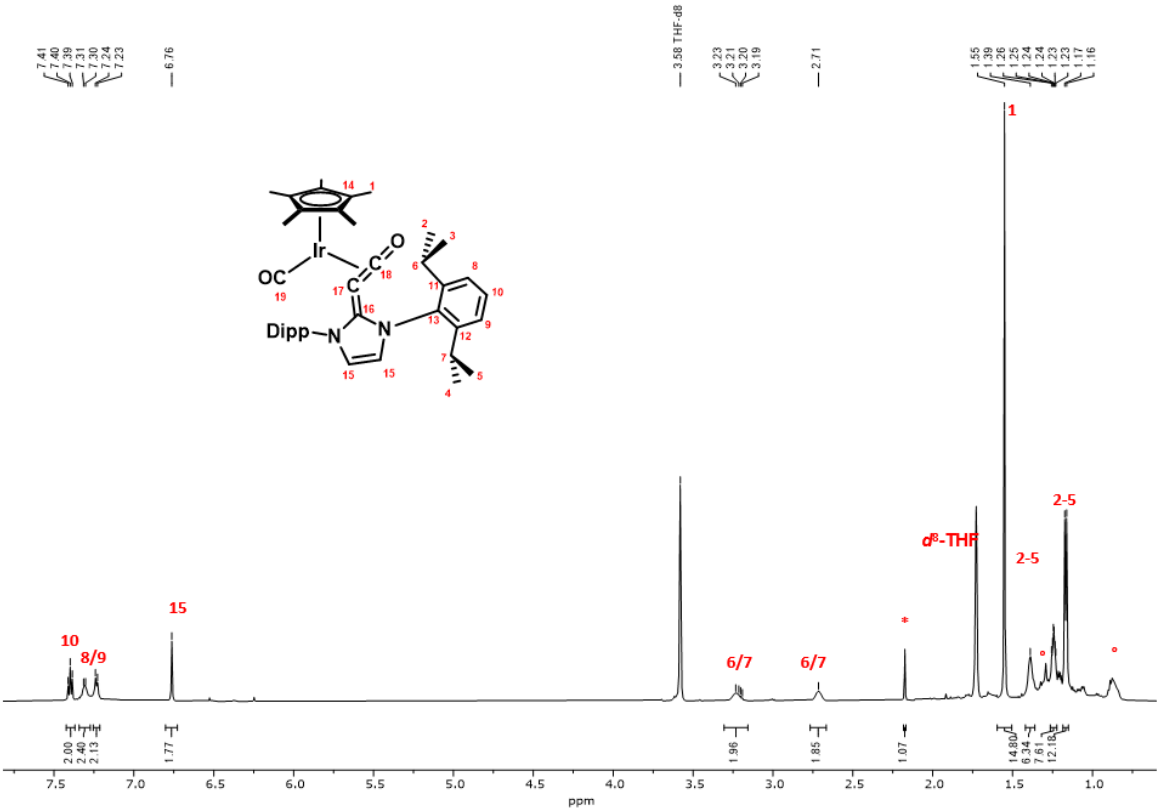

Figure S41 ^{1}H-NMR spectrum (600 MHz, d_8-THF) of complex **11**. *: Cp*Ir(CO)$_2$, °: grease.

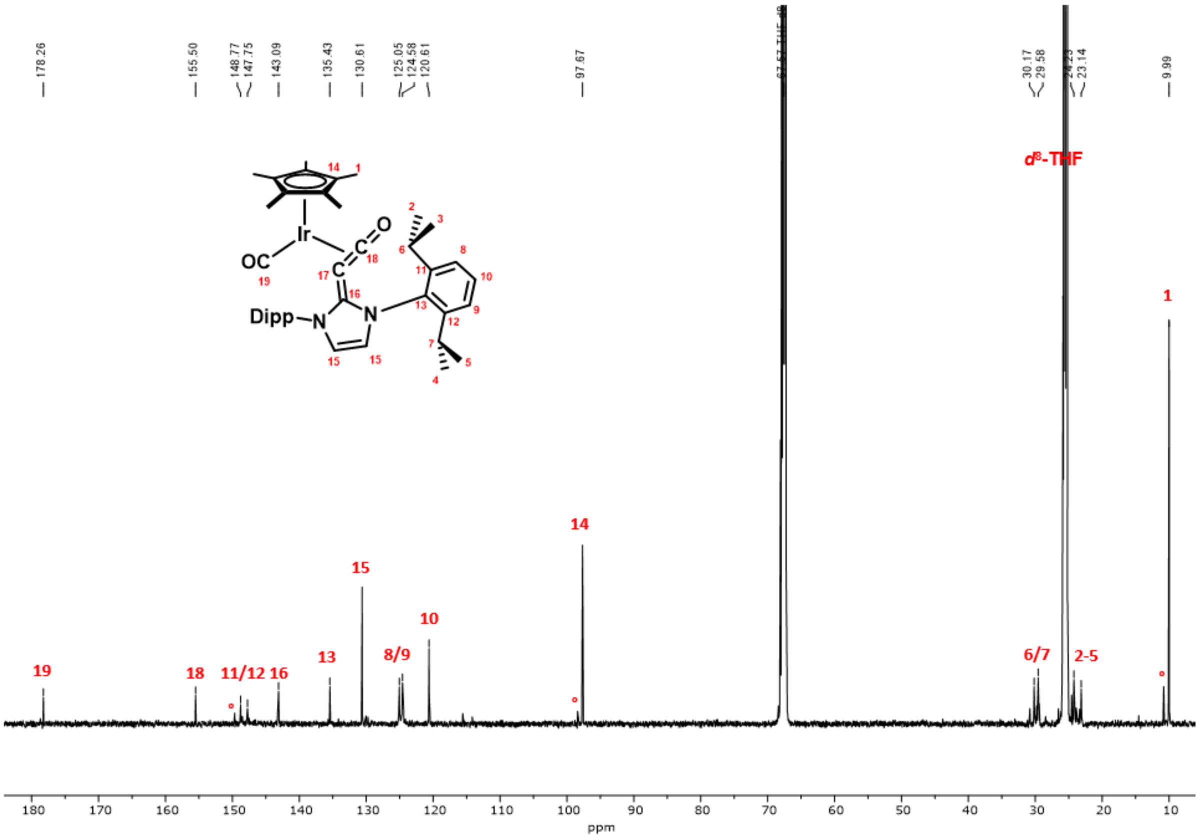

Figure S42 ^{13}C{^{1}H}-NMR spectrum (151 MHz, d_8-THF) of complex **11**. °: Cp*Ir(CO)$_2$.

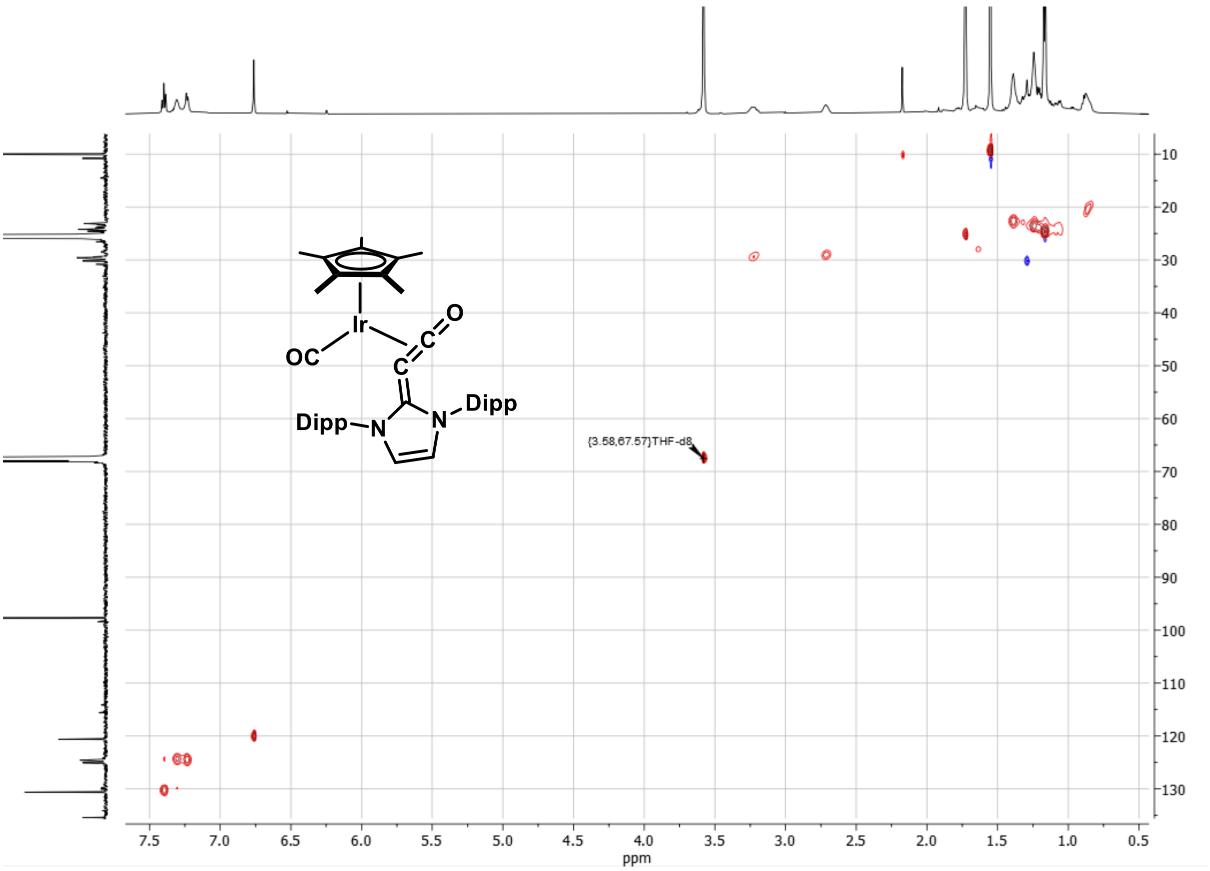

Figure S43 HSQC spectrum (151 MHz, d_8-THF) of complex **11**.

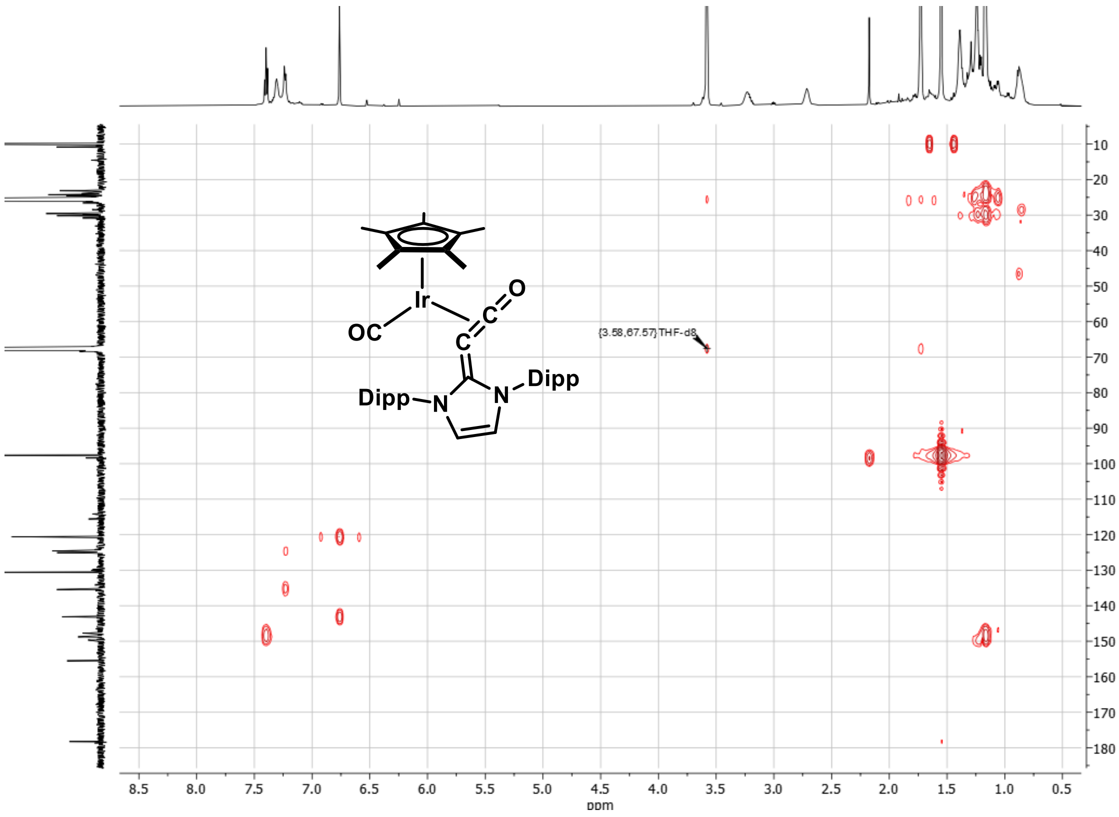

Figure S44 HSQC spectrum (151 MHz, d_8-THF) of complex **11**.

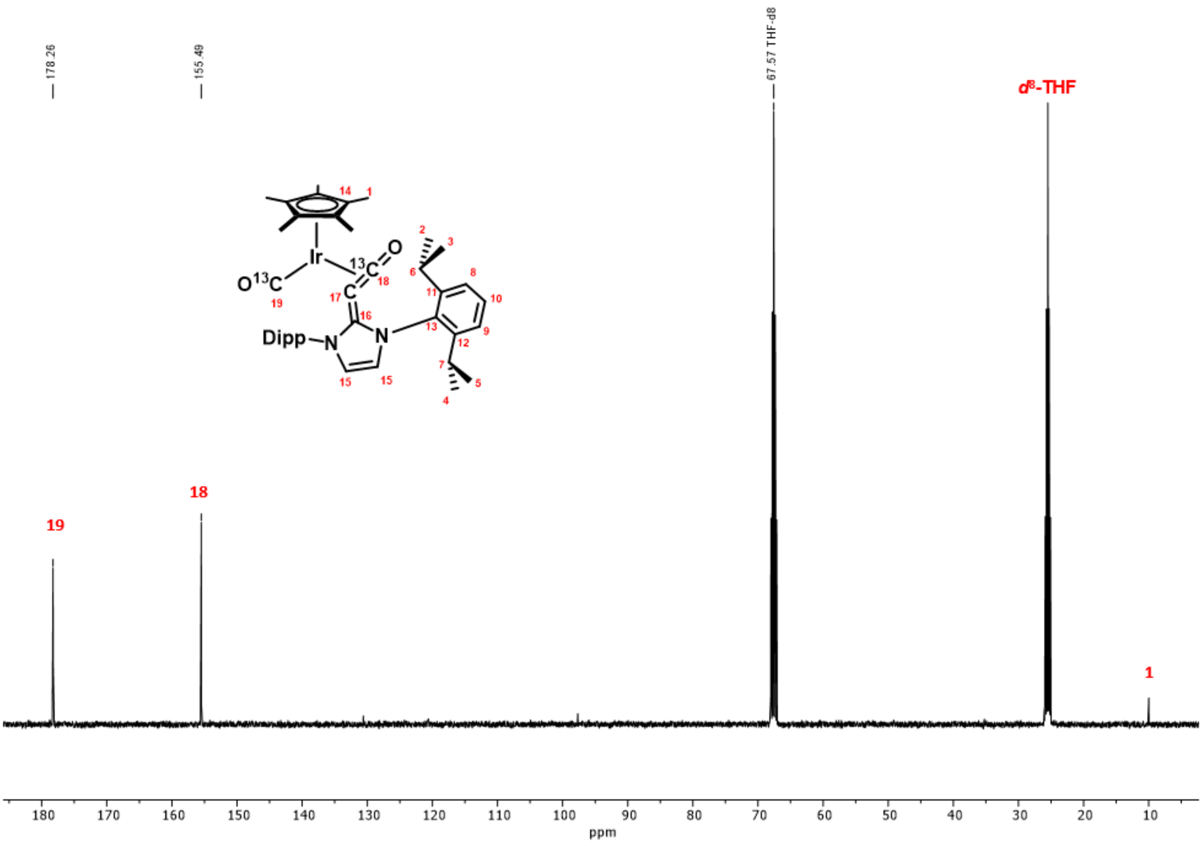

Figure S45 $^{13}C\{^1H\}$-NMR spectrum (151 MHz, d_8-THF) of complex **^{13}C-11**. The signal at 178.26 and 155.49 ppm indicated Ir-C̲O and NHC=C=C̲=O, respectively.

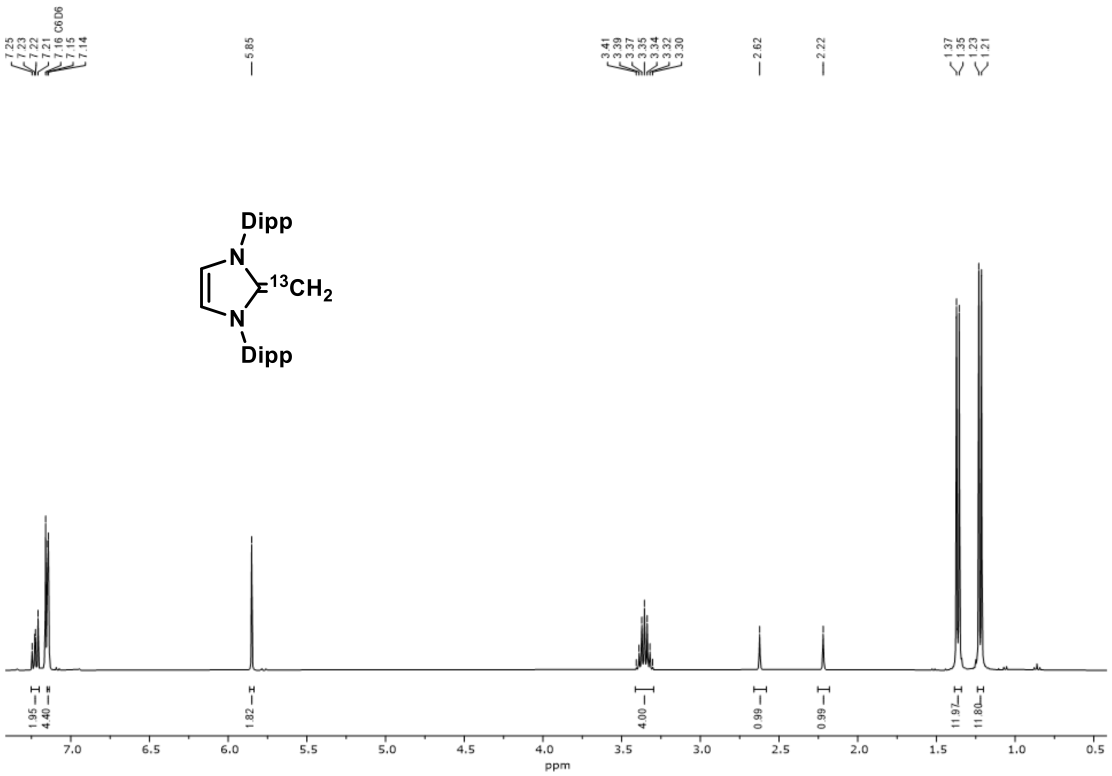

Figure S46 ^1H-NMR spectrum (400 MHz, C_6D_6) of IDipp$^{13}CH_2$.

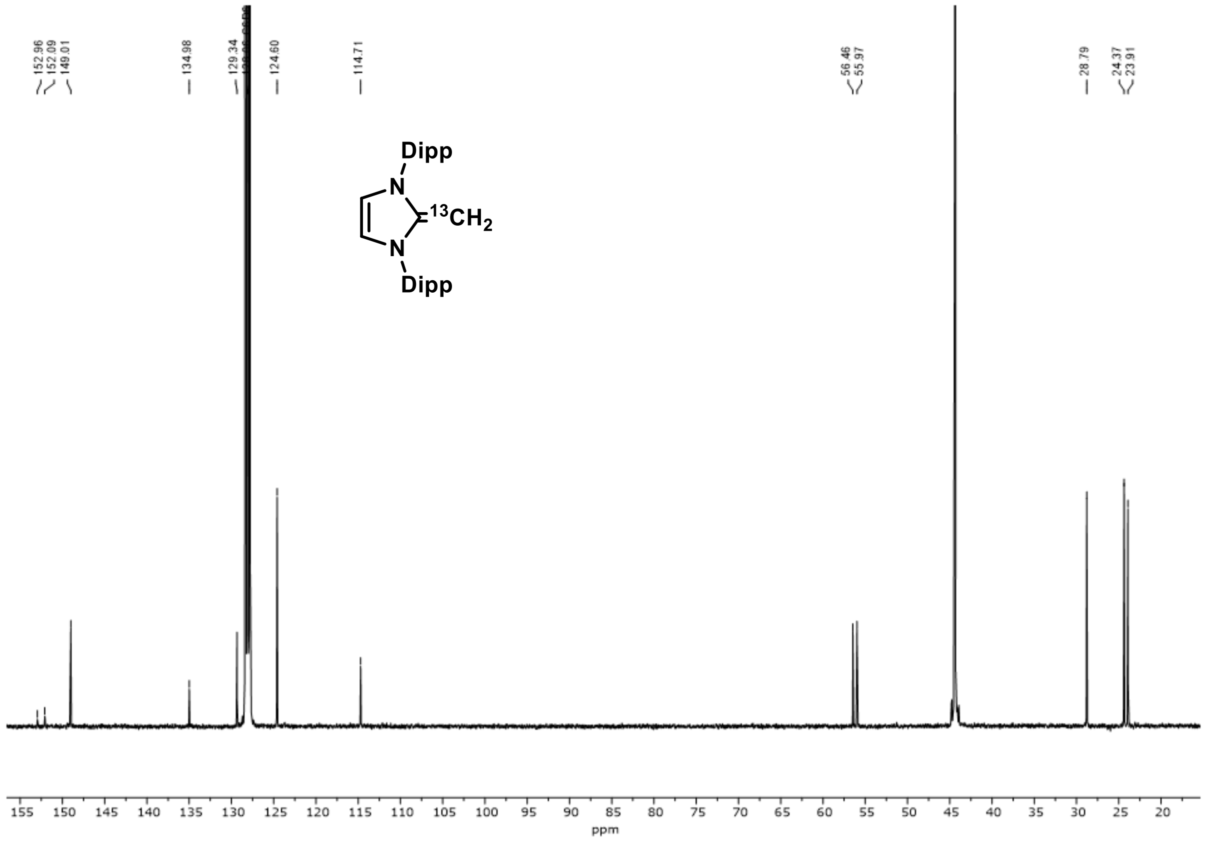

Figure S47 $^{13}C\{^1H\}$-NMR spectrum (101 MHz, C_6D_6) of IDipp$^{13}CH_2$.

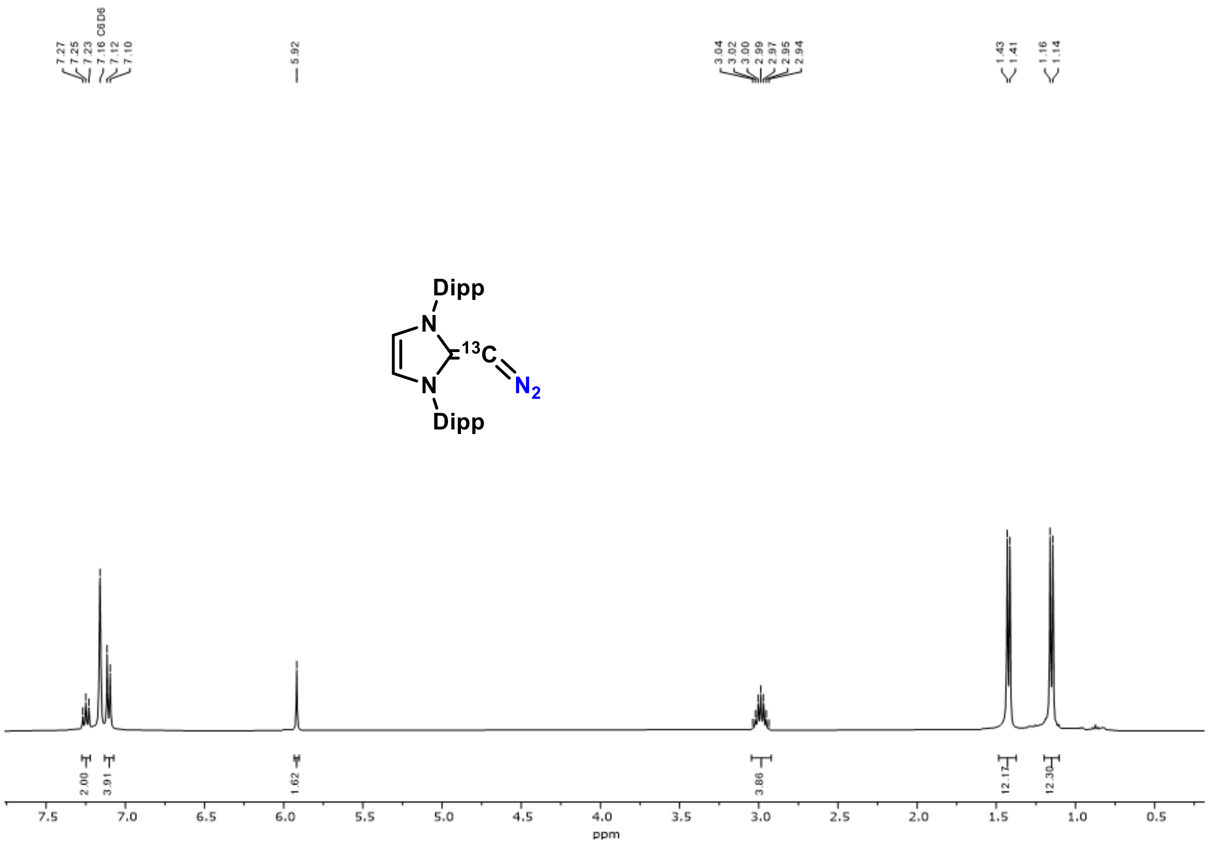

Figure S48 ^1H-NMR spectrum (400 MHz, C_6D_6) of compound **^{13}C-1**.

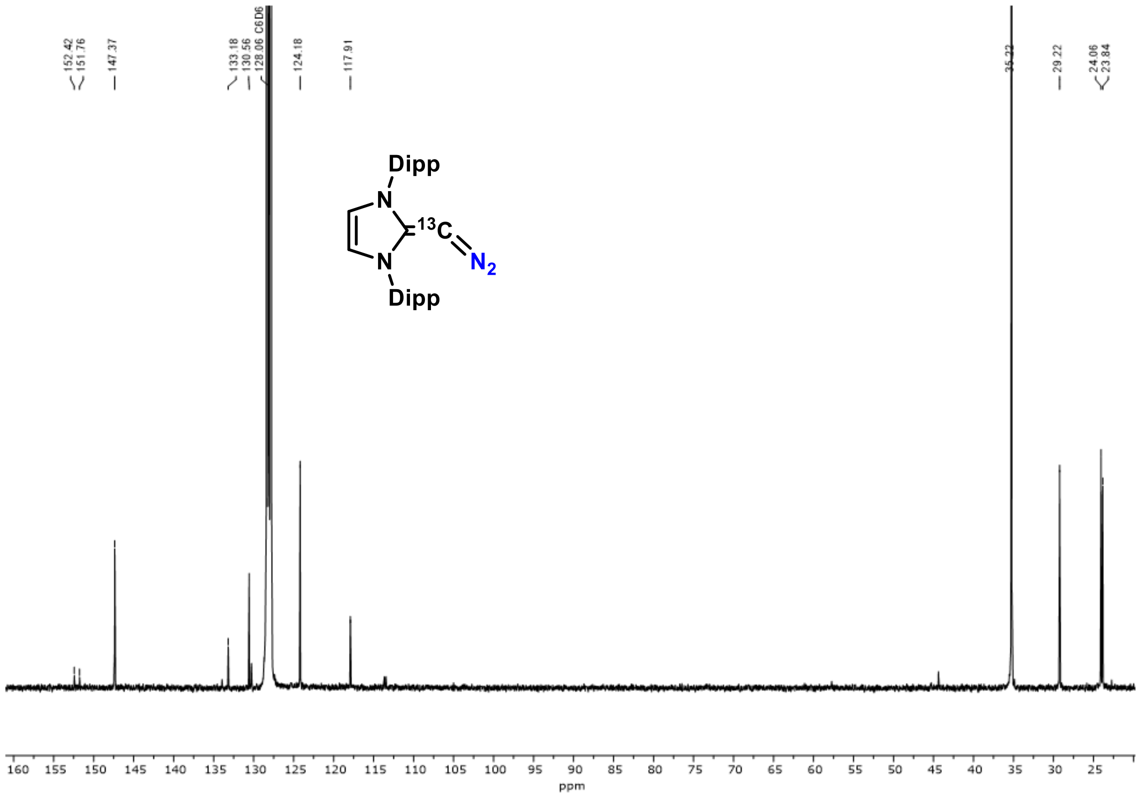

Figure S49 $^{13}C\{^{1}H\}$-NMR spectrum (101 MHz, C_6D_6) of compound **^{13}C-1**.

4. Additional NMR experiment

Reaction of 1 with [Cp*IrCl$_2$]$_2$: A solution of diazoolefin **1** (10.0 mg, 23 μmol) in d_8-toluene (0.25 mL) was added dropwise under stirring to a solution of [Cp*IrCl$_2$]$_2$ (9.3 mg, 12 μmol) in d_8-toluene (0.25 mL) at rt. The mixture was stirred at rt for 3 h. The mixture was transferred to a J-Young tube and the reaction was analyzed by NMR spectroscopy. The ^{1}H NMR spectrum indicates the presence of a mixture of compounds. Extraction with pentane did not give pure complex **2**.

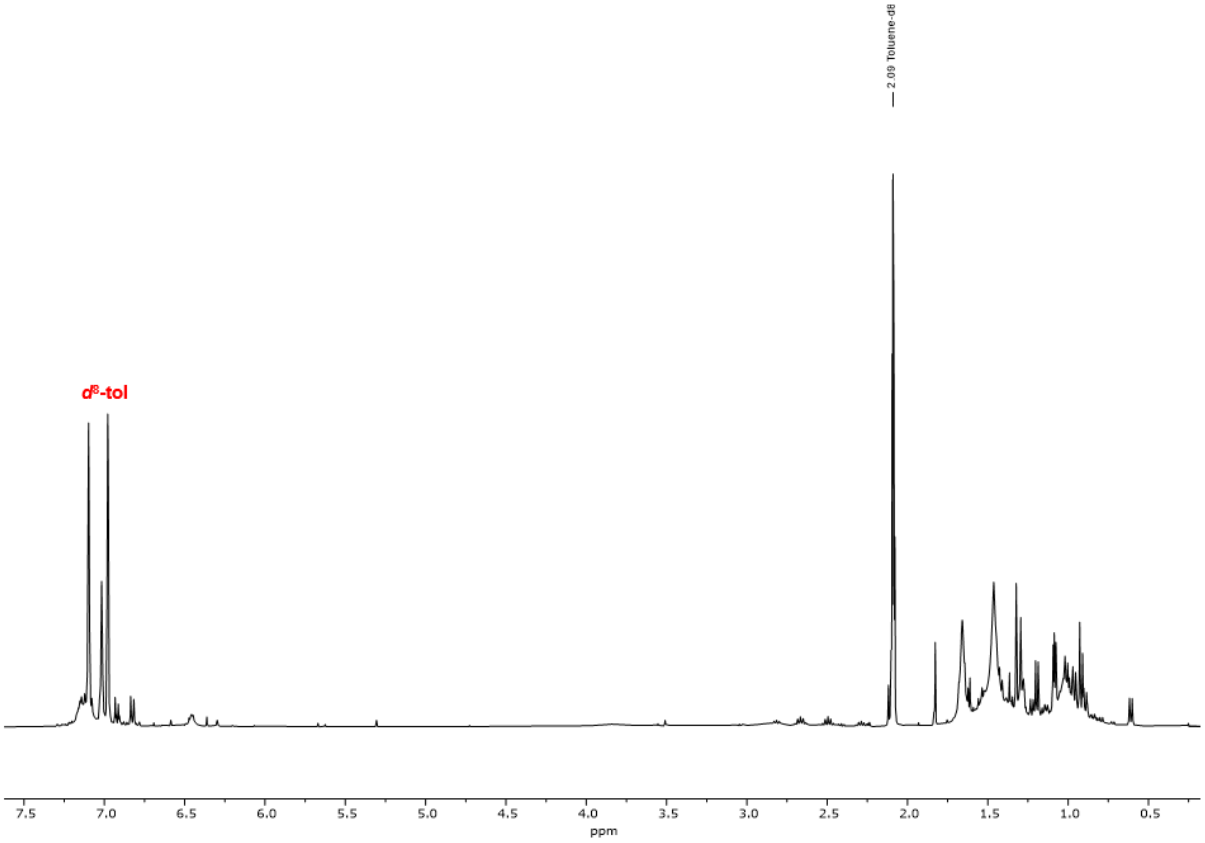

Figure S50 ^{1}H-NMR spectrum (400 MHz, d_8-toluene) of a solution containing diazoolefin **1** and [Cp*IrCl$_2$]$_2$ after a reaction time of 3 h at rt.

5. IR spectra

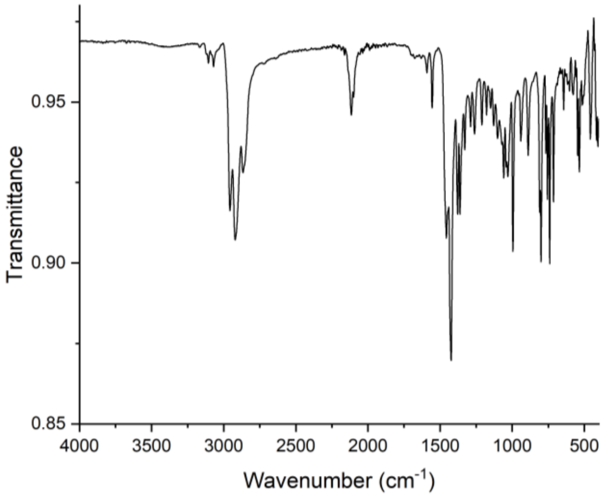

Figure S51 IR spectrum of complex **7**.

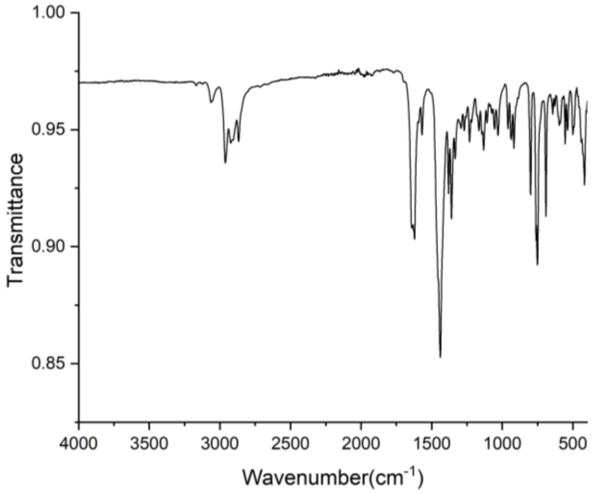

Figure S52 IR spectrum of complex **10**.

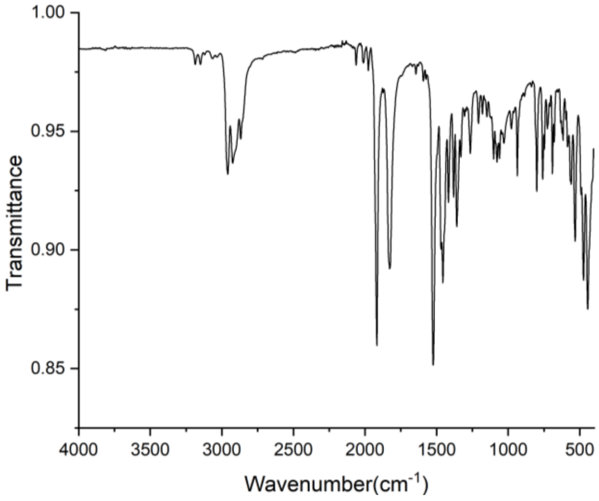

Figure S53 IR spectrum of complex **11**.

6. Single crystal X-ray analyses

<u>Complex **2**</u>

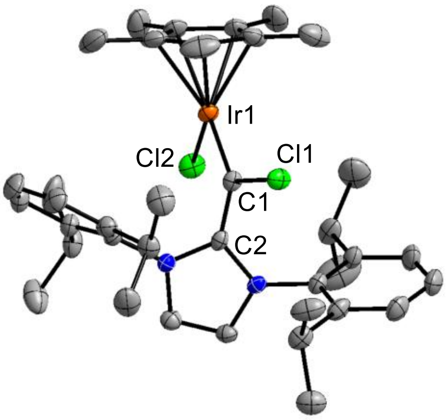

Figure S54 Molecular structure of complex **2** in the crystal. Thermal ellipsoids are at 50% probability and hydrogen atoms are not depicted. Selected bond lengths (Å): Ir1-C1 1.890(3), Ir1-Cl2 2.3818(12), C1-Cl1 1.815(3), C1-C2 1.448(5).

Structure Quality Indicators

Reflections:

Refinement:

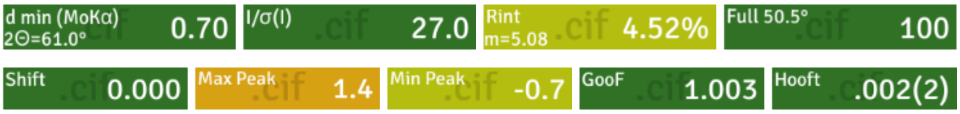

A metallic dark brown plate-shaped crystal with dimensions 0.22 × 0.15 × 0.03 mm^3 was mounted. Data were collected using an XtaLAB Synergy R, DW system, HyPix-Arc 150 diffractometer operating at T = 140.00(10) K. Data were measured using ω scans with Mo$K\alpha$ radiation. The diffraction pattern was indexed and the total number of runs and images was based on the strategy calculation from the program *CrysAlis*^{Pro} system (CCD 43.141a 64-bit (release 04-10-2024)).[3] The maximum resolution achieved was Θ = 30.51° (0.70 Å). The unit cell was refined using *CrysAlis*^{Pro} 1.171.43.141a[3] on 40138 reflections, 38% of the observed reflections. Data reduction, scaling and absorption corrections were performed using *CrysAlis*^{Pro} 1.171.43.141a[3]. The final completeness is 99.96 % out to 30.51° in Θ. A Gaussian absorption correction was performed using *CrysAlis*^{Pro} 1.171.43.141a[3] Numerical absorption correction based on Gaussian integration over a multifaceted crystal model. Empirical absorption correction using spherical harmonics as implemented in SCALE3 ABSPACK scaling algorithm. The absorption coefficient μ of this material is 3.862 mm^{-1} at this wavelength (λ = 0.71073 Å) and the minimum and maximum transmissions are 0.421

and 1.000. The structure was solved in the space group *Pca*2_1 (# 29) by the *ShelXT-2018/2*[4] structure solution program using dual methods and refined by full matrix least squares minimisation on $\boldsymbol{F^2}$ using version 2019/3 of *olex2.refine* 1.5[6], using *Olex2* 1.5[5] as the graphical interface. All non-hydrogen atoms were refined anisotropically. Hydrogen atom positions were calculated geometrically and refined using the riding model. The value of Z' is 2. This means that there are two independent molecules in the asymmetric unit. The moiety formula is $C_{38}H_{51}Cl_2IrN_2$. The Flack parameter was refined to 0.002(2). Determination of absolute structure using Bayesian statistics on Bijvoet differences using the *Olex2* results in None. This structure is in chiral space group, but there are no chiral atoms. Note: The Flack parameter is used to determine chirality of the crystal studied, the value should be near 0, a value of 1 means that the stereochemistry is wrong and the model should be inverted. A value of 0.5 means that the crystal consists of a racemic mixture of the two enantiomers.

Table S1. Crystal data and structure refinement for **2**.

Compound	DW450
Formula	$C_{38}H_{51}N_2Cl_2Ir$
$D_{calc.}$/ g cm^{-3}	1.465
μ/mm^{-1}	3.862
Formula Weight	798.963
Color	metallic dark brown
Shape	plate-shaped
Size/mm^3	0.22×0.15×0.03
T/K	140.00(10)
Crystal System	orthorhombic
Flack Parameter	0.002(2)
Space Group	$Pca2_1$
a/Å	23.9177(3)
b/Å	12.82363(12)
c/Å	23.6141(3)
α/°	90
β/°	90
γ/°	90
V/Å^3	7242.74(14)
Z	8
Z'	2
Wavelength/Å	0.71073
Radiation type	MoK_α
Θ_{in}/°	1.80
Θ_{max}/°	30.51
Measured Refl's.	106950
Indep't Refl's	21760
Refl's I≥2σ(I)	18546
R_{int}	0.0452
Parameters	852
Restraints	61
Largest Peak/e Å^{-3}	1.3796
Deepest Hole/e Å^{-3}	–0.6510
GooF	1.0029
wR_2 (all data)	0.0713
wR_2	0.0679
R_1 (all data)	0.0434
R_1	0.0318
CCDC number	2429379

Complex **3**

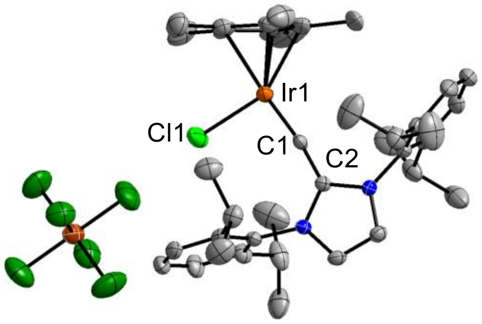

Figure S55 Molecular structure of complex **3** in the crystal. Thermal ellipsoids are at 50% probability, hydrogen atoms and solvents are not depicted. Selected bond lengths (Å): Ir1-C1 1.750(3), Ir1-Cl1 2.3065(9), C1-C2 1.411(4).

Structure Quality Indicators

**Reflections:**	d min (MoKα) 2Θ=61.0° 0.70	I/σ(I) 25.8	Rint m=6.16 5.91%	Full 50.5° 100
**Refinement:**	Shift -0.000	Max Peak 1.2	Min Peak -1.2	GooF 0.999

A metallic dark red prism-shaped crystal with dimensions 0.09 × 0.07 × 0.06 mm^3 was mounted. Data were collected using an XtaLAB Synergy R, DW system, HyPix-Arc 150 diffractometer operating at T = 140.00(10) K. Data were measured using ω scans with Mo$K\alpha$ radiation. The diffraction pattern was indexed and the total number of runs and images was based on the strategy calculation from the program *CrysAlis^{Pro}* system (CCD 43.137a 64-bit (release 10-09-2024)).[3] The maximum resolution achieved was Θ = 30.51° (0.70 Å). The unit cell was refined using *CrysAlis^{Pro}* 1.171.43.135a[3] on 25017 reflections, 32% of the observed reflections. Data reduction, scaling and absorption corrections were performed using *CrysAlis^{Pro}* 1.171.43.135a[3]. The final completeness is 99.99 % out to 30.51° in Θ. A Gaussian absorption correction was performed using *CrysAlis^{Pro}* 1.171.43.135a[3]. Numerical absorption correction based on Gaussian integration over a multifaceted crystal model. Empirical absorption correction using spherical harmonics as implemented in SCALE3 ABSPACK scaling algorithm. The absorption coefficient μ of this material is 3.816 mm^{-1} at this wavelength (λ = 0.71073 Å) and the minimum and maximum transmissions are 0.787 and 0.880. The structure was solved in the space group $P2_1/n$ (# 14) by the *ShelXT-2018/2*[4] structure solution program using iterative methods and refined by full matrix least squares minimisation on $\boldsymbol{F^2}$ using version 2019/3 of *olex2.refine* 1.5[6], using *Olex2*

1.5[5] as the graphical interface. All non-hydrogen atoms were refined anisotropically. Hydrogen atom positions were calculated geometrically and refined using the riding model. There is a single formula unit in the asymmetric unit, which is represented by the reported sum formula. In other words: Z is 4 and Z' is 1. The moiety formula is $C_{38}H_{51}ClIrN_2$, F_6Sb, C_4H_8O.

Table S2. Crystal data and structure refinement for **3**.

Compound	DW438
Formula	$C_{42}H_{59}N_2OF_6ClSbIr$
$D_{calc.}$/ g cm^{-3}	1.644
m/mm^{-1}	3.816
Formula Weight	1071.368
Color	metallic dark red
Shape	prism-shaped
Size/mm^3	0.09×0.07×0.06
T/K	140.00(10)
Crystal System	monoclinic
Space Group	$P2_1/n$
a/Å	12.1407(2)
b/Å	10.7553(2)
c/Å	33.3376(8)
α/°	90
β/°	96.0809(19)
γ/°	90
V/Å^3	4328.63(16)
Z	4
Z'	1
Wavelength/Å	0.71073
Radiation type	MoK_α
Θ_{min}/°	1.73
Θ_{max}/°	30.51
Measured Refl's.	79098
Indep't Refl's	13225
Refl's I≥2σ(I)	10624
R_{int}	0.0591
Parameters	550
Restraints	39
Largest Peak/e Å^{-3}	1.2266
Deepest Hole/e Å^{-3}	–1.2248
GooF	0.9986
wR_2 (all data)	0.0567
wR_2	0.0533
R_1 (all data)	0.0473
R_1	0.0316
CCDC number	2429378

Complex **4**

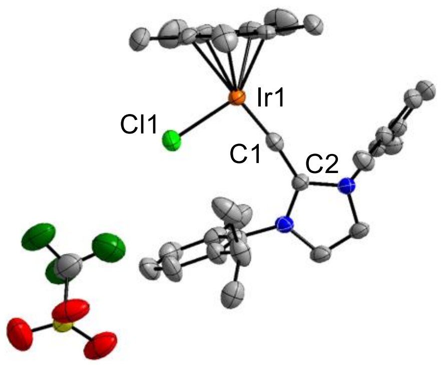

Figure S56 Molecular structure of complex **4** in the crystal. Thermal ellipsoids are at 50% probability. Solvents and hydrogen atoms are not depicted. Selected bond lengths (Å): Ir1-C1 1.731(4), Ir1-Cl1 2.3155(11), C1-C2 1.424(5).

Structure Quality Indicators

**Reflections:**	d min (CuKα) 2Θ=151.3° 0.80	I/σ(I) 40.5	Rint m=6.36 3.17%	Full 135.4° 98% to 151.3° 100
**Refinement:**	Shift 0.002	Max Peak 1.8	Min Peak -1.3	GooF 1.037

A clear dark purple plate-shaped crystal with dimensions 0.10 × 0.09 × 0.03 mm^3 was mounted. Data were collected using an XtaLAB Synergy R, DW system, HyPix-Arc 150 diffractometer operating at T = 140.00(10) K. Data were measured using ω scans with CuK_a radiation. The diffraction pattern was indexed and the total number of runs and images was based on the strategy calculation from the program *CrysAlis^{Pro}* system (CCD 44.105a 64-bit (release 14-04-2025)).[3] The maximum resolution achieved was Θ = 75.643° (0.80 Å). The unit cell was refined using *CrysAlis^{Pro}* 1.171.44.105a[3] on 17441 reflections, 32% of the observed reflections. Data reduction, scaling and absorption corrections were performed using *CrysAlis^{Pro}* 1.171.44.105a[3]. The final completeness is 100.00 % out to 75.643° in Θ. A Gaussian absorption correction was performed using *CrysAlis^{Pro}* 1.171.44.105a[3]. Numerical absorption correction based on Gaussian integration over a multifaceted crystal model. Empirical absorption correction using spherical harmonics as implemented in SCALE3 ABSPACK scaling algorithm. The absorption coefficient μ of this material is 7.436 mm^{-1} at this wavelength (λ = 1.54184 Å) and the minimum and maximum transmissions are 0.585 and 0.948. The structure was solved in the space group $P2_1/n$ (# 14) by the *ShelXT- 2018/2*[4] structure solution program using dual methods and

refined by full matrix least squares minimisation on $\boldsymbol{F^2}$ using version *ShelXL-2019/3*[7], using *Olex2* 1.5[5] as the graphical interface. All non-hydrogen atoms were refined anisotropically. Hydrogen atom positions were calculated geometrically and refined using the riding model. There is a single formula unit in the asymmetric unit, which is represented by the reported sum formula. In other words: Z is 4 and Z' is 1. The moiety formula is $C_{38}H_{51}ClIrN_2$, CF_3O_3S, C_4H_8O.

Table S3. Crystal data and structure refinement for **4**.

Compound	DW448b
Formula	$C_{43}H_{59}ClF_3IrN_2O_4S$
$D_{calc.}$/ g cm^{-3}	1.509
μ/mm^{-1}	7.436
Formula Weight	984.63
Color	Clear dark purple
Shape	plate-shaped
Size/mm^3	0.10×0.09×0.03
T/K	140.00(10)
Crystal System	monoclinic
Space Group	$P2_1/n$
a/Å	11.79059(14)
b/Å	10.82305(16)
c/Å	34.1364(4)
α/°	90
β/°	95.7406(11)
γ/°	90
V/Å^3	4334.30(10)
Z	4
Z'	1
Wavelength/Å	1.54184
Radiation type	Cu K_α
Θ_{min}/°	2.602
Θ_{max}/°	75.643
Measured Refl's.	53675
Indep't Refl's	8844
Refl's I≥2σ(I)	7684
R_{int}	0.0317
Parameters	692
Restraints	608
Largest Peak/e Å^{-3}	1.759
Deepest Hole/e Å^{-3}	–1.279
GooF	1.037
wR_2 (all data)	0.0985
wR_2	0.0949
R_1 (all data)	0.0420
R_1	0.0358
CCDC number	2429382

Complex **5**

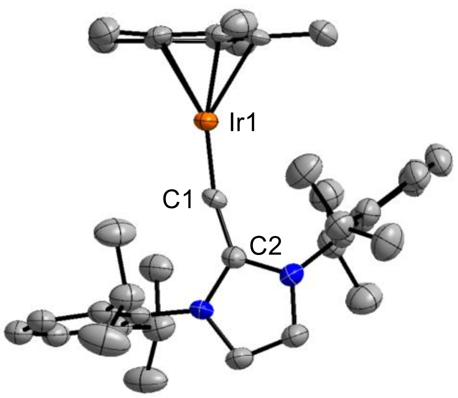

Figure S57 Molecular structure of complex **5** in the crystal. Thermal ellipsoids are at 50% probability and hydrogen atoms are not depicted. Selected bond lengths (Å) and angles (°): Ir1-C1 1.744(7), C1-C2 1.402(10), Ir1-C1-C2 167.0(6).

Structure Quality Indicators

**Reflections:**	d min (CuKα) 2Θ=149.0° 0.80	I/σ(I) 40.8	Rint m=1.84 n/a	Full 135.4° 92% to 149.0° 96.3
**Refinement:**	Shift 0.000	Max Peak 2.6	Min Peak -3.2	GooF 1.054

A colourless plate-shaped crystal with dimensions 0.10 × 0.04 × 0.01 mm^3 was mounted. Data were collected using an XtaLAB Synergy R, DW system, HyPix-Arc 150 diffractometer operating at T = 140.00(10) K. Data were measured using ω scans with Cu$K\alpha$ radiation. The diffraction pattern was indexed and the total number of runs and images was based on the strategy calculation from the program *CrysAlis*^{Pro} system (CCD 43.142a 64-bit (release 17-10-2024)).[3] The maximum resolution achieved was Θ = 74.492° (0.80 Å). The unit cell was refined using *CrysAlis*^{Pro} 1.171.43.141a[3] on 12851 reflections, 149% of the observed reflections. Data reduction, scaling and absorption corrections were performed using *CrysAlis*^{Pro} 1.171.43.141a[3]. The final completeness is 96.30 % out to 74.492° in Θ. A Gaussian absorption correction was performed using *CrysAlis*^{Pro} 1.171.43.141a[3]. Numerical absorption correction based on Gaussian integration over a multifaceted crystal model. Empirical absorption correction using spherical harmonics as implemented in SCALE3 ABSPACK scaling algorithm. The absorption coefficient μ of this material is 7.449 mm^{-1} at this wavelength (λ = 1.54184 Å) and the minimum and maximum transmissions are 0.578 and 1.000. The structure was solved in the space group $P\bar{1}$

(# 2) by the *ShelXT-2018/2*[4] structure solution program using dual methods and refined by full matrix least squares minimisation on $\boldsymbol{F^2}$ using *ShelXL-2019/3*[7], using *Olex2* 1.5[5] as the graphical interface. All non-hydrogen atoms were refined anisotropically. Hydrogen atom positions were calculated geometrically and refined using the riding model.

_refine_special_details: Refined as a 2-component twin.

_twin_special_details: Component 2 rotated by 1.9737° around [-0.55 -0.81 -0.22] (reciprocal) or [-0.30 -0.95 0.01] (direct)

There is a single formula unit in the asymmetric unit, which is represented by the reported sum formula. In other words: Z is 2 and Z' is 1. The moiety formula is $C_{38}H_{51}IrN_2$.

Table S4. Crystal data and structure refinement for **5**.

Compound	DW462
Formula	$C_{38}H_{51}IrN_2$
$D_{calc.}$/ g cm^{-3}	1.360
m/mm^{-1}	7.449
Formula Weight	728.00
Color	colorless
Shape	plate-shaped
Size/mm^3	0.10×0.04×0.01
T/K	140.00(10)
Crystal System	triclinic
Space Group	$P\bar{1}$
a/Å	10.3752(2)
b/Å	10.5393(2)
c/Å	19.0044(4)
α/°	82.6741(18)
β/°	79.4022(19)
γ/°	60.562(2)
V/Å^3	1777.22(8)
Z	2
Z'	1
Wavelength/Å	1.54184
Radiation type	CuK_α
Θ_{min}/°	2.367
Θ_{max}/°	74.492
Measured Refl's.	8640
Indep't Refl's	8640
Refl's I≥2σ(I)	7829
R_{int}	n/a
Parameters	384
Restraints	465
Largest Peak/e Å^{-3}	2.573
Deepest Hole/e Å^{-3}	–3.200
GooF	1.054
wR_2 (all data)	0.1623
wR_2	0.1575
R_1 (all data)	0.0655
R_1	0.0600
CCDC number	2429377

Complex **6**

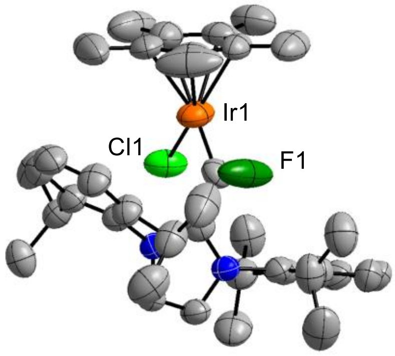

Figure S58 Molecular structure of complex **6** in the crystal. Thermal ellipsoids are at 50% probability, hydrogen atoms and solvents are not depicted.

Structure Quality Indicators

**Reflections:**	d min (CuKα) 2Θ=149.4° 0.80	I/σ(I) 22.2	Rint m=4.46 5.15%	Full 135.4° 95% to 149.4° 100
**Refinement:**	Shift 0.001	Max Peak 1.2	Min Peak -0.7	GooF 0.972

A clear dark brown plate-shaped crystal with dimensions 0.14 × 0.08 × 0.04 mm^3 was mounted. Data were collected using a XtaLAB Synergy R, DW system, HyPix-Arc 150 diffractometer operating at T = 139.99(10) K. Data were measured using ω scans with CuK_α radiation. The diffraction pattern was indexed and the total number of runs and images was based on the strategy calculation from the program *CrysAlis^{Pro}* system (CCD 44.91a 64-bit (release 23-01-2025)).[3] The maximum resolution that was achieved was Θ = 74.69° (0.80 Å). The unit cell was refined using *CrysAlis^{Pro}* 1.171.44.92a on 9175 reflections, 26% of the observed reflections. Data reduction, scaling and absorption corrections were performed using *CrysAlis^{Pro}* 1.171.44.92a[3]. The final completeness is 99.96 % out to 74.69° in Θ. An analytical absorption correction was performed using *CrysAlis^{Pro}* 1.171.44.92a[3]. The analytical numeric absorption correction was done using a multifaceted crystal model based on expressions derived by R.C. Clark & J.S. Reid[8]. The empirical absorption correction was done using spherical harmonics, implemented in SCALE3 ABSPACK scaling algorithm. The absorption coefficient μ of this crystal is 7.055 mm^{-1} at this wavelength (λ = 1.54184 Å) and the minimum and maximum transmissions are 0.565 and 0.832. The structure was solved and the space group $P2_1/c$ (# 14) determined by the *ShelXT*[4] structure solution program using dual methods and refined by full matrix least squares

minimisation on $\boldsymbol{F^2}$ using version of *olex2.refine* 1.5[6], using *Olex2* 1.5[5] as the graphical interface. All non-hydrogen atoms were refined anisotropically. Hydrogen atom positions were calculated geometrically and refined using the riding model.

_smtbx_masks_special_details: A solvent mask was calculated and 120 electrons were found in a volume of 980 Å^3 in 1 void per unit cell. This is consistent with the presence of 0.75 [C_5H_{12}] per Asymmetric Unit which account for 126 electrons per unit cell.

There is a single formula unit in the asymmetric unit, which is represented by the reported sum formula. In other words: Z is 4 and Z' is 1. The moiety formula is $C_{38}H_{51}ClFIrN_2$, 0.75[C_5H_{12}].

Table S5. Crystal data and structure refinement for **6**.

Compound	DW548
Formula	$C_{41.75}H_{60}ClFIrN_2$
$D_{calc.}$/ g cm^{-3}	1.338
μ/mm^{-1}	7.055
Formula Weight	836.622
Color	clear dark brown
Shape	plate
Size/mm^3	0.14×0.08×0.04
T/K	139.99(10)
Crystal System	monoclinic
Space Group	$P2_1/c$
a/Å	12.5530(3)
b/Å	13.8669(4)
c/Å	24.6094(8)
α/°	90
β/°	104.119(3)
γ/°	90
V/Å^3	4154.4(2)
Z	4
Z'	1
Wavelength/Å	1.54184
Radiation type	CuK_α
Θ_{min}/°	3.63
Θ_{max}/°	74.69
Measured Refl's.	35301
Indep't Refl's	8130
Refl's I≥2 σ(I)	5728
R_{int}	0.0515
Parameters	426
Restraints	0
Largest Peak	1.2109
Deepest Hole	–0.6774
GooF	0.9718
wR_2 (all data)	0.1243
wR_2	0.1138
R_1 (all data)	0.0682
R_1	0.0450
CCDC number	2425614

Complex **7**

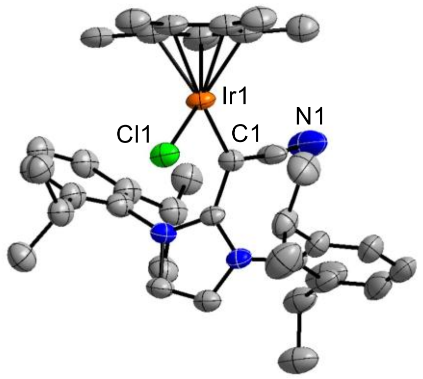

Figure S59 Molecular structure of complex **7** in the crystal. Thermal ellipsoids are at 50% probability and hydrogen atoms are not depicted.

Structure Quality Indicators

**Reflections:**	d min (CuKα) 2Θ=150.0° 0.80	I/σ(I) 13.8	Rint m=4.91 6.85%	Full 135.4° 97% to 150.0° 100
**Refinement:**	Shift 0.000	Max Peak 1.8	Min Peak -1.1	GooF 1.029

A clear intense green plate-shaped crystal with dimensions 0.16 × 0.05 × 0.04 mm^3 was mounted. Data were collected using a XtaLAB Synergy R, DW system, HyPix-Arc 150 diffractometer operating at T = 140.00(10) K. Data were measured using ω scans with CuK_α radiation. The diffraction pattern was indexed and the total number of runs and images was based on the strategy calculation from the program *CrysAlis^{Pro}* system (CCD 44.104a 64-bit (release 03-04-2025)).[3] The maximum resolution that was achieved was Θ = 75.011° (0.80 Å). The unit cell was refined using *CrysAlis^{Pro}* 1.171.44.104a[3] on 10802 reflections, 17% of the observed reflections. Data reduction, scaling and absorption corrections were performed using *CrysAlis^{Pro}* 1.171.44.104a[3]. The final completeness is 100.00 % out to 75.011° in Θ. An analytical absorption correction was performed using *CrysAlis^{Pro}* 1.171.44.104a[3]. The analytical numeric absorption correction was done using a multifaceted crystal model based on expressions derived by R.C. Clark & J.S. Reid[8]. The empirical absorption correction was done using spherical harmonics, implemented in SCALE3 ABSPACK scaling algorithm. The absorption coefficient μ of this crystal is 7.969 mm^{-1} at this wavelength (λ = 1.54184 Å) and the minimum and maximum transmissions are 0.453 and 0.780. The structure was solved and the space group $Pca2_1$ (# 29) determined by the *ShelXT*[4]

structure solution program using dual methods and refined by full matrix least squares minimisation on $\boldsymbol{F^2}$ using *ShelXL-2019/3*[7], using *Olex2* 1.5[5] as the graphical interface. All non-hydrogen atoms were refined anisotropically. Hydrogen atom positions were calculated geometrically and refined using the riding model.

_refine_special_details: Refined as a 2-component inversion twin.

The value of Z' is 2. This means that there are two independent molecules in the asymmetric unit. The moiety formula is 1($C_{39}H_{51}ClIrN_3$). The Flack parameter was refined to 0.024(19). Determination of absolute structure using Bayesian statistics on Bijvoet differences using the *Olex2* results in -0.005(5). The chiral atoms in this structure are: N2(R), C42(S), C43(R), C44(R), C45(R), C46(S), C53(R), C54(R), C55(S), C56(S), C91(S), C92(R), C93(R), C94(R), C95(S), C101(R), C102(R), C103(R), C104(S), C105(S). Note: The Flack parameter is used to determine chirality of the crystal studied, the value should be near 0, a value of 1 means that the stereochemistry is wrong and the model should be inverted. A value of 0.5 means that the crystal consists of a racemic mixture of the two enantiomers.

Table S6. Crystal data and structure refinement for **7.**

Compound	DW539b
Formula	$C_{39}H_{51}ClIrN_3$
$D_{calc.}$/ g cm^{-3}	1.437
μ/mm^{-1}	7.969
Formula Weight	789.47
Color	clear intense green
Shape	plate
Size/mm^3	0.16×0.05×0.04
T/K	140.00(10)
Crystal System	orthorhombic
Flack Parameter	0.024(19)
Hooft Parameter	–0.005(5)
Space Group	$Pca2_1$
a/Å	24.3343(5)
b/Å	12.9026(2)
c/Å	23.2450(4)
α/°	90
β/°	90
γ/°	90
V/Å^3	7298.4(2)
Z	8
Z'	2
Wavelength/Å	1.54184
Radiation type	CuK_α
Θ_{min}/°	3.425
Θ_{max}/°	75.011
Measured Refl's.	62924
Indep't Refl's	13357
Refl's I≥2 σ(I)	8662
R_{int}	0.0685
Parameters	1125
Restraints	2163
Largest Peak	1.777
Deepest Hole	–1.150
GooF	1.029
wR_2 (all data)	0.1324
wR_2	0.1151
R_1 (all data)	0.1000
R_1	0.0537
CCDC number	2425615

Complex **8**

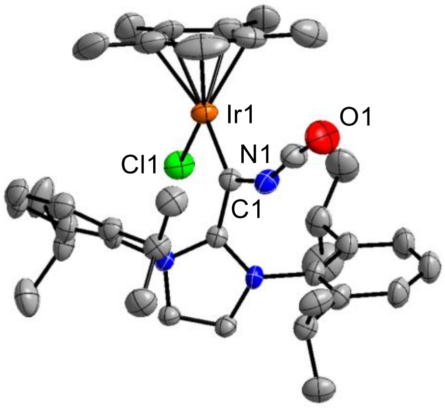

Figure S60 Molecular structure of complex **8** in the crystal. Thermal ellipsoids are at 50% probability and hydrogen atoms are not depicted.

Structure Quality Indicators

**Reflections:**	d min (CuKα) 2Θ=149.4° 0.80	I/σ(I) 34.4	Rint m=5.28 3.55%	Full 135.4° 98% to 149.4° 100
**Refinement:**	Shift 0.001	Max Peak 1.8	Min Peak -1.1	GooF 1.046

A metallic dark green plate-shaped crystal with dimensions 0.13 × 0.09 × 0.02 mm^3 was mounted. Data were collected using an XtaLAB Synergy R, DW system, HyPix-Arc 150 diffractometer operating at T = 140.00(10) K. Data were measured using ω scans with CuK_α radiation. The diffraction pattern was indexed and the total number of runs and images was based on the strategy calculation from the program *CrysAlis*^{Pro} system (CCD 44.88a 64-bit (release 09-01-2025)).[3] The maximum resolution achieved was Θ = 74.676° (0.80 Å). The unit cell was refined using *CrysAlis*^{Pro} 1.171.44.88a[3] on 24056 reflections, 33% of the observed reflections. Data reduction, scaling and absorption corrections were performed using *CrysAlis*^{Pro} 1.171.44.88a[3]. The final completeness is 100.00 % out to 74.676° in Θ. A Gaussian absorption correction was performed using *CrysAlis*^{Pro} 1.171.44.88a[3]. Numerical absorption correction based on Gaussian integration over a multifaceted crystal model. Empirical absorption correction using spherical harmonics as implemented in SCALE3 ABSPACK scaling algorithm. The absorption coefficient μ of this material is 7.804 mm^{-1} at this wavelength (λ = 1.54184 Å) and the minimum and maximum transmissions are 0.421 and 1.000. The structure was solved in the space group $Pca2_1$ (# 29) by the *ShelXT-2018/2*[4] structure solution program using dual methods and refined by full matrix least

squares minimisation on $\boldsymbol{F^2}$ using *ShelXL-2019/3*[7], using *Olex2* 1.5[5] as the graphical interface. All non-hydrogen atoms were refined anisotropically. Hydrogen atom positions were calculated geometrically and refined using the riding model.

_refine_special_details: Refined as a 2-component inversion twin.

The value of Z' is 2. This means that there are two independent molecules in the asymmetric unit. The moiety formula is $C_{39}H_{51}ClIrN_3O$. The Flack parameter was refined to 0.407(10). Determination of absolute structure using Bayesian statistics on Bijvoet differences using the *Olex2* results in None. This structure is in chiral space group, but there are no chiral atoms. Note: The Flack parameter is used to determine chirality of the crystal studied, the value should be near 0, a value of 1 means that the stereochemistry is wrong and the model should be inverted. A value of 0.5 means that the crystal consists of a racemic mixture of the two enantiomers.

Table S7. Crystal data and structure refinement for **8**.

Compound	DW527
Formula	$C_{39}H_{51}ClIrN_3O$
$D_{calc.}$/ g cm^{-3}	1.430
m/mm^{-1}	7.804
Formula Weight	805.47
Color	metallic dark green
Shape	plate-shaped
Size/mm^3	0.13×0.09×0.02
T/K	140.00(10)
Crystal System	orthorhombic
Flack Parameter	0.407(10)
Space Group	$Pca2_1$
a/Å	25.04721(18)
b/Å	12.97420(11)
c/Å	23.0278(2)
α/°	90
β/°	90
γ/°	90
V/Å^3	7483.28(11)
Z	8
Z'	2
Wavelength/Å	1.54184
Radiation type	CuK_α
Θ_{min}/°	3.406
Θ_{max}/°	74.676
Measured Refl's.	72371
Indep't Refl's	14387
Refl's I≥2σ(I)	12962
R_{int}	0.0355
Parameters	848
Restraints	1369
Largest Peak/e Å^{-3}	1.795
Deepest Hole/e Å^{-3}	–1.094
GooF	1.046
wR_2 (all data)	0.0909
wR_2	0.0885
R_1 (all data)	0.0405
R_1	0.0354
CCDC number	2429381

Complex **9**

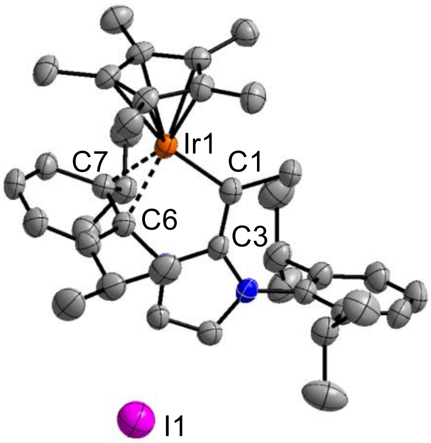

Figure S61 Molecular structure of complex **9** in the crystal. Thermal ellipsoids are at 50% probability and hydrogen atoms are not depicted. Selected bond lengths (Å): Ir1-C1 1.942(5), Ir1-C6 2.150(5), Ir1-C7 2.242(5), C1-C3 1.435(7).

Structure Quality Indicators

**Reflections:**	d min (CuKα) 2Θ=151.2° 0.80	I/σ(I) 33.2	Rint m=5.67 4.74%	Full 135.4° 98% to 151.2° 99.8
**Refinement:**	Shift 0.004	Max Peak 2.3	Min Peak -2.1	GooF 1.091

A clear dark red plate-shaped crystal with dimensions 0.24 × 0.16 × 0.10 mm^3 was mounted. Data were collected using a XtaLAB Synergy R, DW system, HyPix-Arc 150 diffractometer operating at T = 140.00(10) K. Data were measured using *w* scans with CuK_α radiation. The diffraction pattern was indexed and the total number of runs and images was based on the strategy calculation from the program *CrysAlis^{Pro}* system (CCD 44.91a 64-bit (release 23-01-2025)).[3] The maximum resolution that was achieved was Θ = 75.584° (0.80 Å). The unit cell was refined using *CrysAlis*^{Pro} 1.171.44.92a[3] on 16187 reflections, 39% of the observed reflections. Data reduction, scaling and absorption corrections were performed using *CrysAlis*^{Pro} 1.171.44.92a[3]. The final completeness is 99.80 % out to 75.584° in Θ. An analytical absorption correction was performed using *CrysAlis*^{Pro} 1.171.44.92a[3]. The analytical numeric absorption correction was done using a multifaceted crystal model based on expressions derived by R.C. Clark & J.S. Reid.[8] The empirical absorption correction using spherical harmonics, implemented in SCALE3 ABSPACK scaling algorithm. The absorption coefficient μ of this material is 13.691 mm^{-1} at this wavelength (λ = 1.54184

Å) and the minimum and maximum transmissions are 0.109 and 0.409. The structure was solved and the space group *C*2/*c* (# 15) determined by the *ShelXT*[4] structure solution program using dual methods and refined by full matrix least squares minimisation on $\boldsymbol{F^2}$ using *ShelXL-2019/3*[7], using *Olex2* 1.5[5] as the graphical interface. All non-hydrogen atoms were refined anisotropically. Hydrogen atom positions were calculated geometrically and refined using the riding model. There is a single formula unit in the asymmetric unit, which is represented by the reported sum formula. In other words: Z is 8 and Z' is 1. The moiety formula is $C_{39}H_{54}IrN_2$, I.

Table S8. Crystal data and structure refinement for **9**.

Compound	dw551
Formula	$C_{39}H_{54}IIrN_2$
$D_{calc.}$/ g cm^{-3}	1.557
μ/mm^{-1}	13.691
Formula Weight	869.94
Color	clear dark red
Shape	plate
Size/mm^3	0.24×0.16×0.10
T/K	140.00(10)
Crystal System	monoclinic
Space Group	*C*2/*c*
a/Å	34.3815(4)
b/Å	13.37884(14)
c/Å	16.57256(15)
α/°	90
β/°	103.2518(10)
γ/°	90
V/Å^3	7420.13(14)
Z	8
Z'	1
Wavelength/Å	1.54184
Radiation type	CuK_α
Θ_{min}/°	2.641
Θ_{max}/°	75.584
Measured Refl's.	41331
Indep't Refl's	7564
Refl's I≥2 σ(I)	6634
R_{int}	0.0474
Parameters	402
Restraints	0
Largest Peak	2.314
Deepest Hole	–2.083
GooF	1.091
wR_2 (all data)	0.1260
wR_2	0.1219
R_1 (all data)	0.0496
R_1	0.0440
CCDC number	2425613

Complex **10**

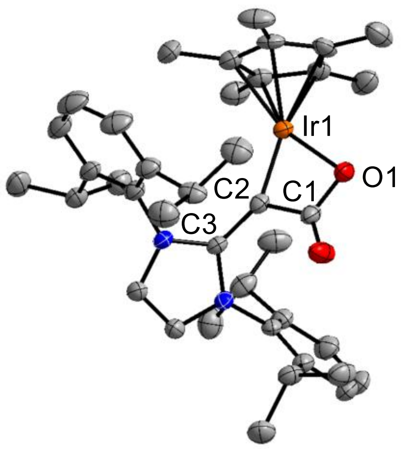

Figure S62 Molecular structure of complex **10** in the crystal. Thermal ellipsoids are at 50% probability and hydrogen atoms are not depicted. Selected bond lengths (Å): Ir1-O1 2.091(2), Ir1-C2 1.946(3), O1-C1 1.320(4), C1-C2 1.473(4), C2-C3 1.436(4).

Structure Quality Indicators

**Reflections:**	d min (CuKα) 2Θ=151.2° 0.80	I/σ(I) 40.0	Rint m=5.37 3.25%	Full 135.4° 99% to 151.2° 100
**Refinement:**	Shift -0.003	Max Peak 1.2	Min Peak -1.6	GooF 1.052

A clear dark brown irregular-shaped crystal with dimensions 0.16 × 0.10 × 0.05 mm^3 was mounted. Data were collected using a XtaLAB Synergy R, DW system, HyPix-Arc 150 diffractometer operating at T = 140.00(10) K. Data were measured using w scans with CuK_α radiation. The diffraction pattern was indexed and the total number of runs and images was based on the strategy calculation from the program *CrysAlis^{Pro}* system (CCD 44.91a 64-bit (release 23-01-2025))[3]. The maximum resolution that was achieved was Θ = 75.595° (0.80 Å). The unit cell was refined using *CrysAlis^{Pro}* 1.171.44.92a[3] on 18114 reflections, 48% of the observed reflections. Data reduction, scaling and absorption corrections were performed using *CrysAlis^{Pro}* 1.171.44.92a[3]. The final completeness is 100.00 % out to 75.595° in Θ. An analytical absorption correction was performed using *CrysAlis^{Pro}* 1.171.44.92a[3]. The analytical numeric absorption correction was done using a multifaceted crystal model based on expressions derived by R.C. Clark & J.S. Reid.[8] The empirical absorption correction was carried out using spherical harmonics, implemented in SCALE3 ABSPACK scaling algorithm. The absorption coefficient μ of this crystal is 7.650 mm^{-1} at this

wavelength (λ = 1.54184Å) and the minimum and maximum transmissions are 0.467 and 0.765. The structure was solved and the space group $P2_1/c$ (# 14) determined by the *ShelXT*[4] structure solution program using dual methods and refined by full matrix least squares minimisation on $\boldsymbol{F^2}$ using *ShelXL-2019/3*[7], using *Olex2* 1.5[5] as the graphical interface. All non-hydrogen atoms were refined anisotropically. Hydrogen atom positions were calculated geometrically and refined using the riding model. There is a single formula unit in the asymmetric unit, which is represented by the reported sum formula. In other words: Z is 4 and Z' is 1. The moiety formula is $C_{39}H_{51}IrN_2O_2$.

Table S9. Crystal data and structure refinement for **10**.

Compound	dw556
Formula	$C_{39}H_{51}N_2O_2Ir$
$D_{calc.}$/ g cm^{-3}	1.466
μ/mm^{-1}	7.650
Formula Weight	772.02
Color	clear dark brown
Shape	irregular
Size/mm^3	0.16×0.10×0.05
T/K	140.00(10)
Crystal System	monoclinic
Space Group	$P2_1/c$
a/Å	14.70356(12)
b/Å	14.13875(13)
c/Å	16.88387(15)
α/°	90
β/°	94.8079(7)
γ/°	90
V/Å^3	3497.64(5)
Z	4
Z'	1
Wavelength/Å	1.54184
Radiation type	Cu K_α
Θ_{min}/°	3.016
Θ_{max}/°	75.595
Measured Refl's.	37389
Indep't Refl's	7178
Refl's I ≥ 2σ(I)	6345
R_{int}	0.0325
Parameters	411
Restraints	0
Largest Peak	1.177
Deepest Hole	–1.635
GooF	1.052
wR_2 (all data)	0.0781
wR_2	0.0749
R_1 (all data)	0.0331
R_1	0.0282
CCDC number	2425611

Complex **11**

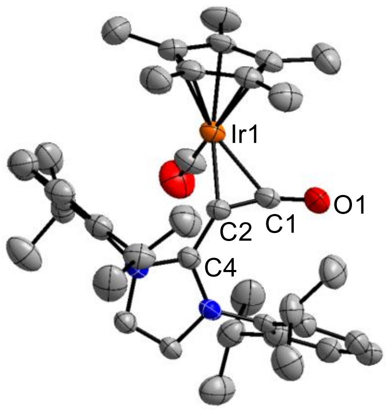

Figure S63 Molecular structure of complex **11** in the crystal. Thermal ellipsoids are at 50% probability and hydrogen atoms are not depicted. Selected bond lengths (Å): Ir1-C1 2.142(10), Ir1-C2 2.096(9), O1-C1 1.224(12), C1-C2 1.302(13), C2-C4 1.402(12).

Structure Quality Indicators

**Reflections:**	d min (CuKα) 2Θ=133.2° 0.84	I/σ(I) 8.9	Rint m=2.87 9.46%	Full 133.2° 99.9
**Refinement:**	Shift 0.001	Max Peak 1.8	Min Peak -1.2	GooF 1.023

A clear intense yellow plate-shaped crystal with dimensions 0.13 × 0.04 × 0.04 mm^3 was mounted. Data were collected using a SuperNova, Dual, Cu at home/near, AtlasS2 diffractometer operating at T = 139.99(10) K. Data were measured using ω scans with CuK_α radiation. The diffraction pattern was indexed and the total number of runs and images was based on the strategy calculation from the program *CrysAlis^{Pro}* system (CCD 44.92a 64-bit (release 31-01-2025)).[3] The maximum resolution that was achieved was Θ = 66.595° (0.84 Å). The unit cell was refined using *CrysAlis^{Pro}* 1.171.44.92a[3] on 4196 reflections, 23% of the observed reflections. Data reduction, scaling and absorption corrections were performed using *CrysAlis^{Pro}* 1.171.44.92a[3]. The final completeness is 99.90 % out to 66.595° in Θ. An analytical absorption correction was performed using *CrysAlis^{Pro}* 1.171.44.92a[3]. The analytical numeric absorption correction was done using a multifaceted crystal model based on expressions derived by R.C. Clark & J.S. Reid.[8] The empirical absorption correction was done using spherical harmonics, implemented in SCALE3 ABSPACK scaling algorithm. The absorption coefficient μ of this crystal is 7.177 mm^{-1} at this wavelength

(λ = 1.54184 Å) and the minimum and maximum transmissions are 0.607 and 0.816. The structure was solved and the space group $P2_1/c$ (# 14) determined by the *ShelXT*[4] structure solution program using dual methods and refined by full matrix least squares minimisation on $\boldsymbol{F^2}$ using *ShelXL-2019/3*[7], using *Olex2* 1.5[5] as the graphical interface. All non-hydrogen atoms were refined anisotropically. Hydrogen atom positions were calculated geometrically and refined using the riding model. There is a single formula unit in the asymmetric unit, which is represented by the reported sum formula. In other words: Z is 4 and Z' is 1. The moiety formula is $C_{40}H_{51}IrN_2O_2$.

Table S10. Crystal data and structure refinement for **11**.

Compound	DW555
Formula	$C_{40}H_{51}IrN_2O_2$
$D_{calc.}$/ g cm^{-3}	1.395
μ/mm^{-1}	7.177
Formula Weight	784.03
Color	clear intense yellow
Shape	plate
Size/mm^3	0.13×0.04×0.04
T/K	139.99(10)
Crystal System	monoclinic
Space Group	$P2_1/c$
a/Å	14.9822(5)
b/Å	14.0417(3)
c/Å	18.0456(6)
α/°	90
β/°	100.458(3)
γ/°	90
V/Å^3	3733.3(2)
Z	4
Z'	1
Wavelength/Å	1.54184
Radiation type	CuK_α
Θ_{min}/°	4.015
Θ_{max}/°	66.595
Measured Refl's.	18428
Indep't Refl's	6599
Refl's I≥2 σ(I)	4569
R_{int}	0.0946
Parameters	419
Restraints	410
Largest Peak	1.835
Deepest Hole	–1.210
GooF	1.023
wR_2 (all data)	0.1622
wR_2	0.1414
R_1 (all data)	0.0928
R_1	0.0607
CCDC number	2425612

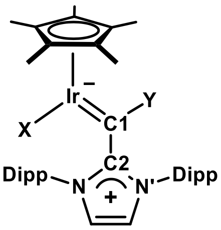

Table S11 Comparison of the selected bond length, bond angle and torsion angle.

	**2**	**6**	**8**
Ir–C1 (Å)	1.890(3), 1.893(4)	1.811(6)	1.921(8)
IrC1–C2 (Å)	1.448(5), 1.452(5)	1.496(6)	1.447(10)
Ir–X (Å)	2.3818(12), 2.3769(11)	2.3707(15)	2.377(2)
IrC1–Y (Å)	1.815(3), 1.825(4)	1.423(6)	1.426(9)
Ir-C1-C2 (°)	129.2(2), 126.9(3)	133.2(4)	125.6(5)
X-Ir-C1 (°)	90.21(11), 91.80(11)	88.80(16)	90.8(2)
Ir-C1-C2-N (°)	–52.9(4), –56.8(4)	–67.2(6)	–60.6(10)
Ir-C1-C2-N' (°)	126.0(3), 121.0(3)	113.2(5)	121.1(7)

Anharmonic Refinement Justification:

In the structures **2429378** and **2429379**, residual electron density peaks were consistently observed in the vicinity of the Iridium metal centers, despite careful refinement of disorder and solvent molecules. The application of anharmonic displacement parameters using *olex2.refine 1.*5,[6] significantly improved the residual maps, reducing the largest residual peaks and resulting in chemically more meaningful models. While this feature of *Olex2.refine* is labelled as "experimental," it is well-documented and has shown practical utility, particularly for heavy atoms like Iridium where harmonic models sometimes fail to capture the full electron density behaviour.

Regarding Kuhs' rule,[9] we acknowledge that in these particular cases, the calculated required sin(θ)/λ values exceed the maximum resolution limits of our datasets. Therefore, strictly speaking, these refinements do not fulfil Kuhs' criterion for statistically significant anharmonic modelling. However, we have included the rule-based calculations, and we clearly state that the refinement was applied primarily for empirical improvement of the model, not as a definitive confirmation of physical anharmonic motion.

Below, we compare the required sin(θ)/λ values against the experimental limits for the relevant structures.

Table S12. Summary of Kuhs' rule.

Structure	Atom	U_eq (Å^2)	Radiation	Resolution (Å)	Required sin(θ)/λ	Experimental sin(θ)/λ	Meets Req?
DW438	Ir1	0.0265	Mo Kα	0.7000	2.8211	0.7143	No
DW450	Ir1	0.0225	Mo Kα	0.7000	3.3211	0.7143	No
DW450	Ir2	0.0237	Mo Kα	0.7000	3.1517	0.7143	No

7. Computational details

General:

All calculations were performed using the Gaussian 16 package (revision C.01).[10] The geometry optimizations and vibration frequencies were calculated by using M062X[11] level of theory. The LanL2DZ basis set and pseudopotential were employed for the Ir atom,[12,13] and the 6-31G(d,p) basis set for C, H, N, O and Cl atoms.[14-18] All local minima were confirmed with no imaginary frequency (Number of imaginary frequencies (NIMAG): 0). The NBO analyses[19] were performed with the Version 3.1 of the NBO program, which was implemented in the G16 C.01 version of the Gaussian program.[20] All calculations were performed in gas phase.

Complex **3⁺** and **9⁺** means complex **3** and **9** without anion.

Table S13. Calculated absolute energies, E(SCF), and free energies at 298 K, G^{298}, for complexes of interest (calculated at the M062X/LanL2DZ,6-31G(d,p) level).

Complex	E(SCF) (a.u.)	NIMAG, ZPVE (kJ mol^{-1})	G^{298} (a.u.)
**2**	–2612.55013305	0, 2129	–2611.819016
**3^{+}**	–2152.15627828	0, 2129	–2151.424831
**5**	–1692.13719337	0, 2120	–1691.409055
**9^{+}**	–1731.89689015	0, 2232	–1731.122234
**10**	–1880.68071202	0, 2166	–1879.934360
**11**	–1918.78825612	0, 2169	–1918.043334

Complex **2**:

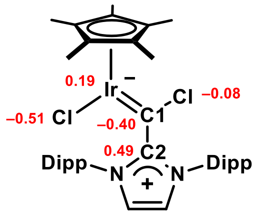

2

Figure S64 Calculated NPA charges of complex **2** on selected atoms (calculated at the M062X/LanL2DZ,6-31G(d,p) level).

Table S14. Comparison of the structural parameters of complex **2**; experimental data from XRD and computed data (calculated at the M062X/LanL2DZ,6-31G(d,p) level).

	**2** (exp)	**2** (calc)
Ir–C1 (Å)	1.890, 1.893	1.870
WBI	--	1.24
C1–C2 (Å)	1.449, 1.453	1.446
WBI	--	1.12
Ir-C1-C2(°)	126.0, 121.3	125.0

Table S15. NBO analysis of the Ir-C1-C2 bond of complex **2** (calculated at the M062X/LanL2DZ,6-31G(d,p) level).

	Occupation Number		%	%s	%p	%d
Ir-C1(σ)	1.87	Ir	30.88	46.99	12.90	40.12
		C1	69.12	43.01	56.98	0.02
Ir-C1(π)	1.85	Ir	44.94	0.77	5.60	93.63
		C1	55.06	1.92	98.08	0.01
C1-C2	1.97	C1	46.02	34.44	65.49	0.07
		C2	53.98	42.61	57.37	0.02

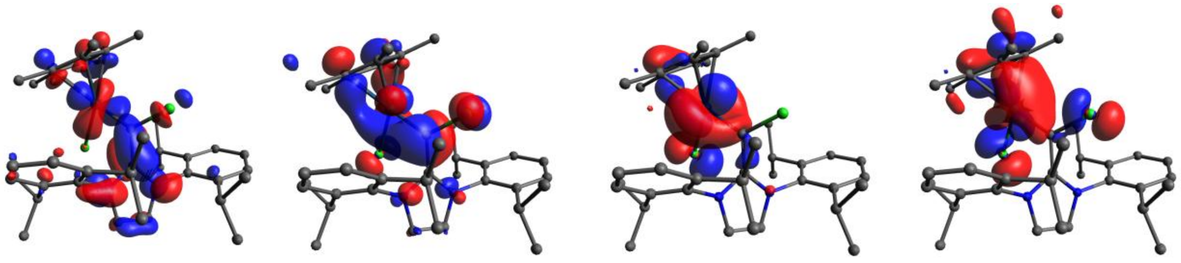

LUMO (-0.340 eV) HOMO (–5.278 eV) HOMO-1 (–6.835 eV) HOMO-2 (–7.175 eV)

Figure S65 Selected surface diagrams of the frontier Kohn-Sham orbitals of the cationic part of complex **2** (M062X/LanL2DZ,6-31G(d,p)), at an isodensity value of 0.04. Color code: deep-blue, nitrogen; gray, carbon; green, chlorine; pale-blue, iridium. Hydrogen atoms are omitted.

Complex $\mathbf{3^+}$:

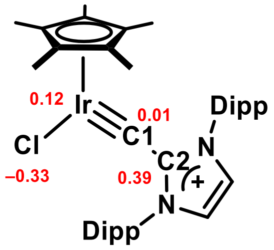

$\mathbf{3^+}$

Figure S66 Calculated NPA charges of complex $\mathbf{3^+}$ on selected atoms (calculated at the M062X/LanL2DZ,6-31G(d,p) level).

Table S16. Comparison of the structural parameters of complex $\mathbf{3^+}$; experimental data from XRD and computed data (calculated at the M062X/LanL2DZ,6-31G(d,p) level).

	$\mathbf{3^+}$ (exp)	$\mathbf{3^+}$ (calc)
Ir–C1 (Å)	1.743	1.725
WBI	--	1.96
C1–C2 (Å)	1.418	1.418
WBI	--	1.16
Ir-C1-C2(°)	172.4	173.6

Table S17. NBO analysis of the Ir-C1-C2 bond of complex **3⁺** (calculated at the M062X/LanL2DZ,6-31G(d,p) level).

	Occupation Number		%	%s	%p	%d
Ir-C1(σ)	1.95	Ir	37.35	35.80	9.70	54.50
		C1	62.56	57.63	42.34	0.03
Ir-C1(1π)	1.90	Ir	70.22	3.37	3.55	93.08
		C1	29.78	0.08	99.82	0.09
Ir-C2(2π)	1.88	Ir	58.53	0.17	2.31	97.52
		C1	41.47	0.03	99.93	0.04
C1-C2	1.97	C1	53.42	39.63	60.33	0.03
		C2	46.58	41.92	58.02	0.06

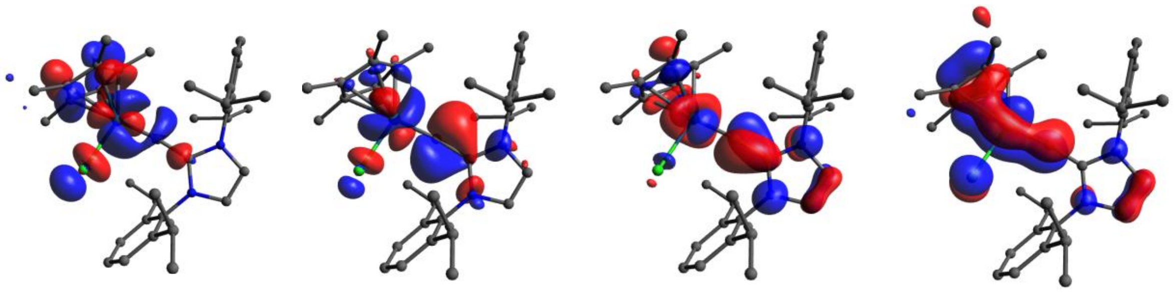

LUMO+2 (–2.898 eV) LUMO+1 (–3.877 eV) LUMO (–4.430 eV) HOMO (–9.283 eV)

Figure S67 Selected surface diagrams of the frontier Kohn-Sham orbitals of the cationic part of complex **3⁺** (M062X/LanL2DZ,6-31G(d,p)), at an isodensity value of 0.04. Color code: deep-blue, nitrogen; gray, carbon; green, chlorine; pale-blue, iridium. Hydrogen atoms are omitted.

<u>Complex **5**:</u>

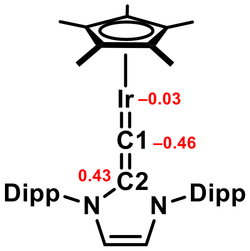

5

Figure S68 Calculated NPA charges of complex **5** on selected atoms (calculated at the M062X/LanL2DZ,6-31G(d,p) level).

Table S18. Comparison of the structural parameters of complex **5**; experimental data from XRD and computed data (calculated at the M062X/LanL2DZ,6-31G(d,p) level).

	**5** (exp)	**5** (calc)
Ir–C1 (Å)	1.744	1.756
WBI	--	1.97
C1–C2 (Å)	1.402	1.395
WBI	--	1.28
Ir-C1-C2(°)	167.0	164.3

Table S19. NBO analysis of the Ir-C bond of complex **5** (calculated at the M062X/LanL2DZ,6-31G(d,p) level).

	Occupation Number		%	%s	%p	%d
Ir-C1(σ)	1.97	Ir	29.23	76.08	9.86	14.06
		C1	70.77	61.83	38.15	0.02
Ir-C1(1π)	1.93	Ir	54.27	0.65	3.76	95.59
		C1	45.73	0.46	99.51	0.03
Ir-C1(2π)	1.76	Ir	51.7	0.81	2.82	96.37
		C1	48.3	0.30	99.68	0.01
C1-C2	1.97	C1	45.51	37.35	62.59	0.06
		C2	54.49	44.06	55.92	0.02

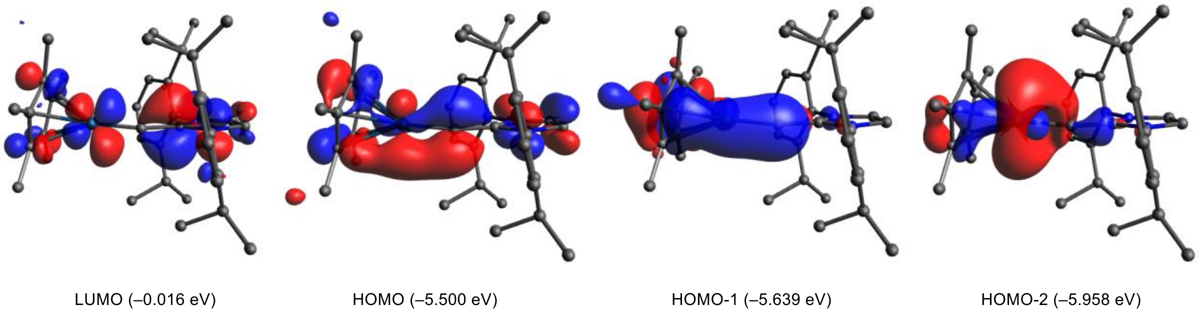

Figure S69 Selected surface diagrams of the frontier Kohn-Sham orbitals of complex **5** (M062X/LanL2DZ,6-31G(d,p)), at an isodensity value of 0.04. Color code: deep-blue, nitrogen; gray, carbon; pale-blue, iridium. Hydrogen atoms are omitted.

Complex **9⁺**:

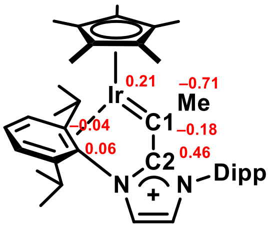

9⁺

Figure S70 Calculated NPA charges of complex **9⁺** on selected atoms (calculated at the M062X/LanL2DZ,6-31G(d,p) level).

Table S20. Comparison of the structural parameters of complex **9⁺**; experimental data from XRD and computed data (calculated at the M062X/LanL2DZ,6-31G(d,p) level).

	**9⁺** (exp)	**9⁺** (calc)
Ir–C1 (Å)	1.942	1.891
WBI	--	1.21
C1–C2 (Å)	1.435	1.450
WBI	--	1.14
Ir-C1-C2(°)	115.0	115.1

Table S21. NBO analysis of the Ir-C bond of complex **9⁺** (calculated at the M062X/LanL2DZ,6-31G(d,p) level).

	Occupation Number		%	%s	%p	%d
Ir-C1 (σ)	1.83	Ir	32.91	43.80	14.77	41.43
		C1	67.09	35.68	64.30	0.03
Ir-C1(π)	1.79	Ir	62.19	0.64	3.14	96.22
		C1	37.81	0.03	99.93	0.04
C1-C2	1.97	C1	53.66	42.54	57.44	0.02
		C2	46.34	29.31	70.63	0.07

Complex **10**:

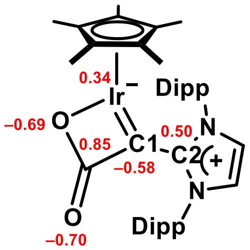

10

Figure S71 Calculated NPA charges of complex **10** on selected atoms (calculated at the M062X/LanL2DZ,6-31G(d,p) level).

Table S22. Comparison of the structural parameters of complex **10**; experimental data from XRD and computed data (calculated at the M062X/LanL2DZ,6-31G(d,p) level).

	**10** (exp)	**10** (calc)
Ir–C1 (Å)	1.946	1.941
WBI	--	1.06
C1–C2 (Å)	1.436	1.413
WBI	--	1.21
Ir-C1-C2(°)	149.2	149.6

Table S23. NBO analysis of the Ir-C1-C2 bond of complex **10** (calculated at the M062X/LanL2DZ,6-31G(d,p) level).

	Occupation Number		%	%s	%p	%d
Ir–C1	1.83	Ir	31.55	33.07	27.84	39.09
		C1	68.45	32.31	67.68	0.01
C1–C2 (σ)	1.97	C1	46.68	35.33	64.62	0.05
		C2	53.32	44.16	55.82	0.02
C1–C2 (π)	1.56	C1	67.23	1.82	98.16	0.02
		C2	32.77	0.04	99.92	0.04

Complex **11**:

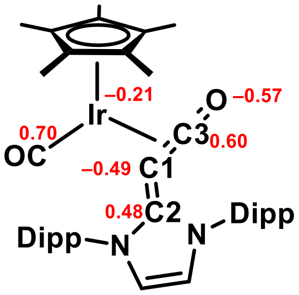

11

Figure S72 Calculated NPA charges of complex **11** on selected atoms (calculated at the M062X/LanL2DZ,6-31G(d,p) level).

Table S24. Comparison of the structural parameters of complex **11**; experimental data from XRD and computed data (calculated at the M062X/LanL2DZ,6-31G(d,p) level).

	**11** (exp)	**11** (calc)
Ir–C1/C3 (Å)	2.089/2.153	2.089/2.149
WBI	--	0.60/0.51
C1–C2 (Å)	1.404	1.381
WBI	--	1.31
C1–C3 (Å)	1.307	1.346
WBI	--	1.46
C3–O	1.216	1.201
WBI	--	1.72
C2-C1-C3(°)	132.6	133.7
C1-C3-O(°)	159.3	158.7

Table S25. NBO analysis of the selected bonds and lone pair of complex **11** (calculated at the M062X/LanL2DZ,6-31G(d,p) level).

	Occupation Number		%	%s	%p	%d
Ir–C1	1.79	Ir	43.94	23.44	11.06	65.49
		C1	56.06	27.93	72.04	0.02
Ir–C3	1.71	Ir	64.94	20.69	6.98	72.33
		C3	35.06	14.00	85.91	0.09
C1–C3	1.95	C1	52.06	31.12	68.83	0.05
		C3	47.94	50.27	49.69	0.04
C1-C2	1.96	C1	48.35	39.54	60.42	0.04
		C2	51.65	43.48	56.5	0.02
C3–O (σ)	1.99	C3	34.53	35.44	64.46	0.10
		O	65.47	43.86	55.80	0.34
C3–O(π)	1.99	C3	27.40	0.33	99.46	0.21
		O	72.60	0.33	99.32	0.35
C1	1.29		--	1.29	98.66	0.05

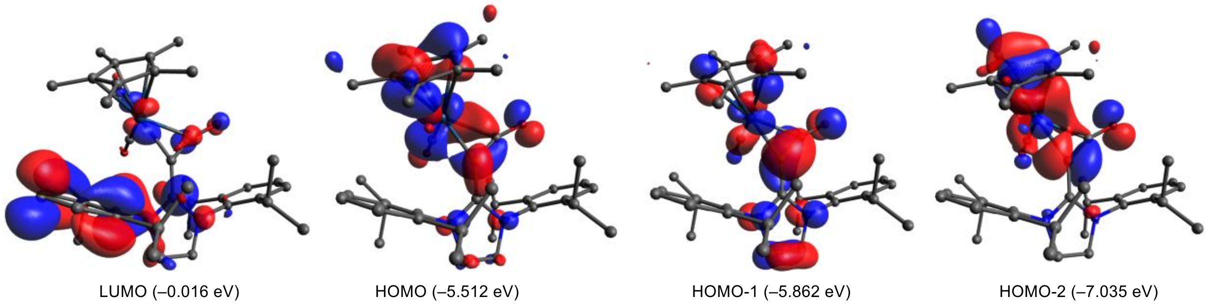

Figure S73 Selected surface diagrams of the frontier Kohn-Sham orbitals of complex **11** (M062X/LanL2DZ,6-31G(d,p)), at an isodensity value of 0.04. Color code: deep-blue, nitrogen; red, oxygen; gray, carbon; pale-blue, iridium. Hydrogen atoms are omitted.

Bonding situation in complex **11**:

The bonding situation in complex **11** can be described by the valence formulas depicted in **Figure S74**. The structures **A** and **D** reflect the short bond between C1 and C3 (1.302 Å; even shorter than a typical C=C double bond; WBI = 1.46), and the π-character of the NBO associated with the Ir-C3 bond. The structures **B**, **D**, and **E** reflect the partial single bond character of the C1–C2 bond (1.402 Å; WBI = 1.31). The structures **B**, **C**, and **E** reflect the σ-character of the NBO associated with the Ir-C1 bond. Structure **E** reflects the pyramidalization at C1 (the sum of the angles is 348.5°), and the lone pair character of the NBO associated with C1.

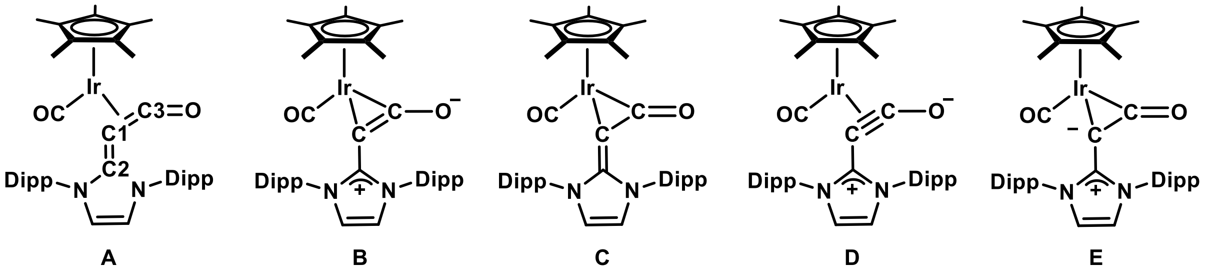

Figure S74 Resonance structures for complex **11**.

Complex **2** (0 1, M062X/LanL2DZ,6-31G(d,p))
Ir -1.3946984289 -0.8879643510 -0.2906957279
Cl -1.2003705824 -0.8652264371 2.1822146532
Cl 1.3500753385 -0.4968995455 -1.9856402696
N 0.6485444414 1.6744780067 1.0418641594
N 2.2798417414 0.2415093133 1.0763086955
C 5.6695315304 1.7636918282 0.1683365739
H 6.6092449460 1.2531033347 -0.0660866010
H 5.5375465853 1.7350212823 1.2537922969
H 5.7659878352 2.8097648938 -0.1380197571
C 1.0819647902 0.5033980755 0.4955297814
C -2.0106294461 0.0259493203 -3.3088934252
H -1.9926505182 1.0802236596 -3.0171761229
H -2.7087802609 -0.0900814880 -4.1449218167
H -1.0076496037 -0.2396140133 -3.6510694201
C 4.4955927991 1.0984028705 -0.5601936974
H 3.5893314159 1.6585873073 -0.3082233693
C -3.5999613107 -1.5963173583 -0.2904312705
C -1.4984232843 3.5514270875 3.6542703654
H -2.2358686606 4.3417781724 3.4775458740
H -0.5066263131 4.0007631711 3.5395291730
H -1.6035246619 3.2164177607 4.6902168578
C -0.3869662624 2.5240923086 0.4933961891
C 4.3185067315 -0.3423100450 -0.1048483563
C -2.4174626990 -0.8265744713 -2.1484860992
C -2.7399901219 -2.6472436509 -0.7174637296
C 3.0280445337 -2.1002178404 1.0471817553
C 1.5436402173 2.0960930629 2.0068418920
H 1.3727853647 2.9928798245 2.5797927237
C -2.6314780649 -3.9925433398 -0.0730925024
H -1.6608968483 -4.4492872004 -0.2800713699
H -3.4118432001 -4.6707292668 -0.4375948076
H -2.7330762841 -3.9030230718 1.0117052761
C -1.5169048777 2.8372620687 1.2587815913
C 1.0047937190 -3.5885976800 1.0866201103
H 0.7360708438 -3.2048597737 0.0976113770
H 0.0776302848 -3.7864411523 1.6332203829
H 1.5524953629 -4.5312658254 0.9702635091
C 1.8292536193 -2.5497549478 1.8538071402
H 1.1731404780 -1.6944401056 2.0207025142
C 2.5624849141 1.2041964754 2.0257079062
H 3.4576907535 1.1527240064 2.6236729656
C -0.1595154560 3.0761080009 -0.7840545852
C 5.2629430379 -1.3033324490 -0.4619608145
H 6.1261492299 -1.0077078185 -1.0510506069
C 1.0280776538 2.8963798202 -3.0380664409
H 0.7303924866 3.8675550435 -3.4464243909
H 0.3001239052 2.1422730084 -3.3484740918
H 1.9933111271 2.6358132168 -3.4822935302
C 1.1657458192 2.9296262356 -1.5159152369

H 1.6271625633 1.9826166886 -1.2373158089
C 3.2238661680 -0.7692360199 0.6656224346
C 3.9978318045 -3.0249431637 0.6497442714
H 3.8743577276 -4.0669558948 0.9301551524
C -3.1036270096 1.7624998142 2.9032834608
H -3.1626665223 1.3222584189 3.9025747211
H -3.2813529478 0.9639058895 2.1796368776
H -3.8950032747 2.5158672438 2.8206504659
C -1.7161195698 2.3761381765 2.6905090472
H -0.9870420727 1.5928354305 2.9057740333
C -4.4607460037 -1.6508509217 0.9353470687
H -3.8430026656 -1.7513994650 1.8342887775
H -5.1456038923 -2.5029741903 0.8845950109
H -5.0606597423 -0.7439274373 1.0353766056
C 5.1062856447 -2.6336754626 -0.0878573768
H 5.8497710867 -3.3682514179 -0.3807232567
C -2.3093527108 4.1863207308 -0.5998277368
H -3.0702220845 4.8278445624 -1.0333776281
C -1.1559570251 3.8906156019 -1.3200047431
H -1.0157715266 4.3216137055 -2.3055656276
C -2.4721893697 3.6808858888 0.6797404698
H -3.3586007496 3.9386545947 1.2511661280
C 4.6743661536 1.1950105835 -2.0792001230
H 5.5992969616 0.7092139363 -2.4052252326
H 4.7310265279 2.2455556728 -2.3828821379
H 3.8360013866 0.7254574170 -2.5991248424
C 0.3583625123 -0.2687308155 -0.4901749658
C -2.0087068025 -2.1856071818 -1.8852765520
C 2.1142371629 4.0551683984 -1.0760806783
H 3.0854725007 3.9503484955 -1.5716621772
H 2.2803731253 4.0367177408 0.0060143456
H 1.6985405093 5.0335374736 -1.3388539718
C -3.3960099005 -0.4619628682 -1.1344414577
C -1.0952520383 -3.0185866712 -2.7263208502
H -0.3845173880 -2.3922607138 -3.2679049303
H -1.6710864941 -3.6038793970 -3.4514491666
H -0.5197973412 -3.7111958204 -2.1069340676
C -4.0875575097 0.8621306605 -1.0446449115
H -4.3496461220 1.1019829819 -0.0113742077
H -5.0056582540 0.8678712542 -1.6440375218
H -3.4351703263 1.6617149034 -1.4038516970
C 2.2548227246 -3.0853371689 3.2249787314
H 2.8761399562 -3.9827145822 3.1294264729
H 1.3681219112 -3.3458574322 3.8096355714
H 2.8256353945 -2.3379994120 3.7841582342

Complex **3**$^{+}$ (1 1, M062X/LanL2DZ,6-31G(d,p))

```
Ir 0.1074179323 1.5051858674 -0.1425504009
Cl 2.4314175412 1.5359135263 -0.6961897369
N -1.0583922686 -2.4335305087 0.0960608211
N 1.1166637547 -2.3913210274 0.1992551869
C -2.4169912444 -1.9734951235 -0.0612628930
C 1.8683958044 0.1845227050 3.2302956394
H 1.1405809505 0.2387917476 4.0465167003
H 1.5475930493 0.8483298618 2.4210364761
H 2.8256286052 0.5506781969 3.6135733308
C 0.0184647371 -1.6234262494 0.0805371992
C -3.2027483620 -1.8291756556 1.0929429894
C -0.9687359597 2.8497466714 2.6970997271
H -1.5579825813 1.9592556898 2.9213475016
H -1.4852480895 3.7160546779 3.1234753830
H -0.0016348172 2.7527988562 3.1934697106
C 2.8858604917 -1.3442678198 1.5064191784
C -1.7581985719 -0.5719221429 -3.3146170218
H -2.7000306121 -0.1386472920 -3.6682367331
H -1.1151768725 -0.7283629386 -4.1853366200
H -1.2670138106 0.1459580386 -2.6496557465
C -0.6316406957 -3.7398034261 0.2236979081
H -1.3339981412 -4.5575683192 0.2551001763
C 4.5796772478 -1.4957120237 -0.7377110445
H 5.2465894248 -1.5389207239 -1.5910722755
C -2.8679566857 -1.7033194769 -1.3593718761
C 3.8137968834 -3.1062261799 -3.0738670783
H 4.4868630209 -2.3375797957 -3.4642270763
H 3.3478038359 -3.5931752057 -3.9339164107
H 4.4123826359 -3.8492413951 -2.5405445127
C 2.4702853584 -1.8884787356 0.2867919903
C 0.8724609934 4.3929311719 -1.8602584718
H 1.8972244209 4.0271274093 -1.7523603503
H 0.8771844926 5.4793881580 -1.7278630069
H 0.5350033964 4.1732191763 -2.8739819080
C 4.2005038230 -0.8776125441 1.5660409923
H 4.5713553699 -0.4495013382 2.4921502992
C 3.2770980614 -1.9751330486 -0.8572138263
C -3.0473950572 1.9166082243 0.4197178479
H -3.2853562474 1.2372561653 -0.4025102031
H -3.8471023655 2.6598010261 0.5006030456
H -3.0318733425 1.3254755464 1.3374241350
C -0.0280349107 -0.2105399406 -0.0321853764
C -4.9955561182 -1.0945173234 -0.3648491099
H -6.0181596666 -0.7520396392 -0.4850429909
C 2.7333789205 -2.4976233515 -2.1777268601
H 2.0075336695 -3.2915696875 -1.9609293662
C -2.0054737837 -1.9026914935 -2.5950256982
H -1.0288427805 -2.2935641584 -2.2855439645
C -4.1853070760 -1.2509990509 -1.4830386159
```

H -4.5818332215 -1.0345127502 -2.4705818776
C -1.2476327536 3.0956489700 -1.0802516848
C 0.7265752086 -3.7110838042 0.2900967406
H 1.4549092382 -4.5000326065 0.3922516097
C 2.5032854577 -2.1881865821 3.8413209140
H 3.5040355590 -1.8890617301 4.1666313698
H 2.5582963866 -3.2254178638 3.4992255763
H 1.8405370109 -2.1455600244 4.7102890732
C 1.9915587304 -1.2608132455 2.7321542669
H 0.9839210543 -1.5996650974 2.4617151695
C -2.6353852818 -2.9355009440 -3.5377443747
H -2.8070687730 -3.8879770344 -3.0294252629
H -1.9772474714 -3.1140005180 -4.3919607773
H -3.5955702988 -2.5824386891 -3.9252340997
C 0.2633657190 3.7166506638 0.5708763035
C 1.4823312488 4.2975713064 1.2091048904
H 1.6414334672 3.8889808889 2.2085092729
H 1.3822601027 5.3844030716 1.2943260540
H 2.3673888029 4.0756594311 0.6079604655
C -2.6380567007 -2.1004438414 2.4788856787
H -1.8364343635 -2.8413587311 2.3814588417
C -2.0169457688 -0.8196630306 3.0573499177
H -1.2768157654 -0.3765307979 2.3791376641
H -1.5259211259 -1.0287450785 4.0127776051
H -2.8030236789 -0.0774982297 3.2396250388
C -4.5109371601 -1.3812199679 0.9089333630
H -5.1619271306 -1.2605808891 1.7680125496
C -3.6756153261 -2.6758956863 3.4456650957
H -4.4357096828 -1.9343809160 3.7079680617
H -3.1865894332 -2.9782180703 4.3748084771
H -4.1798999274 -3.5483105777 3.0229729575
C -0.0174750265 3.7604785807 -0.8414664810
C 1.9948172819 -1.3741035399 -2.9227031001
H 1.1832529698 -0.9427075850 -2.3270855713
H 1.5760791612 -1.7545637066 -3.8600356735
H 2.6858611779 -0.5580265103 -3.1530765771
C -1.7298203540 2.5932723436 0.1951069911
C -1.9485011579 2.9592440564 -2.3924723370
H -1.2423345573 2.7705560854 -3.2036782904
H -2.4912833958 3.8827513460 -2.6196040374
H -2.6695755006 2.1402759148 -2.3701519546
C -0.8051339994 3.0363929286 1.2242293200
C 5.0355896546 -0.9542128500 0.4604191297
H 6.0533346577 -0.5848876777 0.5285615942

Complex **5** (0 1, M062X/LanL2DZ,6-31G(d,p))
Ir -0.9814792454 -1.2121527818 -0.2279160463
N 0.6617135084 2.3682441067 0.1581666812
N 2.4744025952 1.1695198793 0.1487290661
C 1.1163915988 1.0840935748 0.0371773715
C 3.4788762043 -0.0404850153 3.7624387589
H 4.1258999863 -0.8762211044 4.0480834776
H 2.8690620893 0.2226263311 4.6320635688
H 4.1191670794 0.8126678477 3.5192351424
C -2.1221262002 -2.6276897276 2.5032984013
H -1.1944244033 -3.1618260248 2.7221945327
H -2.9520219781 -3.1919873159 2.9446987477
H -2.0677673711 -1.6553072976 3.0004379726
C -3.0291687283 -1.6404202039 -1.0159788318
C -3.4072309391 3.3415307894 0.0579837639
H -4.4642475746 3.5881515307 0.0332023575
C -0.7276426173 2.7181048025 0.1210670202
C -3.7284521252 -0.8121925249 -2.0474599364
H -3.2438403163 -0.9037291433 -3.0225168967
H -3.7123532051 0.2459283431 -1.7689455578
H -4.7738653385 -1.1237583435 -2.1590078143
C 3.3485511823 0.0329897648 0.1755788895
C 5.1333058192 0.5654764047 -3.1516884024
H 5.3360333114 -0.4179962111 -3.5868477439
H 6.0235349957 0.8870660144 -2.6035107689
H 4.9717133511 1.2573126430 -3.9826968760
C 4.0963606372 -0.2584726640 -0.9757455151
C -2.7154533813 3.1427278977 -1.1306063676
H -3.2350841110 3.2375943689 -2.0800998671
C -1.8123178690 -3.3345405595 0.0046384701
C 4.9619204877 -1.3496426885 -0.9166606255
H 5.5614470326 -1.6072307968 -1.7830637623
C -2.3089099059 -2.4594828301 1.0283901541
C 4.2899218963 -1.8175606128 1.3527411851
H 4.3669227153 -2.4324612479 2.2448110093
C -2.2388853623 -2.8199955562 -1.2534484958
C 1.6564952581 -1.5920852318 2.9283368583
H 1.0082624333 -1.8249937005 2.0777826424
H 1.0272438980 -1.3308969405 3.7858736626
H 2.2359483283 -2.4823444444 3.1967477359
C -1.3988023886 2.8990080264 1.3395816963
C 2.8547728759 2.4885521376 0.3432856573
H 3.8907188444 2.7614390659 0.4632593921
C -1.3551649067 2.8242707524 -1.1248903330
C -1.9287166138 -3.3980436079 -2.5987960963
H -1.8291682366 -2.6083718649 -3.3474197962
H -2.7197204334 -4.0815702771 -2.9286253392
H -0.9876983688 -3.9517185459 -2.5793352923
C 2.5800799293 -0.4191026889 2.5795086984
H 1.9396345662 0.4417838591 2.3599999598

C -2.7550782182 3.2198285936 1.2813345630
H -3.3118682161 3.3650618128 2.2015558209
C 1.7263008282 3.2360500182 0.3378177565
H 1.5738651684 4.2976490795 0.4473004119
C 5.0597929607 -2.1201798494 0.2383178805
H 5.7377588241 -2.9674621308 0.2640679118
C -0.6947018335 2.6844185033 2.6695733680
H 0.3802658603 2.8238149985 2.5106759148
C -3.8566705086 -0.3429280176 1.0843767534
H -3.4044241515 -0.0948523555 2.0472647388
H -4.8934513811 -0.6527348688 1.2637146077
H -3.8649351876 0.5703654147 0.4842804497
C 0.3380771470 -0.0552209895 -0.1676621947
C -0.9713357915 -4.5525559157 0.2298534958
H -0.3340707379 -4.7534401355 -0.6339109559
H -1.5926367138 -5.4381488489 0.4074238368
H -0.3159760223 -4.4195040488 1.0939546334
C 3.8937433514 0.5345676031 -2.2561967120
H 3.6610286093 1.5697643255 -1.9808002508
C 2.6862011161 -0.0281346567 -3.0237348457
H 2.4825946288 0.5813529168 -3.9105609048
H 1.7852316161 -0.0685481302 -2.4027741790
H 2.9019175062 -1.0501987586 -3.3531402177
C 3.4120011912 -0.7304417848 1.3469043976
C -1.1298122155 3.6854948159 3.7426679681
H -2.1689529749 3.5239276160 4.0450558379
H -1.0336552979 4.7177911180 3.3946005375
H -0.5116437502 3.5637042528 4.6364808703
C -3.0842622301 -1.4223568176 0.3957400205
C -1.2231624141 1.5021480462 -3.2671315431
H -2.2519419632 1.7415652146 -3.5593241895
H -1.2219760941 0.5698932828 -2.6938577811
H -0.6413630940 1.3563795483 -4.1829937620
C -0.6078116767 2.6292886894 -2.4323489936
H 0.4208927726 2.3345176694 -2.2002841393
C -0.5533124728 3.9449483638 -3.2186401991
H 0.0215301603 3.8141144691 -4.1401509507
H -0.0870191739 4.7414906315 -2.6310518391
H -1.5593914997 4.2768816470 -3.4952479576
C -0.9066771895 1.2404873320 3.1496826673
H -0.3044603108 1.0449631640 4.0441887387
H -0.6439601846 0.5150499364 2.3701039212
H -1.9602480875 1.0891366019 3.4125336493

Complex **9⁺** (1 1, M062X/LanL2DZ,6-31G(d,p))

Ir -1.3249217731 -0.1657779127 -0.5610867580
N 0.5226956077 0.4763721874 1.6181679901
N 2.5555718542 0.3070671864 0.8965518069
C -0.9130803025 0.3102659298 1.5256222519
C -1.3544736108 -1.0756491925 1.4966254682
C 3.7232643682 0.1350176327 0.0666792705
C 1.3355898374 0.5843843561 2.7241494614
H 0.9421222303 0.7333983190 3.7172478244
C 2.6122282649 0.5025294967 2.2678748063
H 3.5594431750 0.5738225611 2.7794631803
C -2.7458223532 0.9942575997 -1.8624666436
C 1.3841392004 -0.1975526270 -1.9677783004
H 0.8054661322 -0.6886136391 -2.7471919115
H 2.2822122595 -0.7974366195 -1.7926195385
H 1.7278051043 0.7703732641 -2.3587058463
C -3.6325742745 0.2443408803 -1.0294175887
C 4.1509122138 1.2216331185 -0.7111436498
C 1.2557500092 0.2709874065 0.5119843032
C 0.5542819730 -0.0276295377 -0.7212940201
C -2.3714055490 -1.2662425666 -2.2653733804
C -3.3904244509 -1.1412764005 -1.2626492258
C -1.9690778696 0.0583498508 -2.6476119583
C -1.7065736416 1.3703974632 2.1440358353
C 4.3598266977 -1.1137028653 0.0824385943
C 5.2800829818 1.0217564891 -1.5050645535
H 5.6477486914 1.8293042616 -2.1279070749
C -4.1409817484 -2.2916155745 -0.6678998810
H -3.4724428700 -3.1201640884 -0.4201533004
H -4.8809141782 -2.6623933911 -1.3847622378
H -4.6713516374 -1.9980621383 0.2388726276
C -0.3847005859 -2.2546734014 1.6414317262
H 0.6012903984 -1.9390376384 1.2906882993
C -2.7754638759 2.4728247740 -2.0844370723
H -3.2796569560 2.9872300164 -1.2645725574
H -3.3271729893 2.6953870238 -3.0052876647
H -1.7697098484 2.8869057596 -2.1865711998
C -2.6853770443 -1.3066360547 2.0229333613
H -3.0440276539 -2.3303750109 2.0689570884
C -4.6943160656 0.8548991470 -0.1701610753
H -5.1370294329 0.1246091525 0.5076517082
H -5.4946414793 1.2562991311 -0.8006395228
H -4.2962843368 1.6750088508 0.4340308409
C 5.4861563345 -1.2570954475 -0.7313288922
H 6.0112577141 -2.2057062636 -0.7558150646
C -1.9083786456 -2.5391267464 -2.9016190027
H -0.8418923311 -2.5031850727 -3.1391414891
H -2.4544164187 -2.7174728379 -3.8344341860
H -2.0810643031 -3.3958585188 -2.2470892442
C 3.8123873987 -2.2922790357 0.8741439991

H 3.2373218956 -1.9045585047 1.7230008632
C -1.1769716998 0.4455421327 -3.8596816751
H -0.4642283547 1.2457364312 -3.6468829511
H -1.8644559742 0.8062207336 -4.6322246133
H -0.6322192875 -0.4017854002 -4.2793809033
C -1.1232691298 2.7705118832 2.2412724542
H -0.1647489599 2.6954241456 2.7718607506
C -0.7933893928 -3.4615389831 0.7983687175
H -1.7753929894 -3.8468940549 1.0929095348
H -0.0750464718 -4.2743101580 0.9361647586
H -0.8226117595 -3.1983395591 -0.2628871761
C -2.9419687172 1.0409932290 2.6122907078
H -3.5686766452 1.7969549709 3.0710268360
C 3.4310763097 2.5609329961 -0.6687237623
H 2.3589155857 2.3653238370 -0.5382227307
C -0.2533947012 -2.6394683549 3.1220352696
H 0.0091029235 -1.7757402355 3.7408122866
H 0.5277490273 -3.3952805355 3.2446795543
H -1.1881572836 -3.0554411596 3.5088396890
C -3.4294456819 -0.3042275739 2.5541768002
H -4.4023784138 -0.5247949966 2.9834089442
C 5.9403327808 -0.2027433532 -1.5137019532
H 6.8165294266 -0.3360890351 -2.1394086727
C 2.8526184632 -3.1138964659 -0.0002718202
H 2.0012349737 -2.5183983301 -0.3486389129
H 2.4611351754 -3.9656072727 0.5649777292
H 3.3769455613 -3.5012617602 -0.8798094656
C -0.8260082570 3.3482797885 0.8502669467
H -0.1955323358 2.6850530502 0.2513332331
H -0.3292390013 4.3178939231 0.9447336511
H -1.7619321923 3.4963896627 0.3038527254
C 3.5887411188 3.3683611004 -1.9586998632
H 4.6106749722 3.7403942313 -2.0768048764
H 2.9301662080 4.2398276428 -1.9297076780
H 3.3376104171 2.7758095715 -2.8433210366
C -2.0034621272 3.7368644447 3.0312776318
H -2.9599042208 3.9019189858 2.5242230789
H -1.5042381057 4.7048509784 3.1145070465
H -2.2067315074 3.3731988438 4.0417547034
C 3.9087883199 3.3854055649 0.5357130231
H 3.7388466155 2.8620417175 1.4805961826
H 3.3795904101 4.3414609200 0.5772433849
H 4.9801152300 3.5906585024 0.4503047578
C 4.9152697384 -3.1888519198 1.4436614232
H 5.4299458614 -3.7446280247 0.6547561511
H 4.4785889649 -3.9249456241 2.1231507614
H 5.6600656614 -2.6102680338 1.9958272378

Complex **10** (0 1, M062X/LanL2DZ,6-31G(d,p))

```
Ir -1.2734420067 -1.2720051529 -0.3339634266
O 0.0560982013 -1.9042616099 -1.8182957034
O 1.8924301388 -0.6401717785 -2.2203187619
N 0.7177007693 2.1604814458 0.3257660525
N 2.4676697413 0.8760954301 0.3269250051
C -0.5787171169 2.7136608781 0.0594781318
C 2.8960935830 2.1496196179 0.6587054694
H 3.9300748223 2.3526373903 0.8826423470
C 1.1159080102 0.8711908040 0.1096005095
C 1.8132361925 2.9531419711 0.6464051209
H 1.7084358427 4.0129571708 0.8114618740
C 3.3046869480 -0.2883007581 0.4472925631
C -3.0443610461 -2.5589554715 -0.8397098879
C -2.6772231485 -1.6779842026 1.2864562575
C -3.1886679407 -0.6250558368 0.4734265939
C 0.3657408810 -0.2319545119 -0.3563566508
C -3.3711801597 -1.1627463687 -0.8635653022
C 3.1228282999 -1.1088449921 1.5702445697
C 0.9214268294 -0.9438752727 -1.5459562672
C -1.2816429939 3.2794387603 1.1406592634
C 4.3064518736 -0.5172363659 -0.5087475353
C -1.0256836561 2.7800984892 -1.2669890028
C 4.9378730084 -2.4910489385 0.7560082249
H 5.5752421533 -3.3621458718 0.8703915083
C -2.5795681991 -2.8787609074 0.4645027384
C -0.7394186358 3.1577976739 2.5602767952
H 0.3249259430 3.4107963870 2.5315842566
C 4.5657407998 0.4262870045 -1.6700365854
H 3.6775303285 1.0509806762 -1.7928027608
C 2.1334895187 -0.7873340815 2.6772909149
H 1.5458136857 0.0862409786 2.3831304119
C 3.9513633833 -2.2254206917 1.6958286190
H 3.8302323972 -2.8826354741 2.5522094252
C 5.1198703645 -1.6358769742 -0.3237693500
H 5.9032319021 -1.8436443977 -1.0455580703
C 5.7827117929 1.3124830871 -1.3664328675
H 5.6625332147 1.8876891979 -0.4433739466
H 5.9559612358 2.0170910279 -2.1854179639
H 6.6827569738 0.6985738549 -1.2533492698
C -2.4544051155 -1.6396887540 2.7639517845
H -1.7062002647 -2.3739694707 3.0672840662
H -3.3877747866 -1.8722027581 3.2890035960
H -2.1126305491 -0.6565741870 3.0900999807
C -2.2506139983 3.4209440373 -1.4910533698
H -2.6274421414 3.4948803594 -2.5073427016
C -0.1941222066 2.3110339544 -2.4508182995
H 0.6869894884 1.7758032340 -2.0905433116
C -2.4750276170 3.9412312220 0.8547909091
H -3.0388058973 4.4071831471 1.6550517897
```

C 4.7780845689 -0.3189341710 -2.9913683727
H 5.7129326870 -0.8897001739 -2.9853526703
H 4.8470287284 0.4040011551 -3.8100287712
H 3.9393291315 -0.9872496160 -3.1847979143
C -0.8427112487 1.7126452039 3.0791148175
H -1.8574611247 1.5240090446 3.4454343744
H -0.1573011923 1.5552458937 3.9185318852
H -0.6205103369 0.9720074073 2.3015293073
C -3.6104886457 0.7406526136 0.8967409878
H -3.1231212153 1.0373602369 1.8254703251
H -4.6952647533 0.7751904700 1.0522350484
H -3.3499349648 1.4768604614 0.1345570178
C -2.9594553674 4.0048837047 -0.4496056247
H -3.8945275233 4.5175399846 -0.6522310233
C -2.0790660506 -4.2057402485 0.9413859332
H -1.6681992186 -4.7894626938 0.1158308234
H -2.8837432219 -4.7847164135 1.4079217894
H -1.2822151298 -4.0768310824 1.6780189854
C -0.9531659629 1.3346765093 -3.3528792645
H -1.8376436393 1.8016663414 -3.8018929733
H -1.2632078094 0.4507731219 -2.7890618463
H -0.2976034902 1.0015620444 -4.1618096447
C 2.8835887551 -0.4203182814 3.9643666802
H 3.4746258212 -1.2665095630 4.3290778612
H 2.1766288544 -0.1395385925 4.7516404937
H 3.5659772170 0.4183617524 3.7975673306
C -3.0360362314 -3.4458505776 -2.0468693059
H -2.4757366774 -2.9779620914 -2.8609966508
H -4.0564719524 -3.6432103819 -2.3901237533
H -2.5583189031 -4.4021359395 -1.8297976399
C 1.1479229826 -1.9324004492 2.9188207175
H 0.5929686212 -2.1516059005 2.0010591917
H 0.4368305201 -1.6474979385 3.7016201472
H 1.6632999214 -2.8400385557 3.2501961897
C -3.9121149977 -0.4036895565 -2.0320828723
H -3.6128414734 0.6471362797 -1.9822421158
H -5.0071680442 -0.4490425998 -2.0459036527
H -3.5401495169 -0.8164914494 -2.9730295566
C -1.3964624252 4.1171190261 3.5518219596
H -1.3260144394 5.1569083980 3.2205494599
H -0.9045840714 4.0340571066 4.5246516289
H -2.4536806613 3.8739089536 3.6990847110
C 0.2954915863 3.5305978894 -3.2444234590
H 0.9412899669 3.2077796942 -4.0656274166
H 0.8641446296 4.2157412849 -2.6082564778
H -0.5452762448 4.0871377240 -3.6720667431

Complex **11** (0 1, M062X/LanL2DZ,6-31G(d,p))

```
Ir -1.3481260561 -1.2166339814 -0.1288283586
N 2.5585169741 0.7462692846 0.7100517773
N 0.8200616016 2.0340646779 0.9696698728
O 1.3435801169 -2.2433293819 -1.2097447926
C 1.9193746252 2.6909627373 1.5203995692
H 1.8139323887 3.6646291469 1.9709938192
O -0.8454698071 -2.0858363332 2.6679818464
C 2.9957728839 1.8976625690 1.3476844842
H 4.0312709833 2.0275681387 1.6175166894
C 3.3889758812 -0.3490373394 0.3046664576
C 1.2112376280 0.8187140042 0.4646121029
C -0.4576043023 2.6620819809 0.7868845455
C -2.7170167295 3.0058479364 1.4901715383
H -3.5505003659 2.7767955080 2.1460330506
C 0.4528168318 -0.1576945244 -0.1517019736
C 0.7316374131 -1.3780717127 -0.6455186644
C 3.4912609883 -1.4614250290 1.1522865038
C 4.0209498528 -0.2777624596 -0.9414534085
C 5.0173734171 -2.4302268501 -0.4607347622
H 5.6639677083 -3.2494885527 -0.7586514915
C -1.9566984515 -1.0672101654 -2.3524695247
C 4.3321676157 -2.4967375353 0.7479755847
H 4.4386901993 -3.3753629351 1.3749749730
C 2.9561863858 0.3132681802 -3.1136061117
H 3.5407790525 -0.4318450464 -3.6630453089
H 2.7012686486 1.1229492316 -3.8049238516
H 2.0347277529 -0.1690710777 -2.7751015747
C -2.8030196783 -0.0989146997 -1.6983037872
C -1.0600904724 -1.7507537207 1.5762117783
C -3.6135808663 -0.7784932132 -0.7667053199
C -1.5045546066 2.3329707839 1.6592111384
C -2.7902696244 1.3701334937 -1.9761353156
H -3.4138864485 1.9129756980 -1.2634768431
H -3.1576670359 1.5831521014 -2.9868708479
H -1.7776432678 1.7715743937 -1.8938151438
C -1.8010890514 4.2988879363 -0.3303618116
H -1.9306445281 5.0662093349 -1.0857255838
C -0.5740889860 3.6422647740 -0.2151768395
C -2.2965878294 -2.3681918875 -1.8675173067
C 4.8527561351 -1.3392939932 -1.3023925720
H 5.3577354508 -1.3185195808 -2.2638405498
C -2.8621443144 3.9806840076 0.5096079991
H -3.8092198604 4.5004448567 0.4014029966
C -1.6907251070 -3.6533828189 -2.3452265122
H -0.6008329724 -3.5716366085 -2.3923656920
H -2.0649113752 -3.9163248881 -3.3408493414
H -1.9312492506 -4.4749905810 -1.6673163235
C 0.6381577882 2.9514933883 -2.3146115575
H 0.6640123124 1.9165587251 -1.9549155582
```

H 1.5303794333 3.1300091605 -2.9245193263
H -0.2403877365 3.0666258967 -2.9595035067
C 2.7062617848 -1.5352763016 2.4506698547
H 1.7937838130 -0.9410576634 2.3217043948
C 0.5818976360 3.9572315193 -1.1551110189
H 1.5139690131 3.8505845050 -0.5895209152
C -2.5592009832 0.6283891505 3.2243799356
H -3.2789377512 1.3003653100 3.7039335036
H -2.3080204648 -0.1564761098 3.9421177208
H -3.0351408477 0.1581619906 2.3588968211
C -1.2871761767 1.3658545827 2.8085692602
H -0.5660848685 0.6132634349 2.4773300988
C -3.2690497258 -2.1892480586 -0.8270818252
C -3.9778597518 -3.2815698257 -0.0816230655
H -3.3448197898 -4.1658397789 0.0153795279
H -4.8978297669 -3.5758221515 -0.5988335451
H -4.2485686661 -2.9597731066 0.9270390447
C 3.7556973958 0.8520241378 -1.9198642146
H 3.1380432929 1.6067442078 -1.4192758421
C -0.9507254823 -0.7637748900 -3.4213162500
H -0.5310914486 0.2378816385 -3.2840677168
H -1.4025779410 -0.8061973122 -4.4187468862
H -0.1238976700 -1.4795377382 -3.3888402796
C -4.6778824356 -0.1749108371 0.0987363569
H -4.8349521161 -0.7668421228 1.0041191080
H -5.6362694619 -0.1243789445 -0.4311941352
H -4.4083281331 0.8394338532 0.4075286954
C -0.6829486436 2.1127496987 4.0066434888
H 0.2718920921 2.5804164384 3.7493860051
H -0.5087658575 1.4202812893 4.8353957200
H -1.3646529165 2.8972080619 4.3520214715
C 5.0462714934 1.5384478300 -2.3761177397
H 5.6130719206 1.9260992398 -1.5248398529
H 4.8148911245 2.3729904506 -3.0449073264
H 5.6926790644 0.8455621539 -2.9237158977
C 3.5127403388 -0.9288619279 3.6059967938
H 4.4436605491 -1.4868896077 3.7520111633
H 2.9389921324 -0.9697277066 4.5365384148
H 3.7711317381 0.1160870843 3.4104257142
C 2.2617590842 -2.9606165251 2.7845655122
H 1.7577245085 -3.4223739006 1.9307583858
H 1.5606593236 -2.9409503580 3.6221108695
H 3.1098525149 -3.5903258669 3.0729082197
C 0.5444792563 5.3870030251 -1.6987123702
H -0.2805334061 5.5269239878 -2.4040952593
H 1.4703686318 5.5992979671 -2.2398582281
H 0.4379369270 6.1231376824 -0.8970596742

8. References

(1) Varava, P.; Dong, Z.; Scopelliti, R.; Fadaei-Tirani, F.; Severin, K. *Nat. Chem.* **2021**, *13*, 1055–1060.

(2) Bergbreiter, D. E. *J. Am. Chem. Soc.* **1978**, *100*, 2126–2134.

(3) *CrysAlis^Pro^* Software System, Rigaku Oxford Diffraction (2024-25).

(4) Sheldrick, G. M. *Acta Crystallogr., Sect. A* **2015**, *71*, 3–8.

(5) Dolomanov, O. V.; Bourhis, L. J.; Gildea, R. J.; Howard, J. A. K.; Puschmann, H. *J. Appl. Crystallogr.* **2009**, *42*, 339–341.

(6) Bourhis, L. J.; Dolomanov, O. V.; Gildea, R. J.; Howard, J. A. K.; Puschmann, H. *Acta Crystallogr., Sect. A* **2015**, *71*, 59–71.

(7) Sheldrick, G. M. *Acta Crystallogr., Sect. C* **2015**, *71*, 3–8.

(8) Clark, R. C.; Reid, J. S. *Acta Crystallogr., Sect. A* **1995**, *51*, 887–897.

(9) Kuhs, W. F. *Aust. J. Phys*. **1988**, *41*, 369–382.

(10) Frisch, M. J.; Trucks, G. W.; Schlegel, H. B.; Scuseria, G. E.; Robb, M. A.; Cheeseman, J. R.; Scalmani, G.; Barone, V.; Petersson, G. A.; Nakatsuji, H.; Li, X.; Caricato, M.; Marenich, A. V.; Bloino, J.; Janesko, B. G.; Gomperts, R.; Mennucci, B.; Hratchian, H. P.; Ortiz, J. V.; Izmaylov, A. F.; Sonnenberg, J. L.; Williams-Young, D.; Ding, F.; Lipparini, F.; Egidi, F.; Goings, J.; Peng, B.; Petrone, A.; Henderson, T.; Ranasinghe, D.; Zakrzewski, V. G.; Gao, J.; Rega, N.; Zheng, G.; Liang, W.; Hada, M.; Ehara, M.; Toyota, K.; Fukuda, R.; Hasegawa, J.; Ishida, M.; Nakajima, T.; Honda, Y.; Kitao, O.; Nakai, H.; Vreven, T.; Throssell, K.; Montgomery, J. A., Jr.; Peralta, J. E.; Ogliaro, F.; Bearpark, M. J.; Heyd, J. J.; Brothers, E. N.; Kudin, K. N.; Staroverov, V. N.; Keith, T. A.; Kobayashi, R.; Normand, J.; Raghavachari, K.; Rendell, A. P.; Burant, J. C.; Iyengar, S. S.; Tomasi, J.; Cossi, M.; Millam, J. M.; Klene, M.; Adamo, C.; Cammi, R.; Ochterski, J. W.; Martin, R. L.; Morokuma, K.; Farkas, O.; Foresman, J. B.; Fox, D. J. *Gaussian 16*, Gaussian, Inc., Wallingford, CT, 2019.

(11) Zhao, Y.; Schultz, N. E.; Truhlar, D. G. *J. Chem. Theory Comput.* **2006**, *2*, 364−382.

(12) Pritchard, B. P.; Altarawy, D.; Didier, B.; Gibson, T. D.; Windus, T. L. *J. Chem. Inf. Model.* **2019**, *59*, 4814–4820.

(13) Hay, P. J.; Wadt, W. R. *J. Chem. Phys.* **1985**, *82*, 270–283.

(14) Ditchfield, R.; Hehre, W. J.; Pople, J. A. *J. Chem. Phys.* **1971**, *54*, 724–728.

(15) Franci, M. M.; Pietro, W. J.; Hehre, W. J.; Binkley, J. S.; Gordon, M. S.; DeFrees, D. J.; Pople, J. A. *J. Chem. Phys.* **1982**, *77*, 3654–3665.

(16) Gordon, M. S.; Binkley, J. S.; Pople, J. A.; Pietro, W. J. *J. Am. Chem. Soc.* **1982**, *104*, 2797–2803.

(17) Hariharan, P. C.; Pople, J. A. *Theor. Chim. Acta* **1983**, *28*, 213–222.

(18) Hehre, W. J.; Ditchfield, R.; Pople, J. A. *J. Chem. Phys.* **1972**, *56*, 2257–2261.

(19) Reed, A. E.; Curtiss, L. A.; Weinhold, F. *Chem. Rev.* **1988**, *88*, 899–926.

(20) Glendening, E. D.; Reed, A. E.; Carpenter, J. E.; Weinhold, F. NBO Version 3.1.
